# Supplementary figures and images for: Skin-lightening products and Jordanian women: Beliefs and practice. A cross-sectional study
Source: PLoS One. 2023 Nov 21;18(11):e0293896. doi: 10.1371/journal.pone.0293896 (PMC10662732; doi:10.1371/journal.pone.0293896)

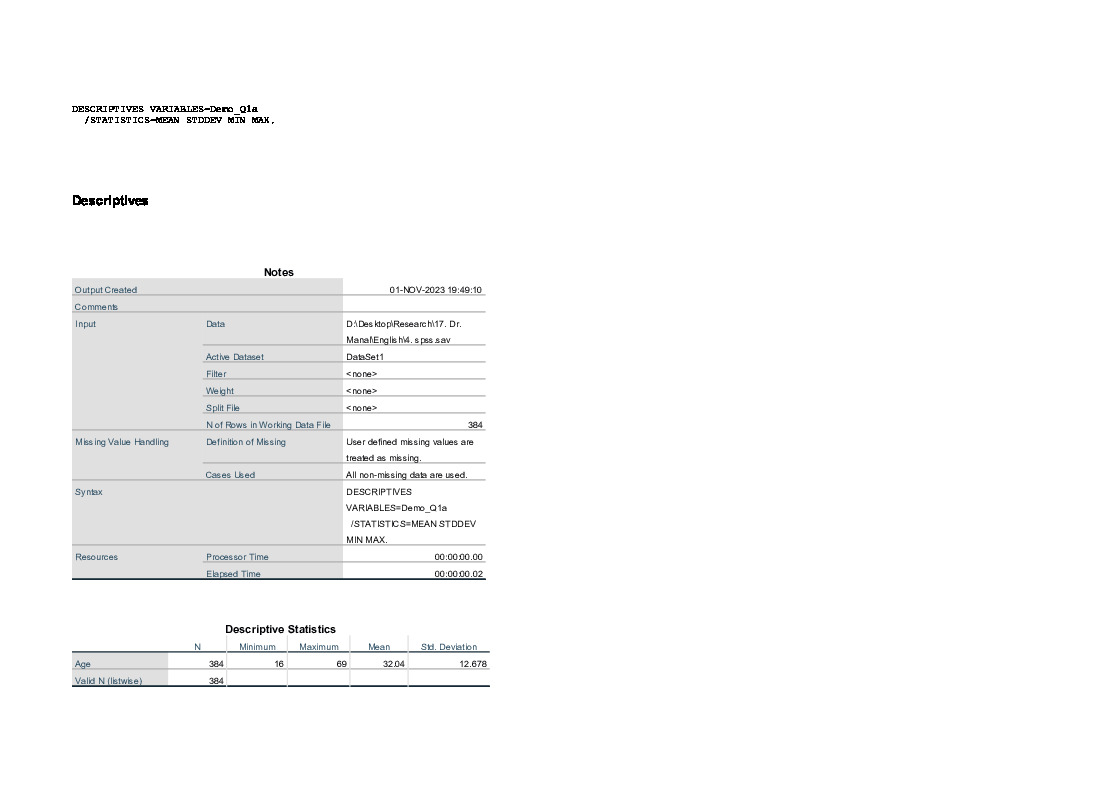

Supplement: S1 Fig — (ZIP) [file pone.0293896.s001.zip › Outputs - word file 00.tiff]

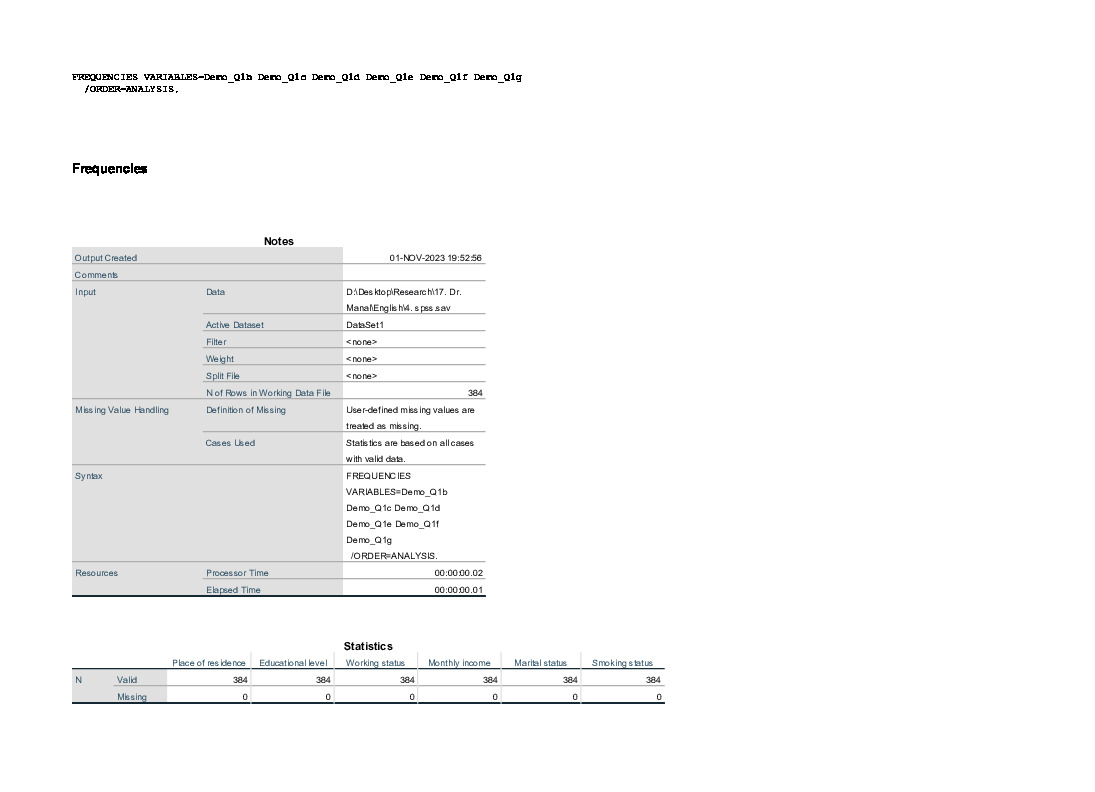

Supplement: S1 Fig — (ZIP) [file pone.0293896.s001.zip › Outputs - word file 01.tiff]

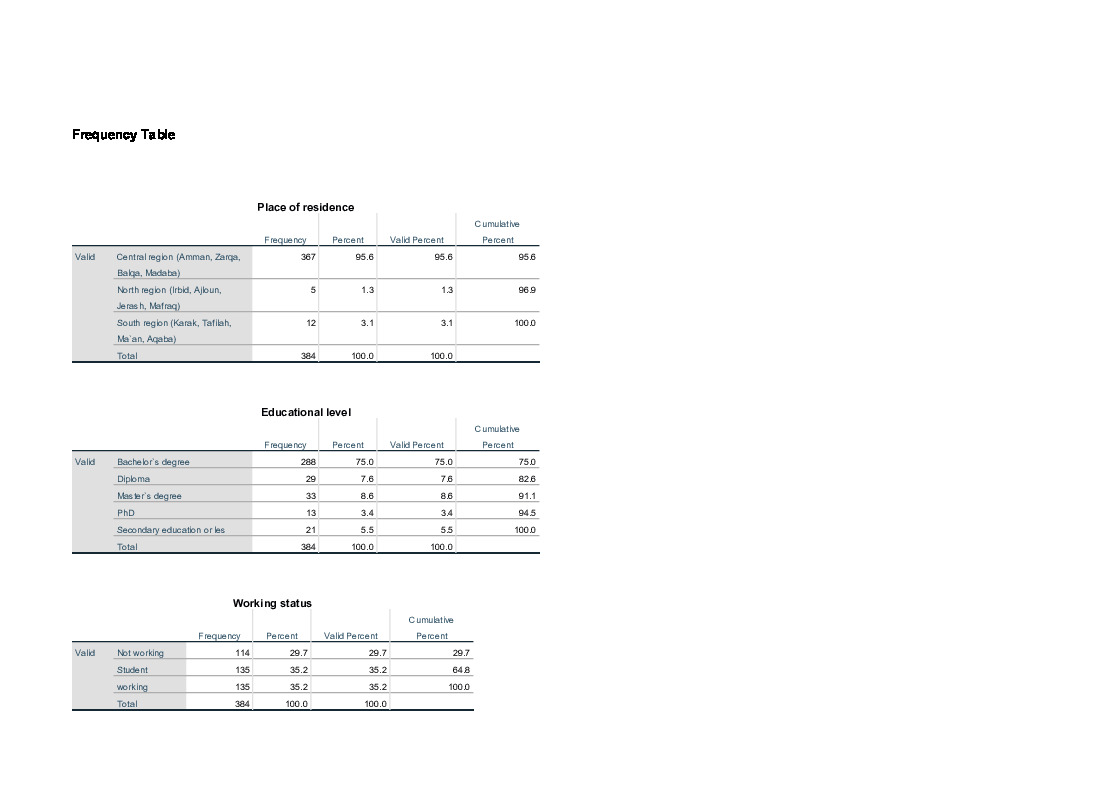

Supplement: S1 Fig — (ZIP) [file pone.0293896.s001.zip › Outputs - word file 02.tiff]

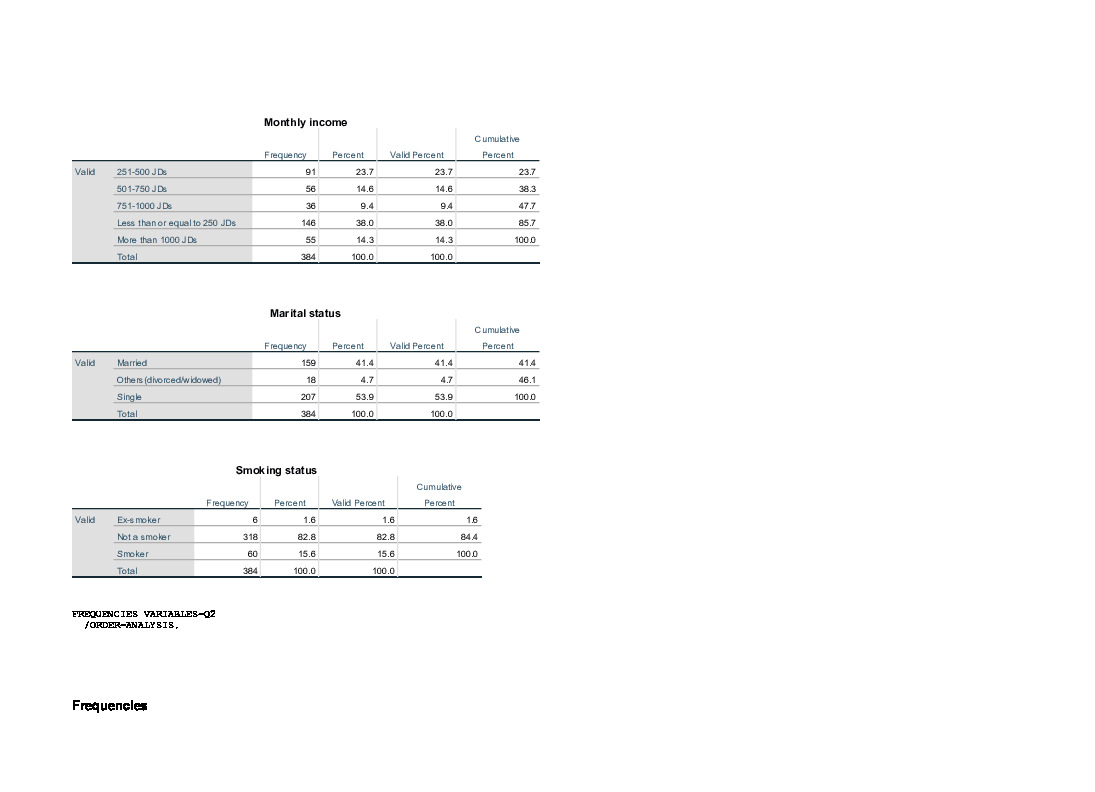

Supplement: S1 Fig — (ZIP) [file pone.0293896.s001.zip › Outputs - word file 03.tiff]

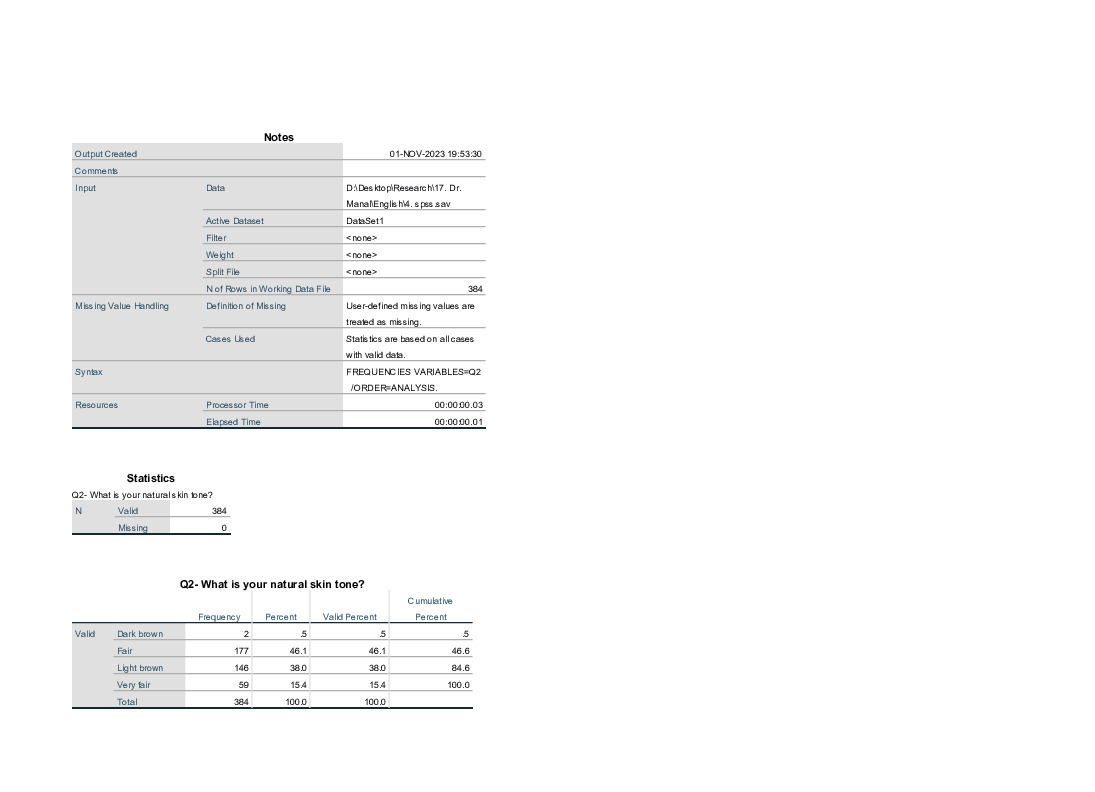

Supplement: S1 Fig — (ZIP) [file pone.0293896.s001.zip › Outputs - word file 04.tiff]

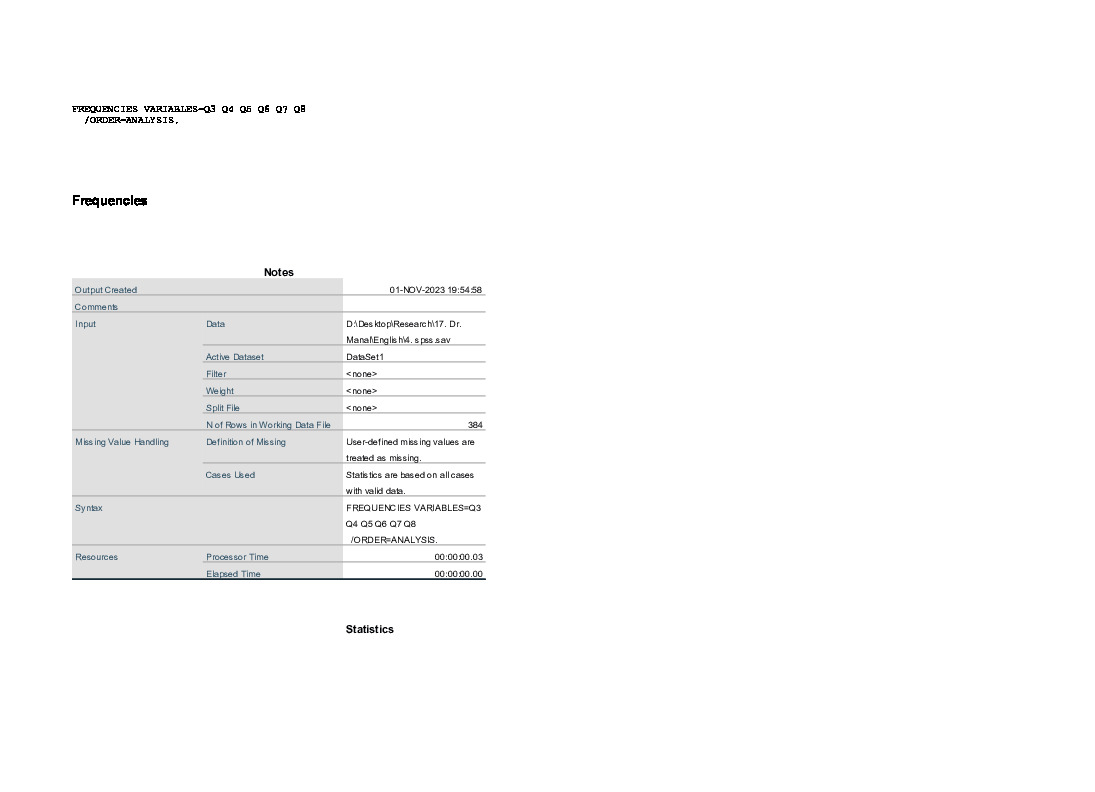

Supplement: S1 Fig — (ZIP) [file pone.0293896.s001.zip › Outputs - word file 05.tiff]

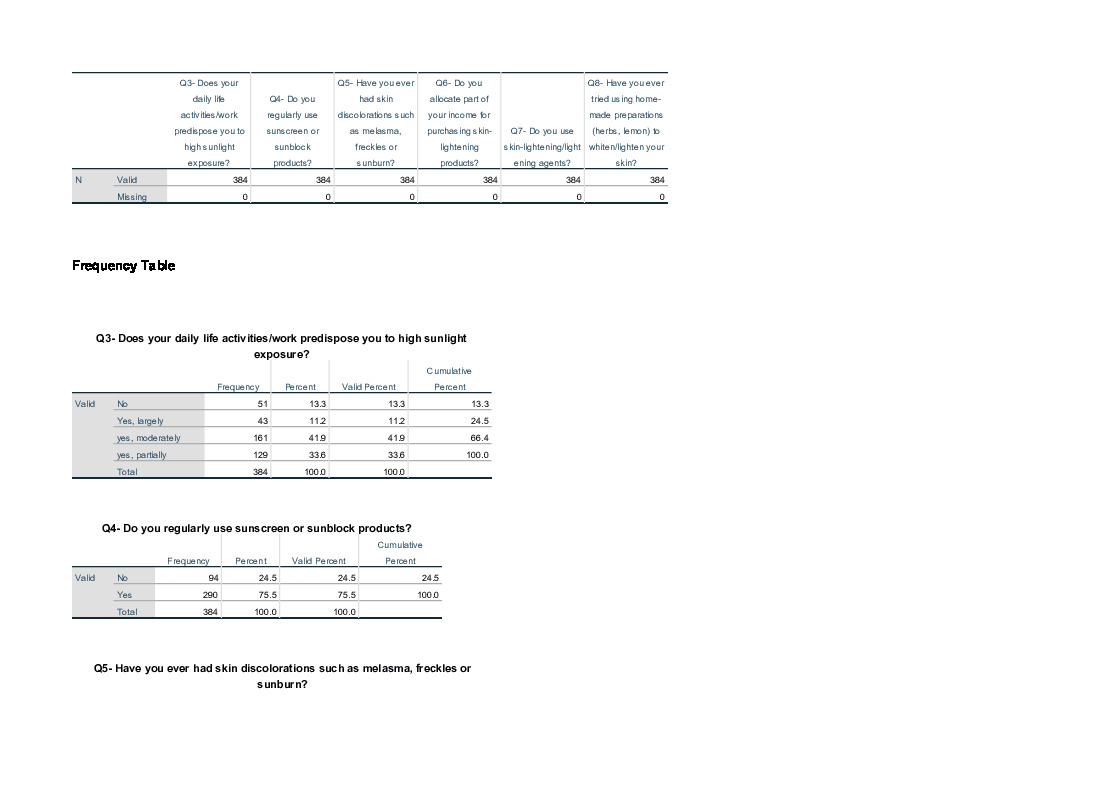

Supplement: S1 Fig — (ZIP) [file pone.0293896.s001.zip › Outputs - word file 06.tiff]

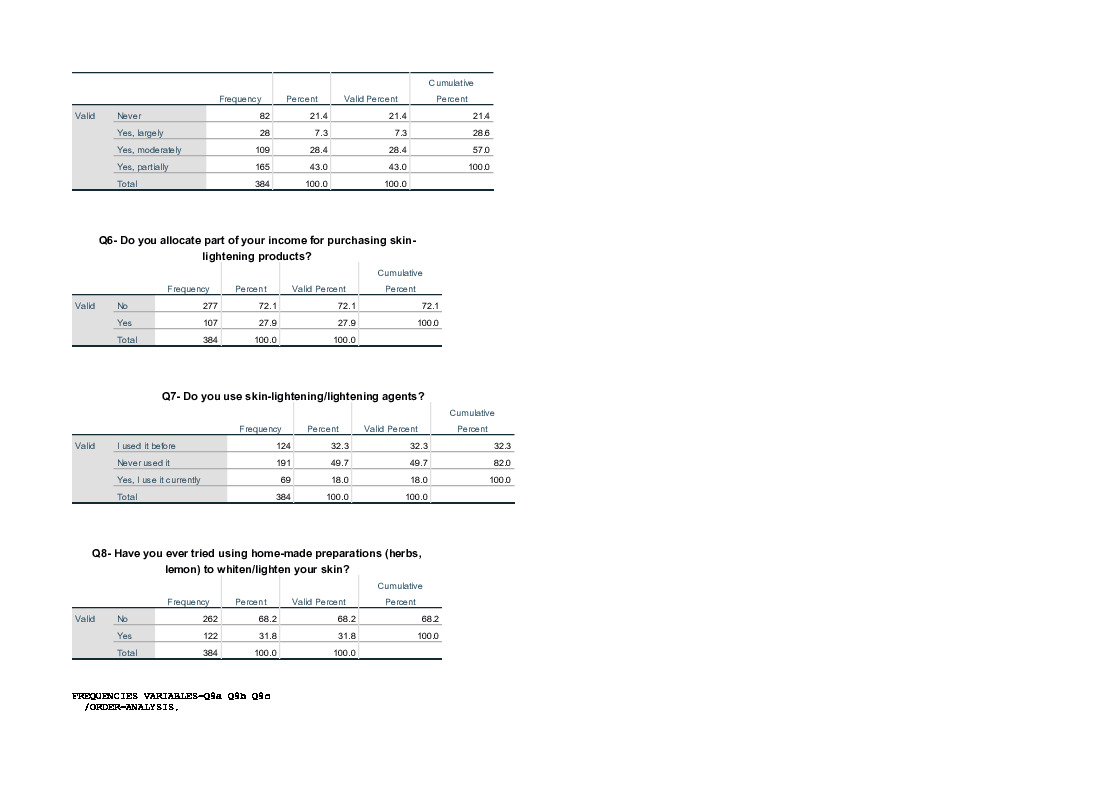

Supplement: S1 Fig — (ZIP) [file pone.0293896.s001.zip › Outputs - word file 07.tiff]

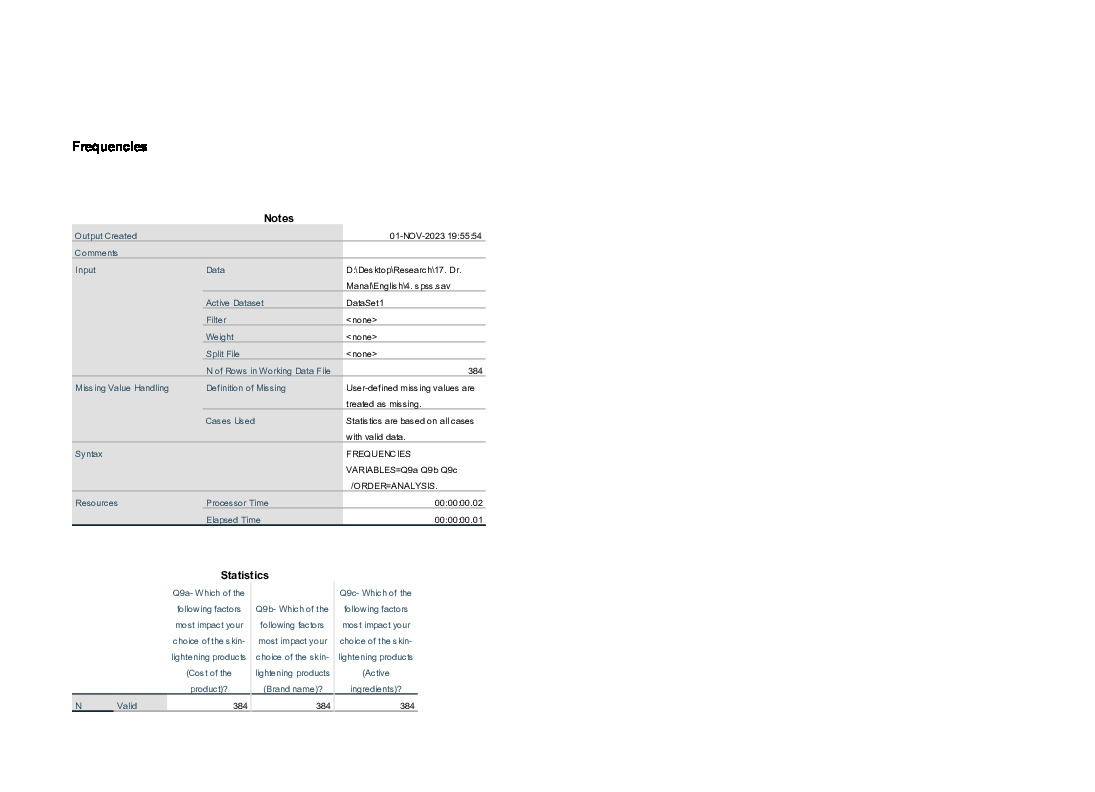

Supplement: S1 Fig — (ZIP) [file pone.0293896.s001.zip › Outputs - word file 08.tiff]

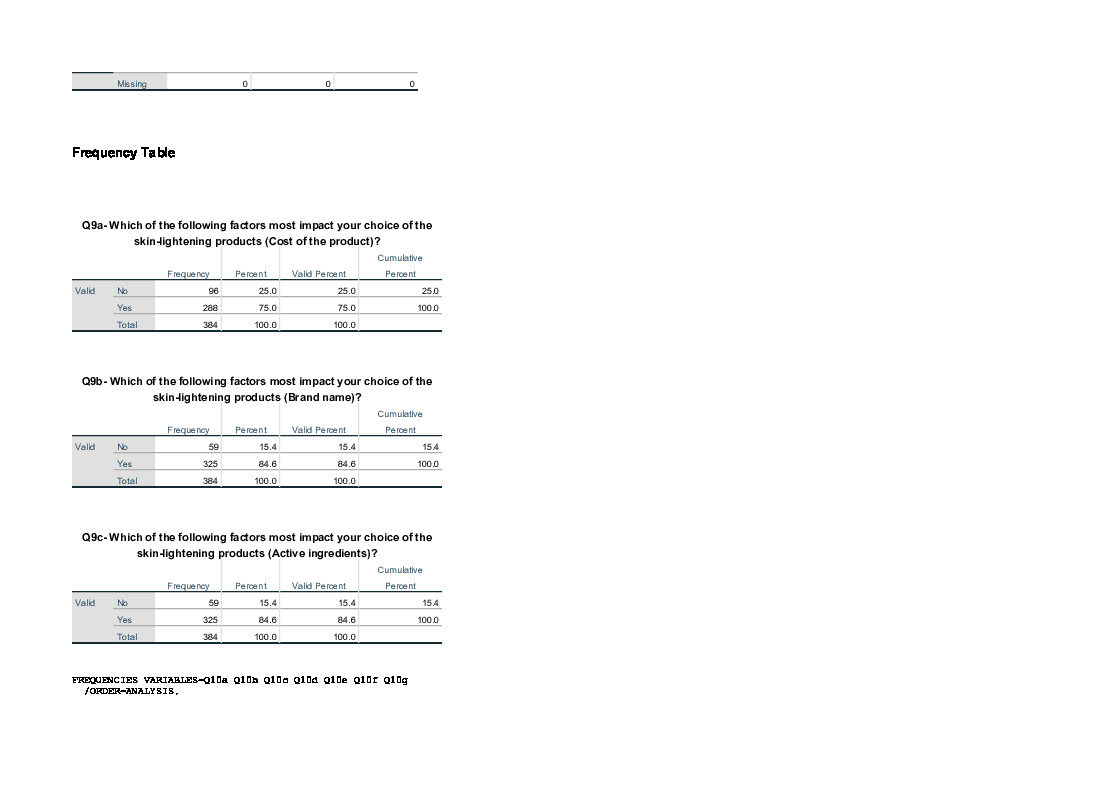

Supplement: S1 Fig — (ZIP) [file pone.0293896.s001.zip › Outputs - word file 09.tiff]

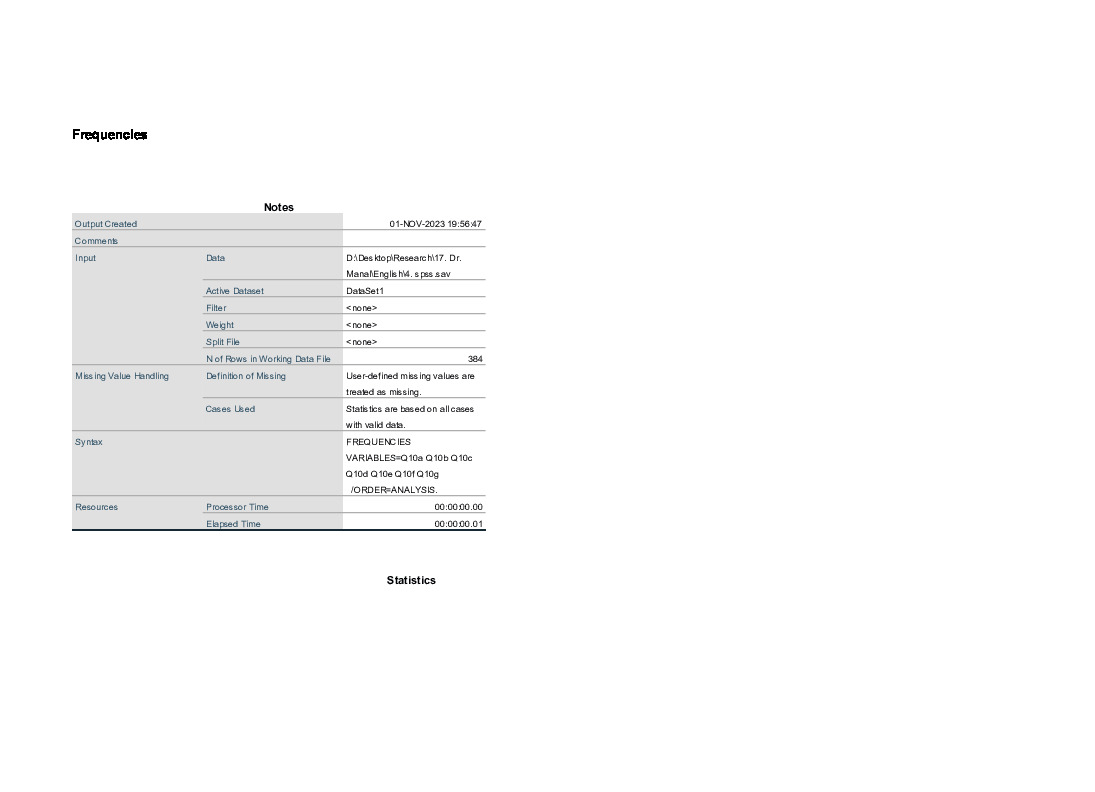

Supplement: S1 Fig — (ZIP) [file pone.0293896.s001.zip › Outputs - word file 10.tiff]

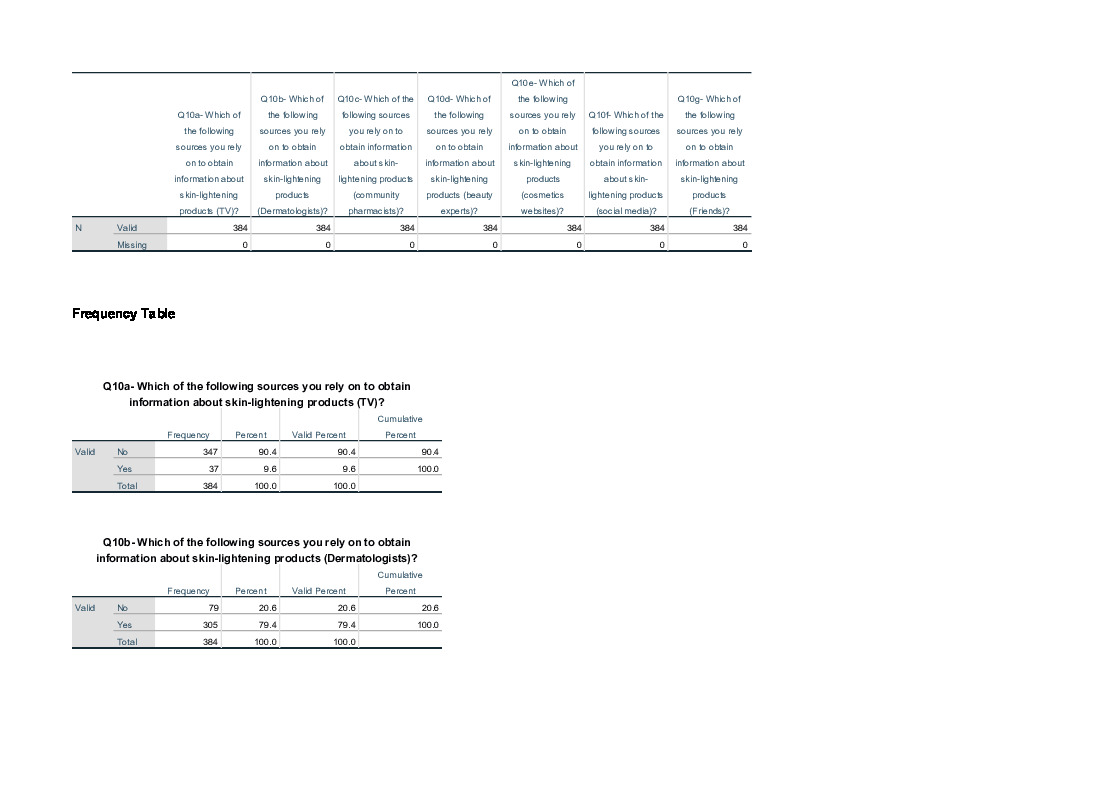

Supplement: S1 Fig — (ZIP) [file pone.0293896.s001.zip › Outputs - word file 11.tiff]

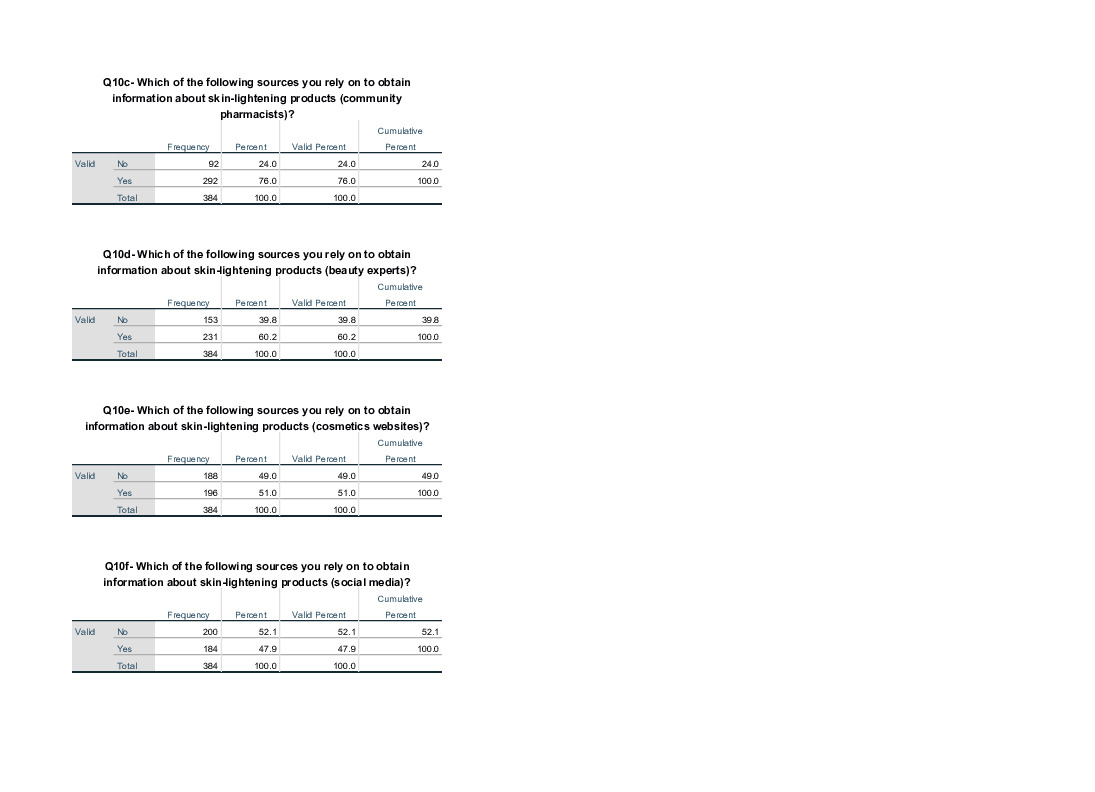

Supplement: S1 Fig — (ZIP) [file pone.0293896.s001.zip › Outputs - word file 12.tiff]

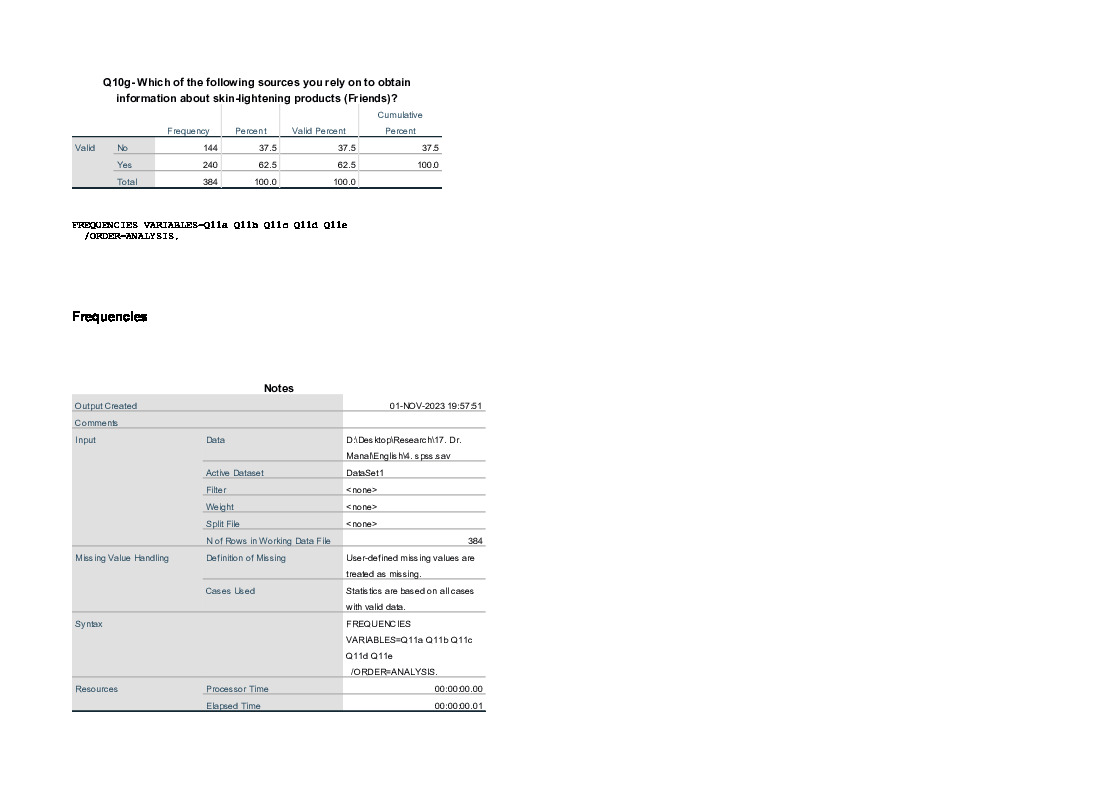

Supplement: S1 Fig — (ZIP) [file pone.0293896.s001.zip › Outputs - word file 13.tiff]

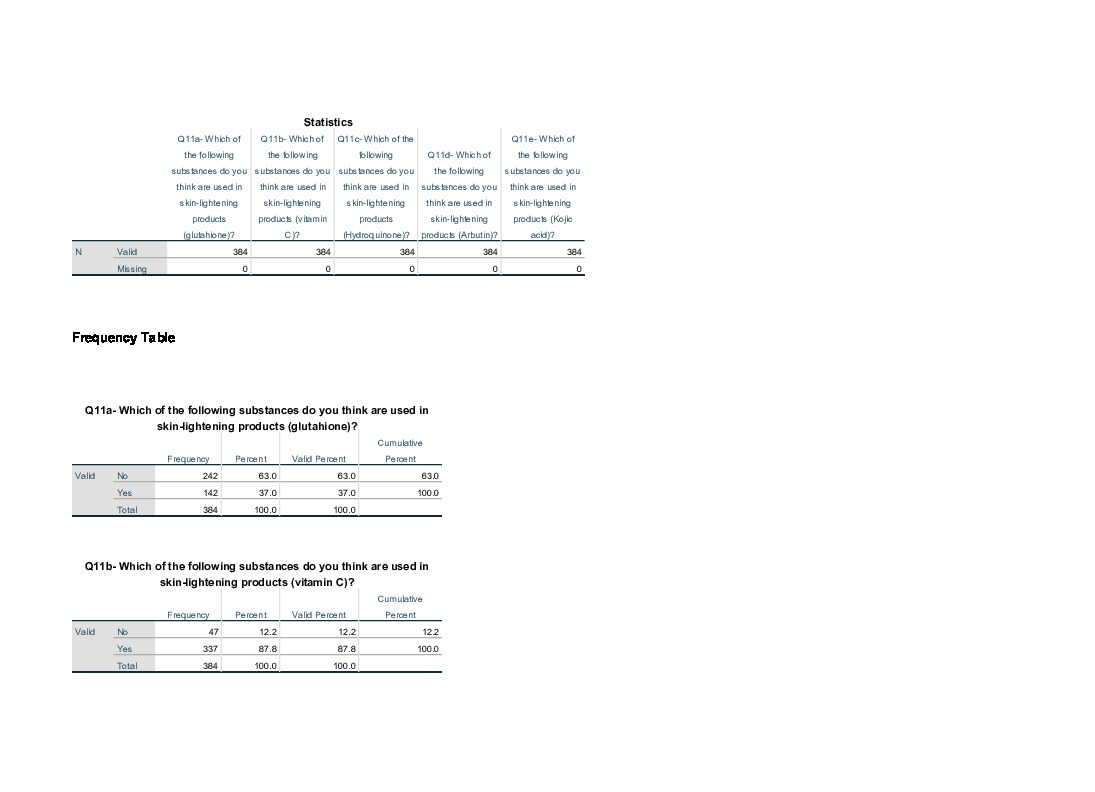

Supplement: S1 Fig — (ZIP) [file pone.0293896.s001.zip › Outputs - word file 14.tiff]

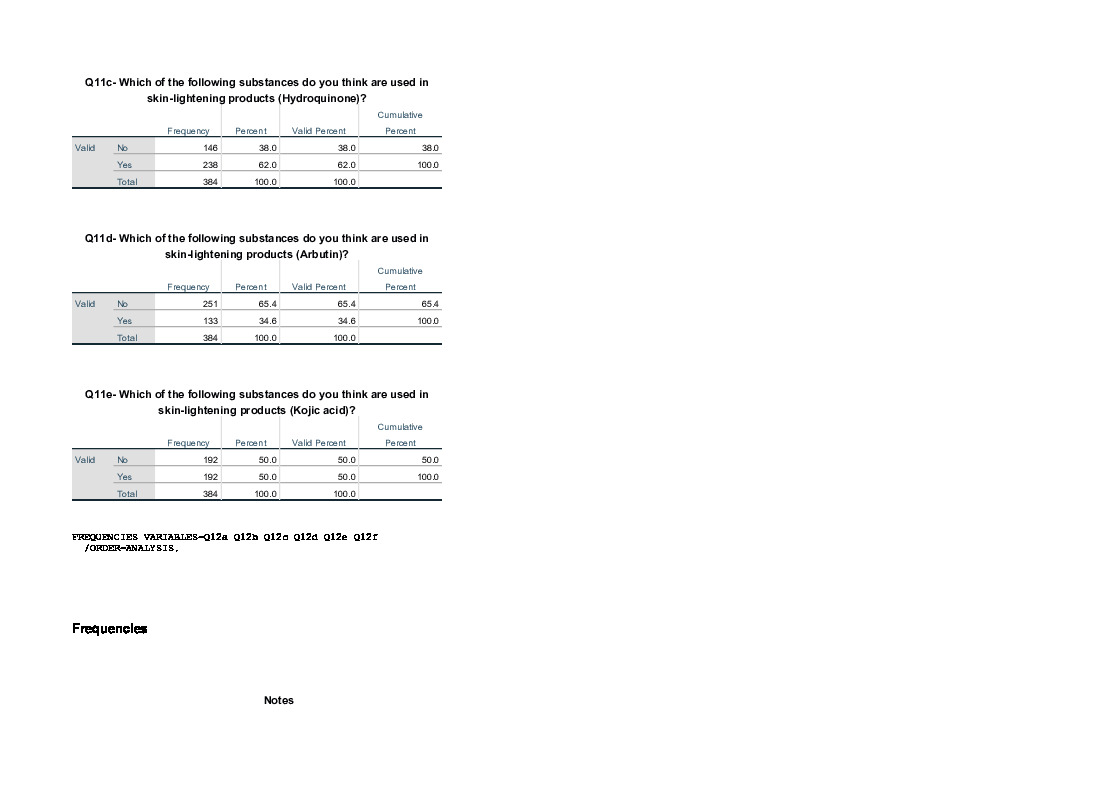

Supplement: S1 Fig — (ZIP) [file pone.0293896.s001.zip › Outputs - word file 15.tiff]

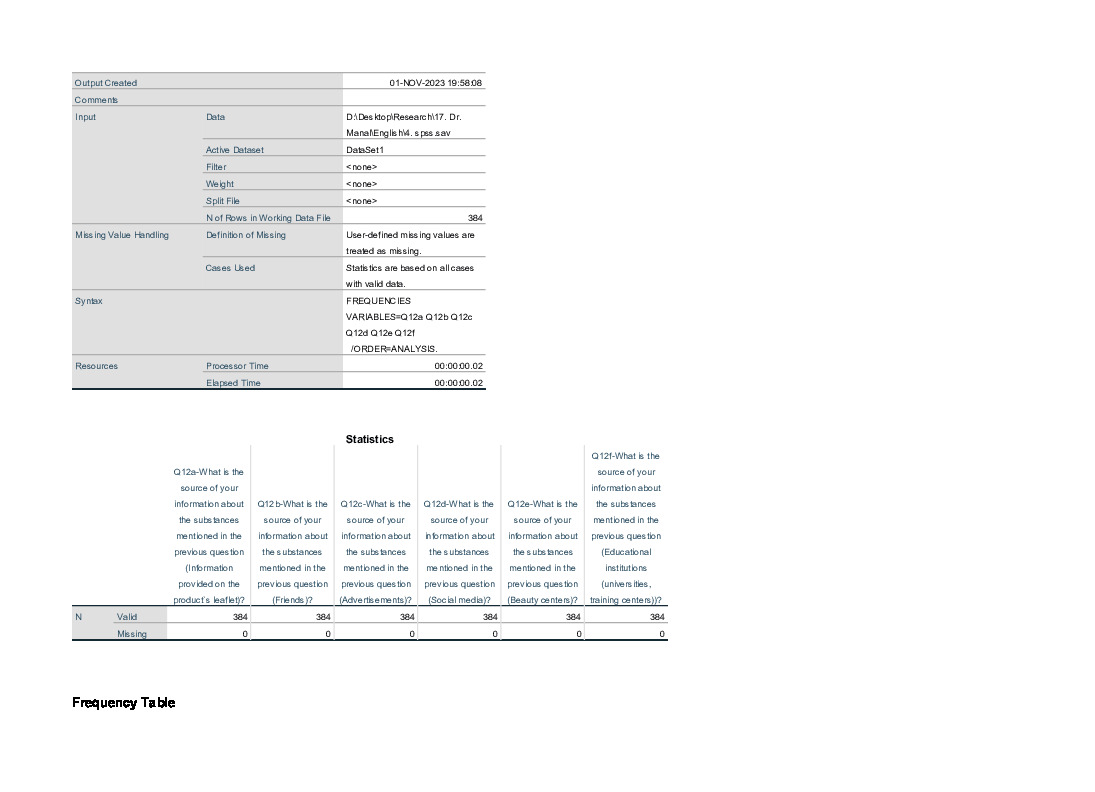

Supplement: S1 Fig — (ZIP) [file pone.0293896.s001.zip › Outputs - word file 16.tiff]

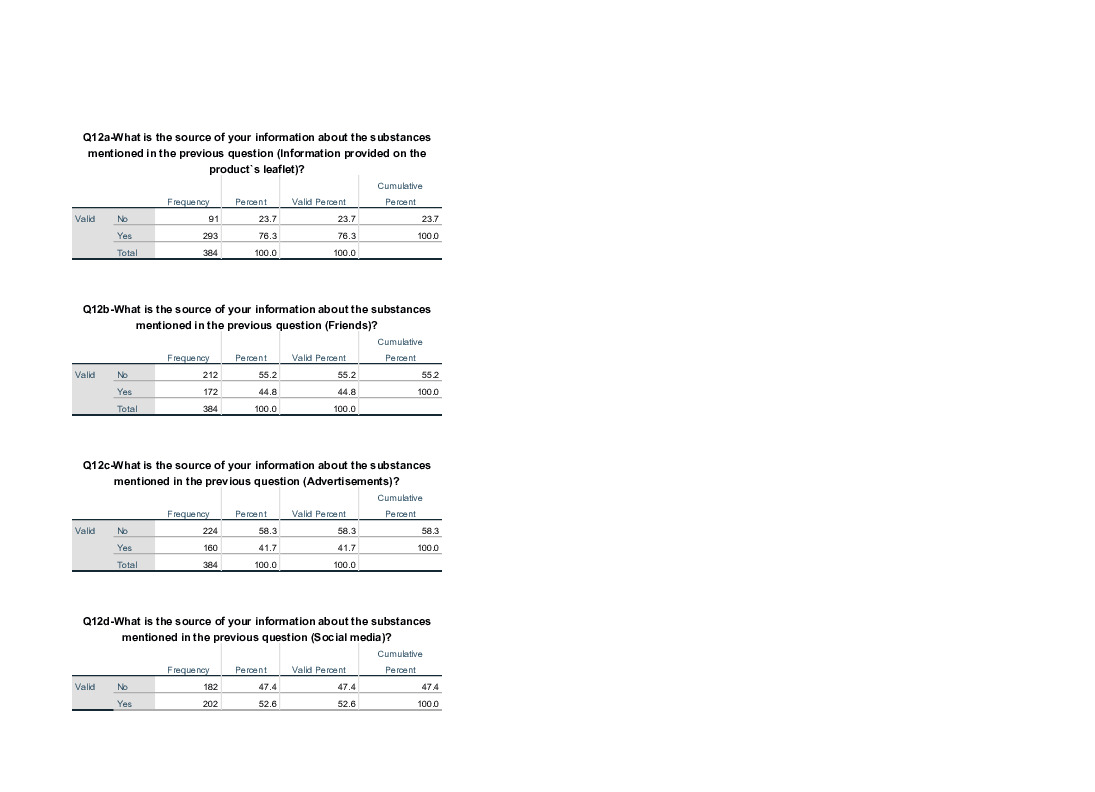

Supplement: S1 Fig — (ZIP) [file pone.0293896.s001.zip › Outputs - word file 17.tiff]

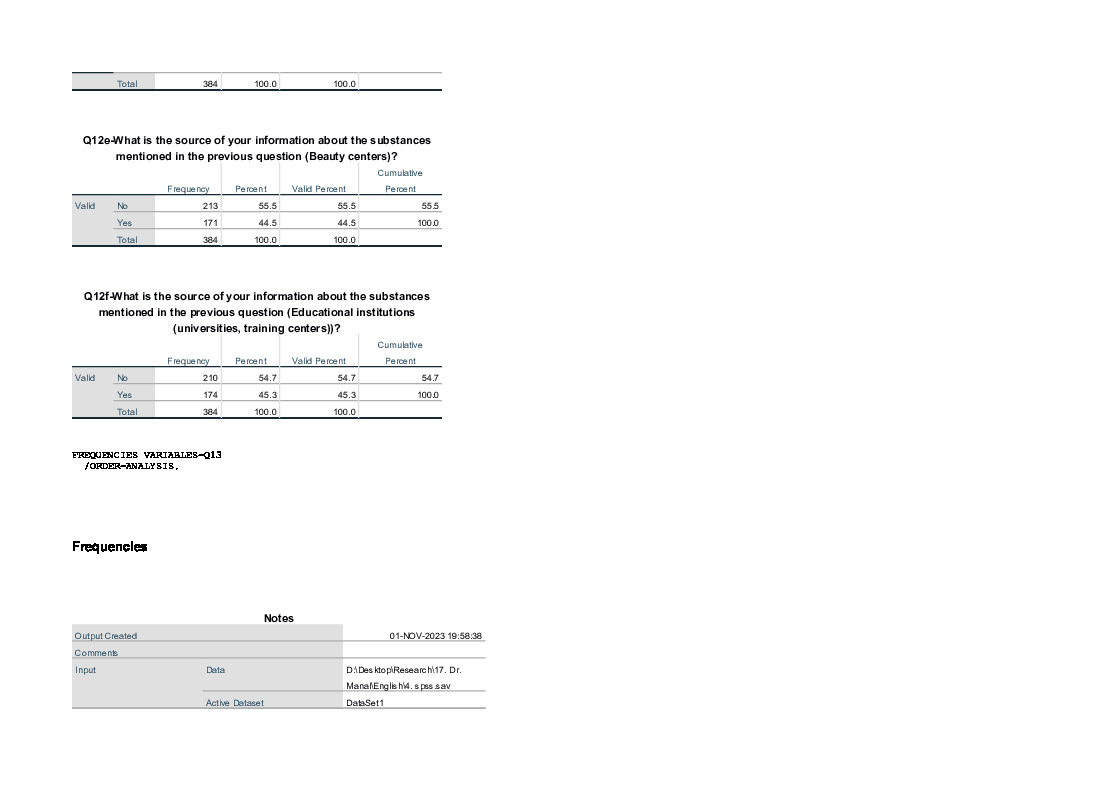

Supplement: S1 Fig — (ZIP) [file pone.0293896.s001.zip › Outputs - word file 18.tiff]

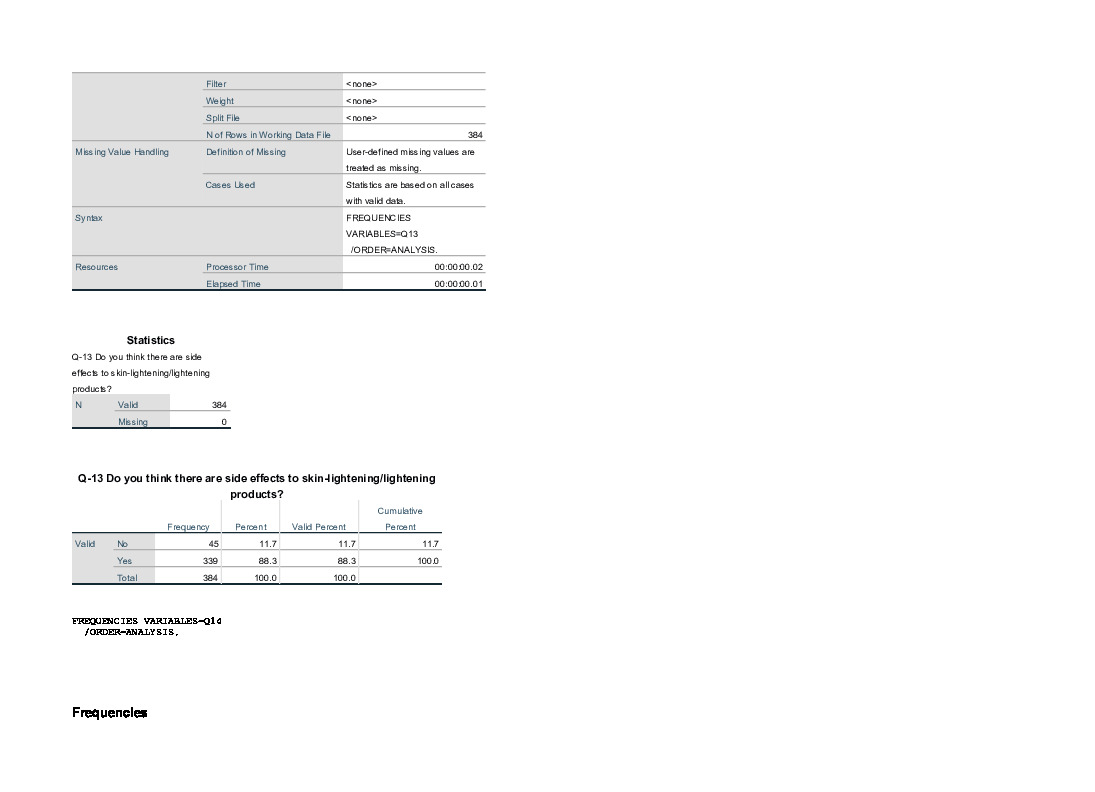

Supplement: S1 Fig — (ZIP) [file pone.0293896.s001.zip › Outputs - word file 19.tiff]

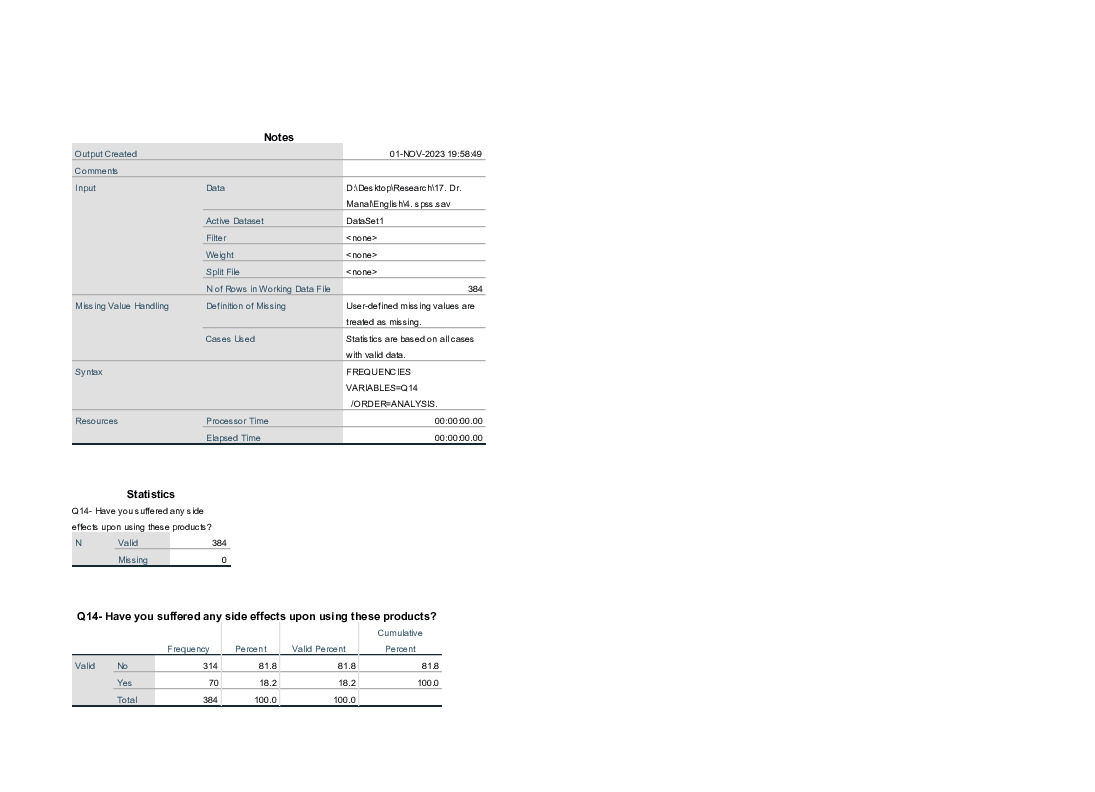

Supplement: S1 Fig — (ZIP) [file pone.0293896.s001.zip › Outputs - word file 20.tiff]

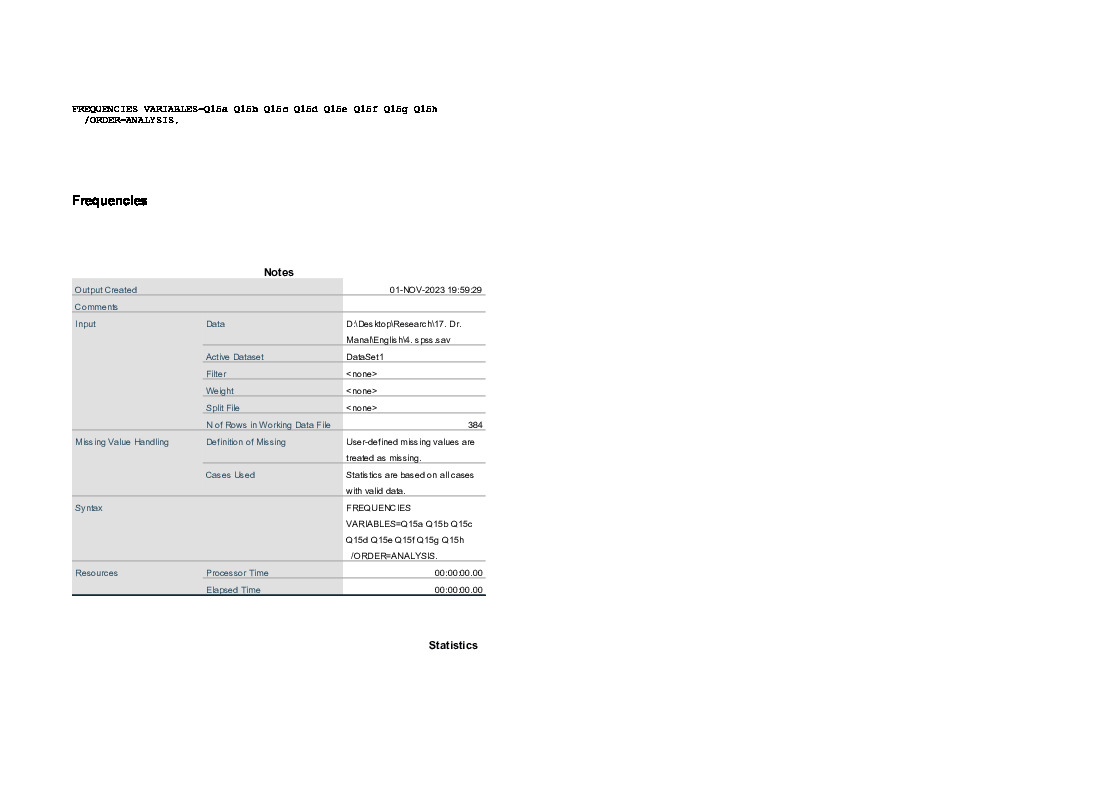

Supplement: S1 Fig — (ZIP) [file pone.0293896.s001.zip › Outputs - word file 21.tiff]

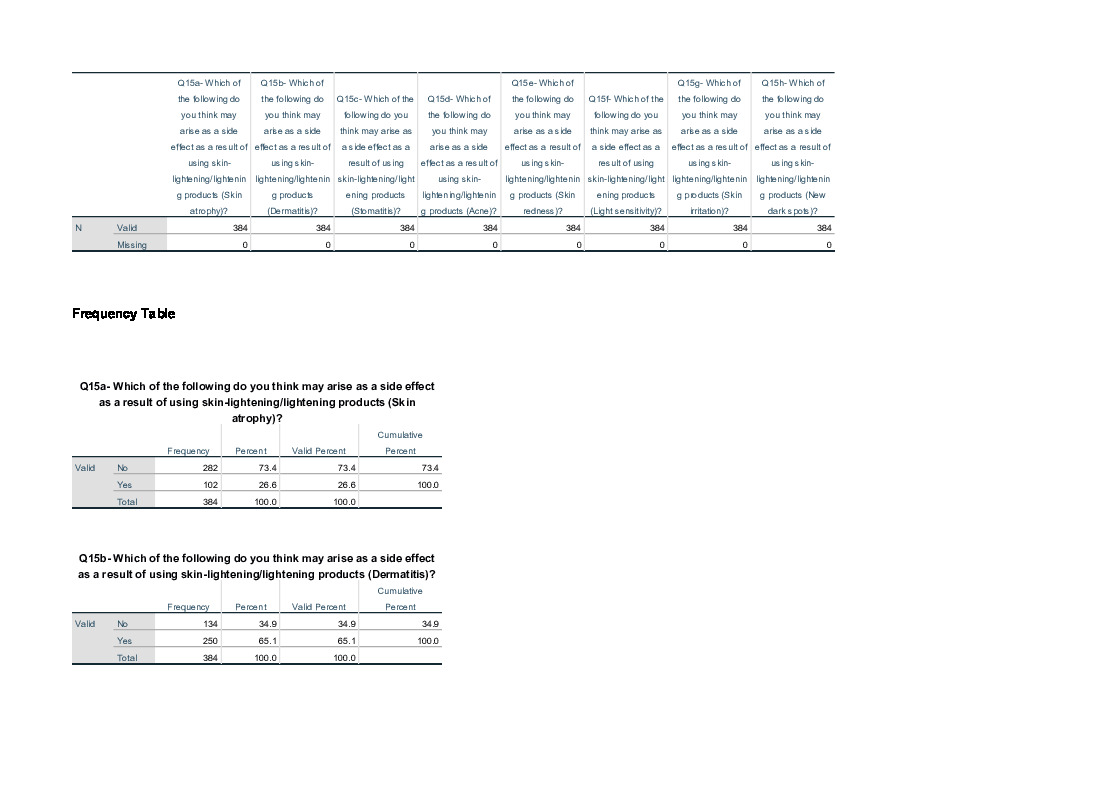

Supplement: S1 Fig — (ZIP) [file pone.0293896.s001.zip › Outputs - word file 22.tiff]

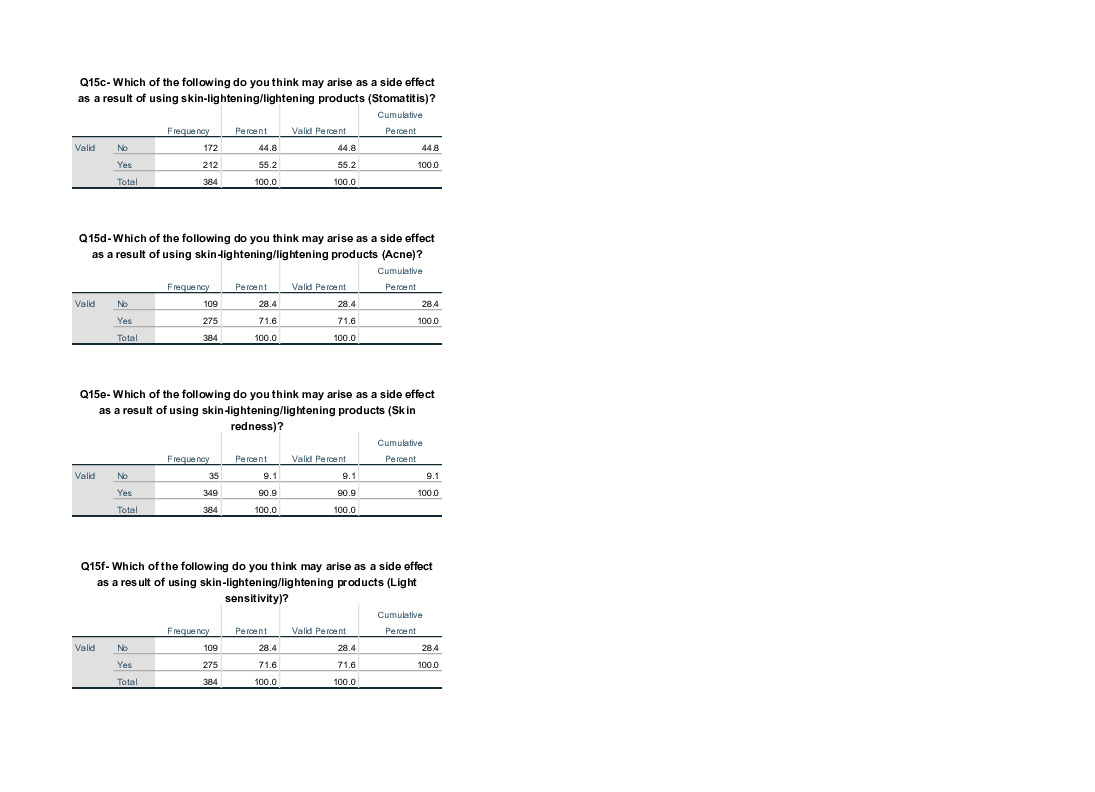

Supplement: S1 Fig — (ZIP) [file pone.0293896.s001.zip › Outputs - word file 23.tiff]

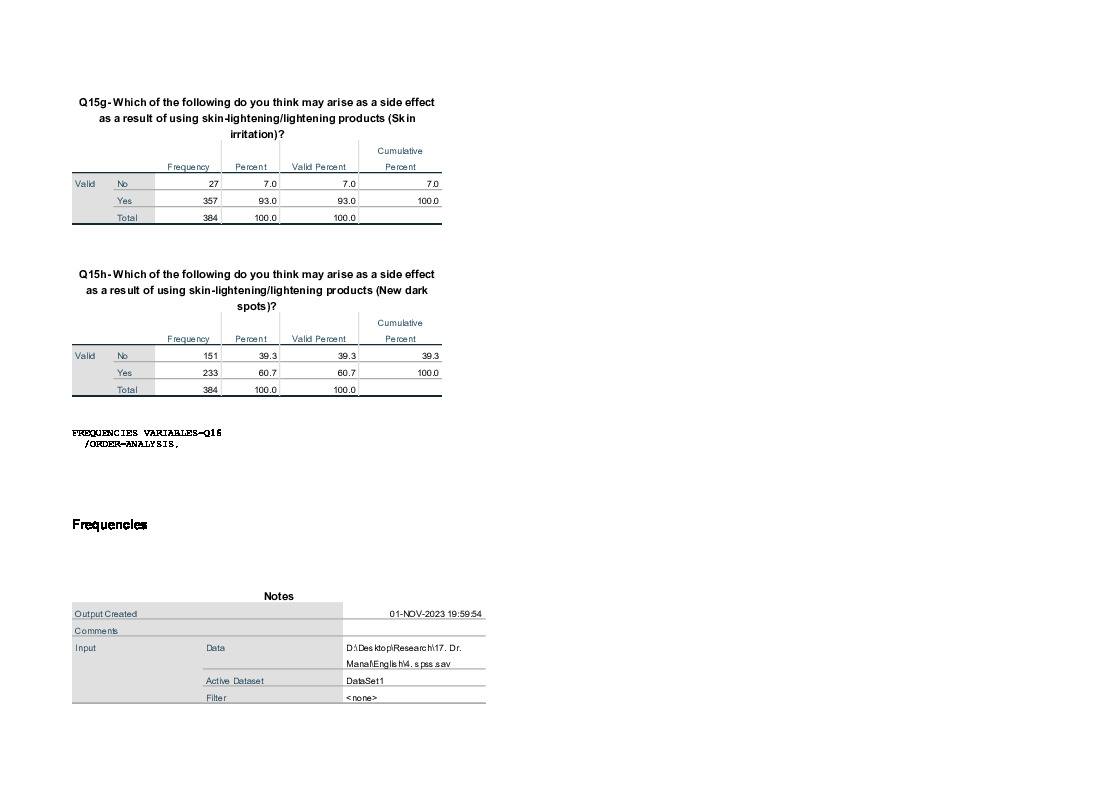

Supplement: S1 Fig — (ZIP) [file pone.0293896.s001.zip › Outputs - word file 24.tiff]

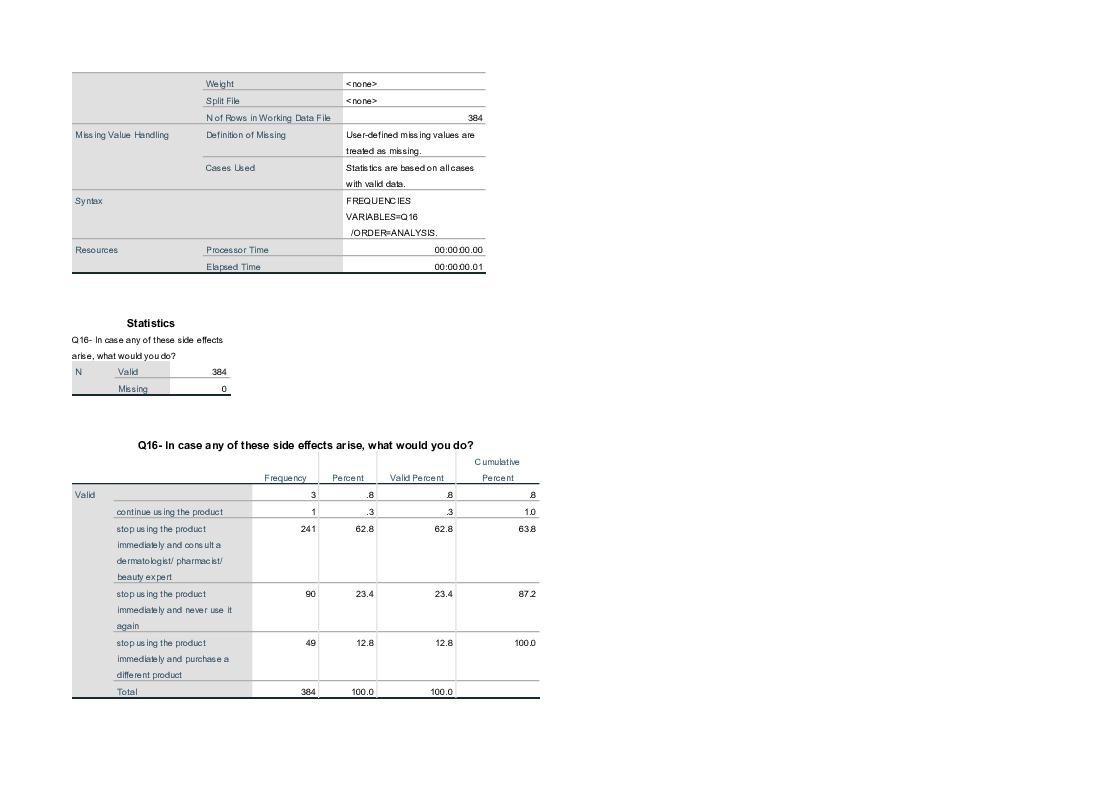

Supplement: S1 Fig — (ZIP) [file pone.0293896.s001.zip › Outputs - word file 25.tiff]

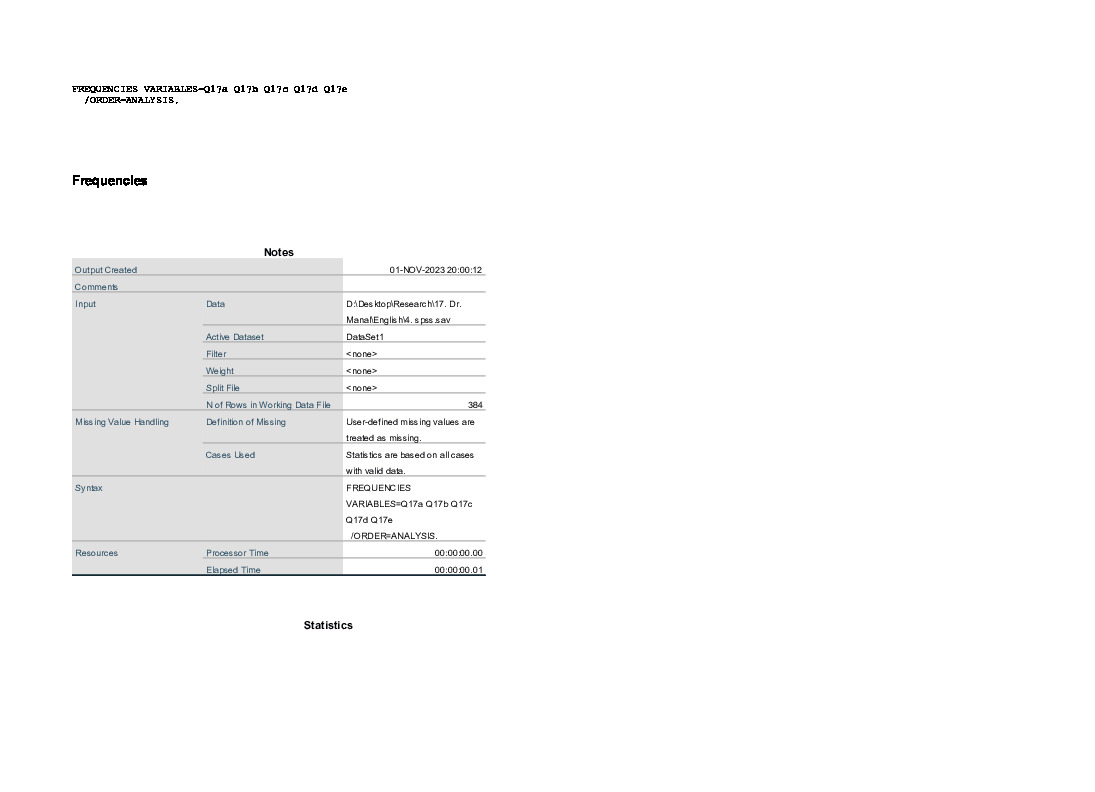

Supplement: S1 Fig — (ZIP) [file pone.0293896.s001.zip › Outputs - word file 26.tiff]

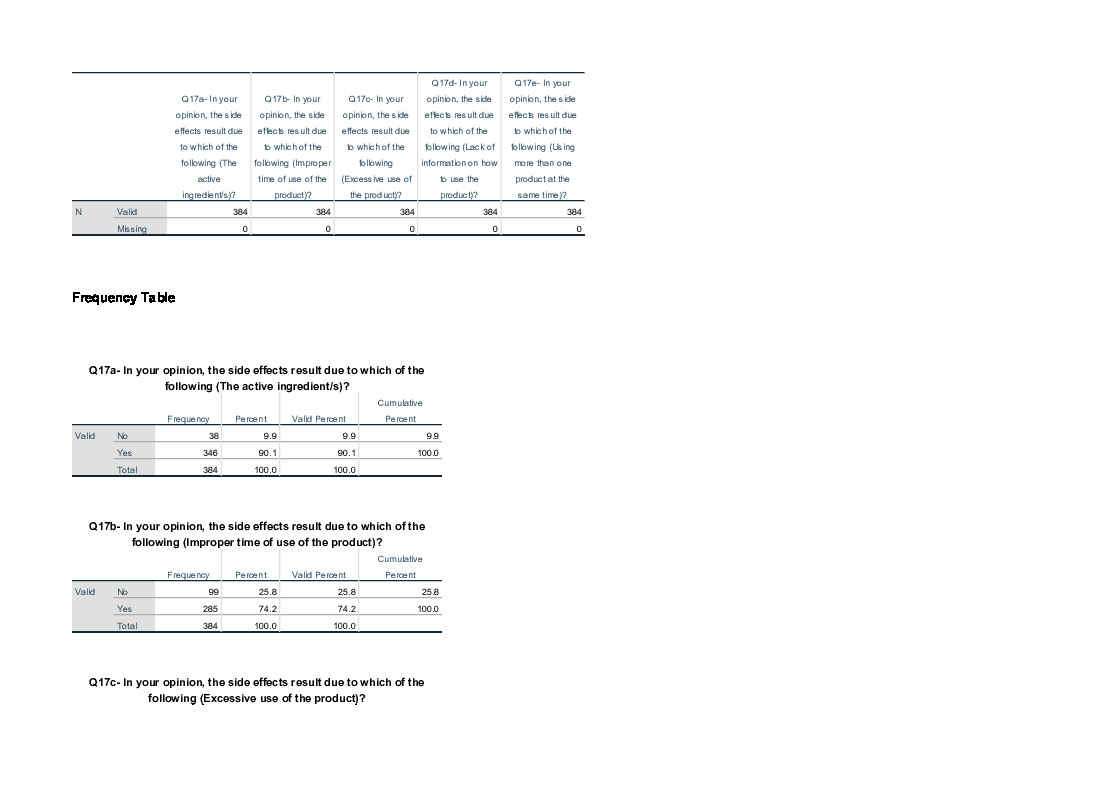

Supplement: S1 Fig — (ZIP) [file pone.0293896.s001.zip › Outputs - word file 27.tiff]

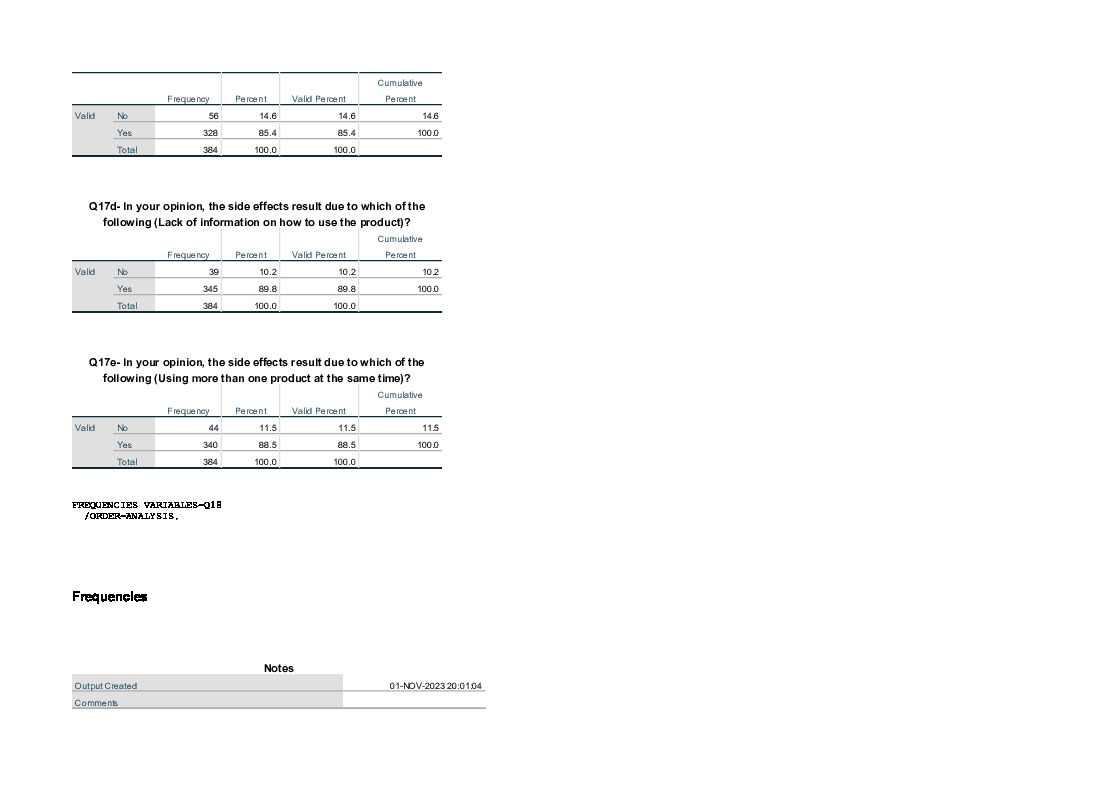

Supplement: S1 Fig — (ZIP) [file pone.0293896.s001.zip › Outputs - word file 28.tiff]

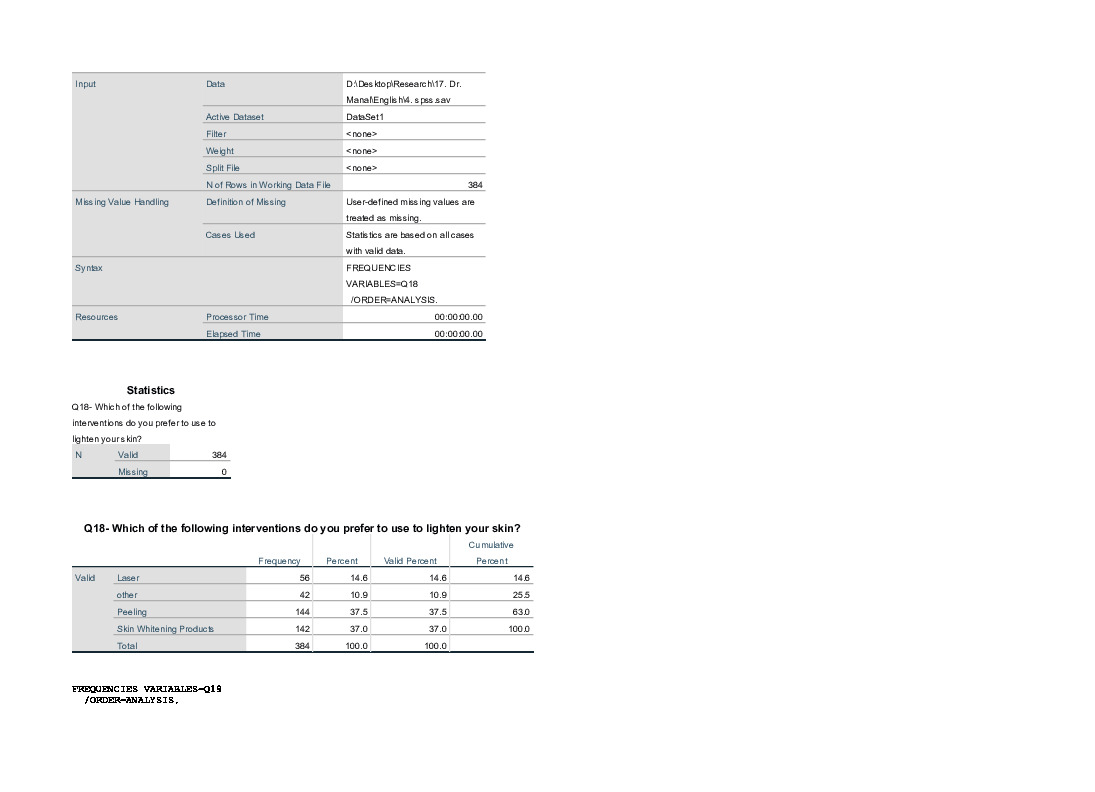

Supplement: S1 Fig — (ZIP) [file pone.0293896.s001.zip › Outputs - word file 29.tiff]

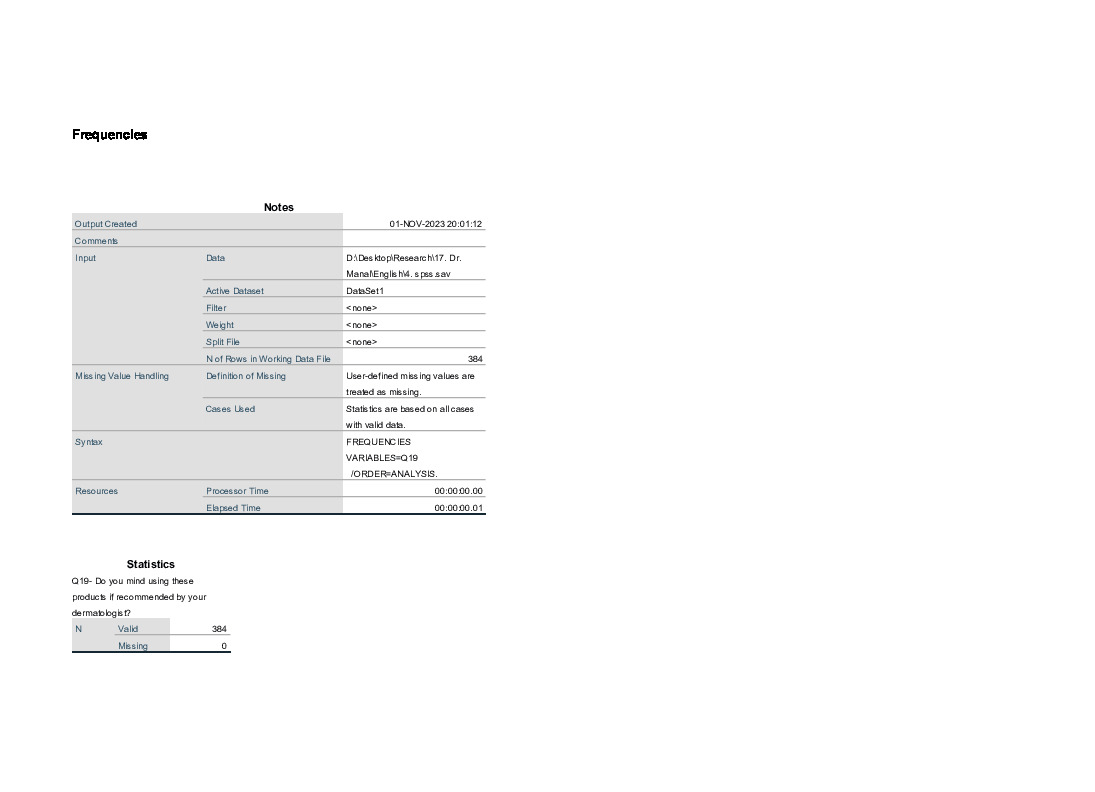

Supplement: S1 Fig — (ZIP) [file pone.0293896.s001.zip › Outputs - word file 30.tiff]

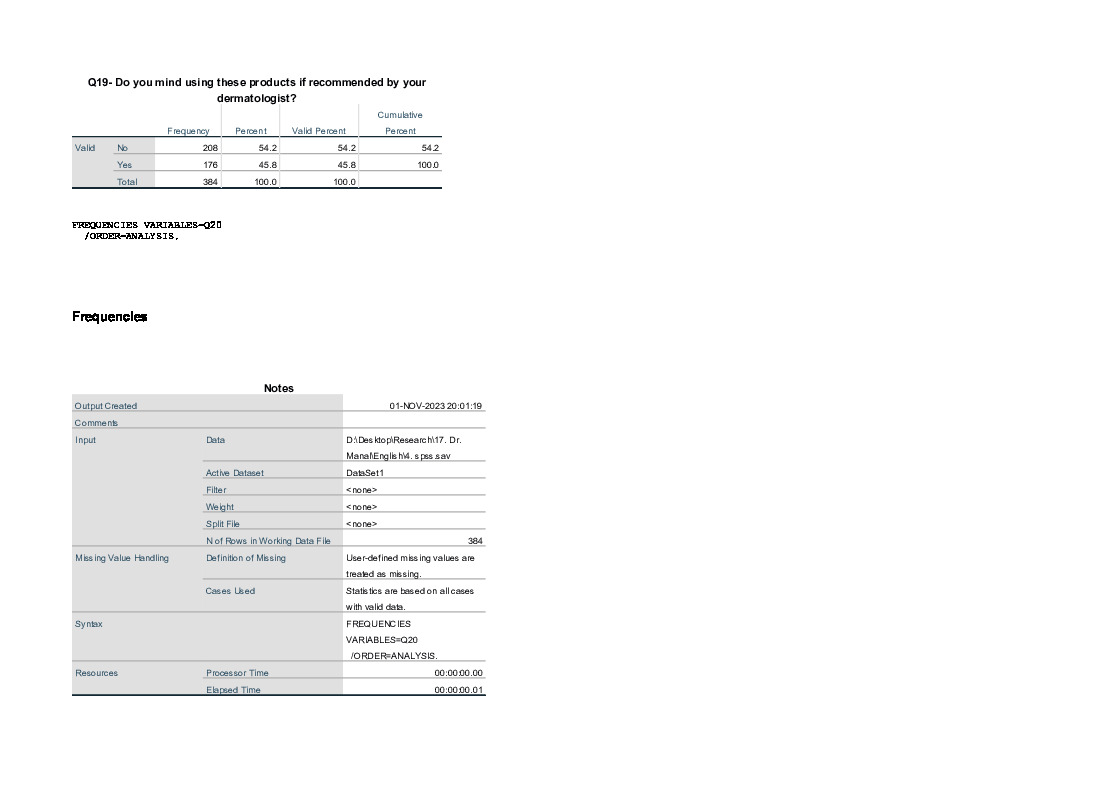

Supplement: S1 Fig — (ZIP) [file pone.0293896.s001.zip › Outputs - word file 31.tiff]

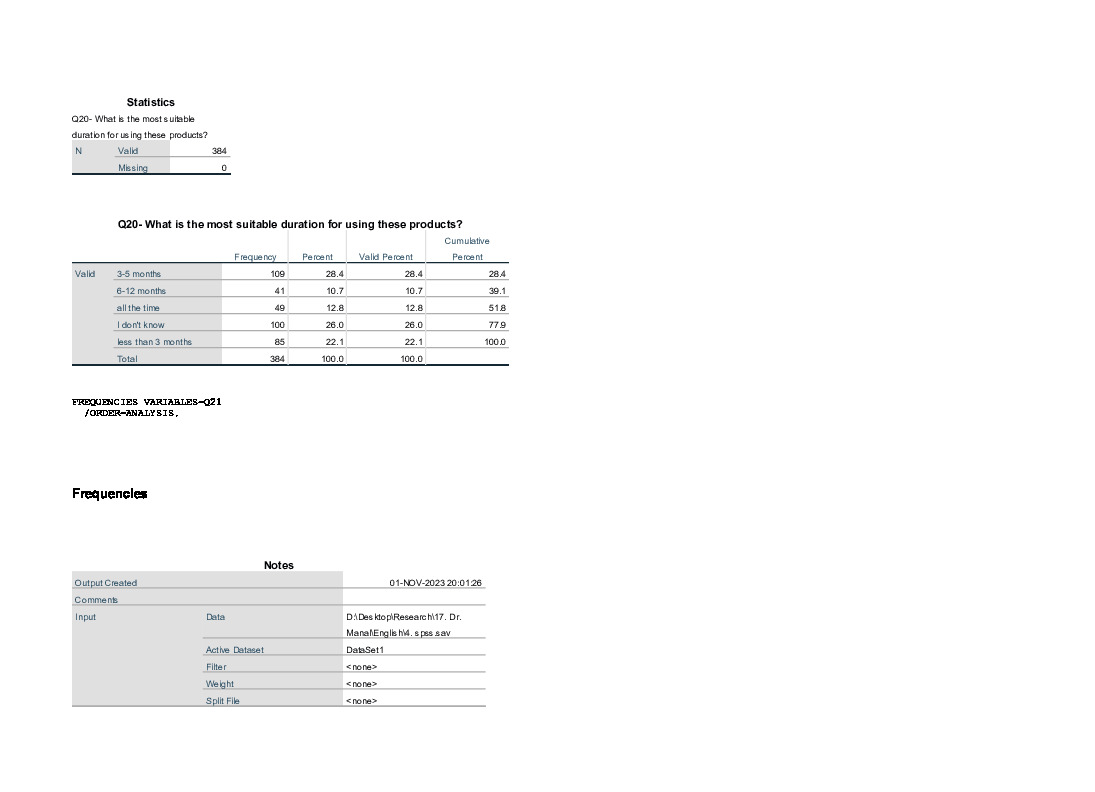

Supplement: S1 Fig — (ZIP) [file pone.0293896.s001.zip › Outputs - word file 32.tiff]

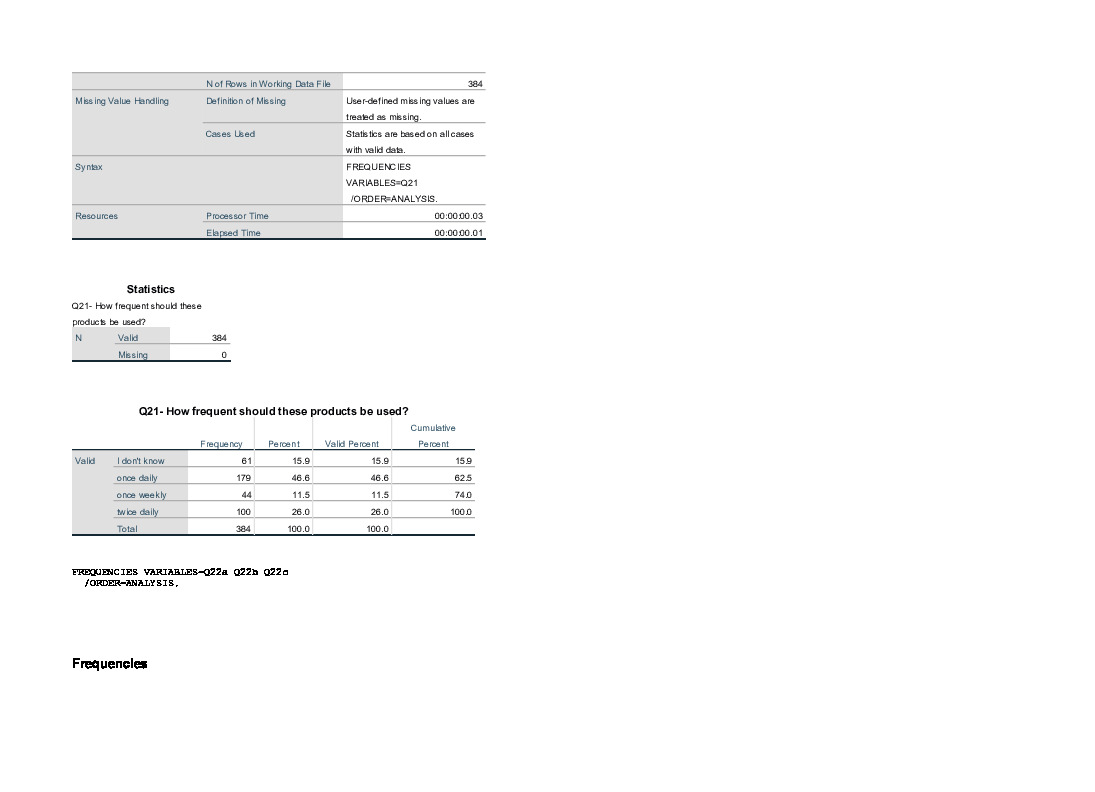

Supplement: S1 Fig — (ZIP) [file pone.0293896.s001.zip › Outputs - word file 33.tiff]

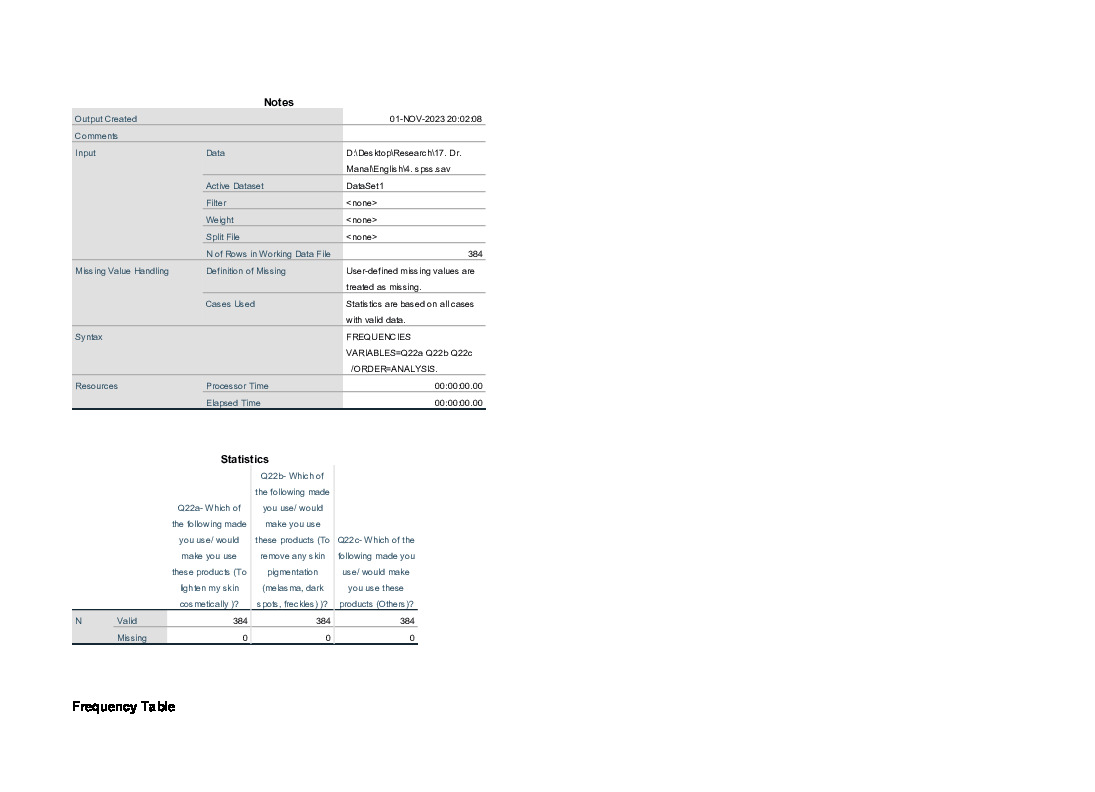

Supplement: S1 Fig — (ZIP) [file pone.0293896.s001.zip › Outputs - word file 34.tiff]

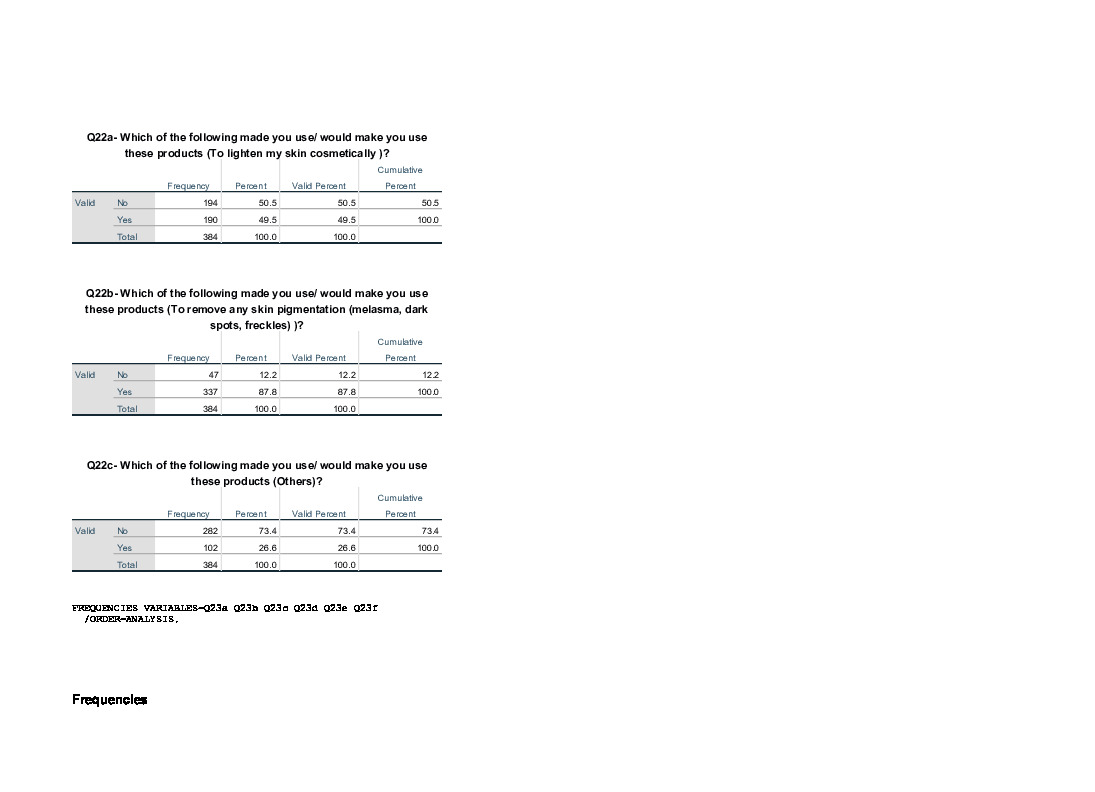

Supplement: S1 Fig — (ZIP) [file pone.0293896.s001.zip › Outputs - word file 35.tiff]

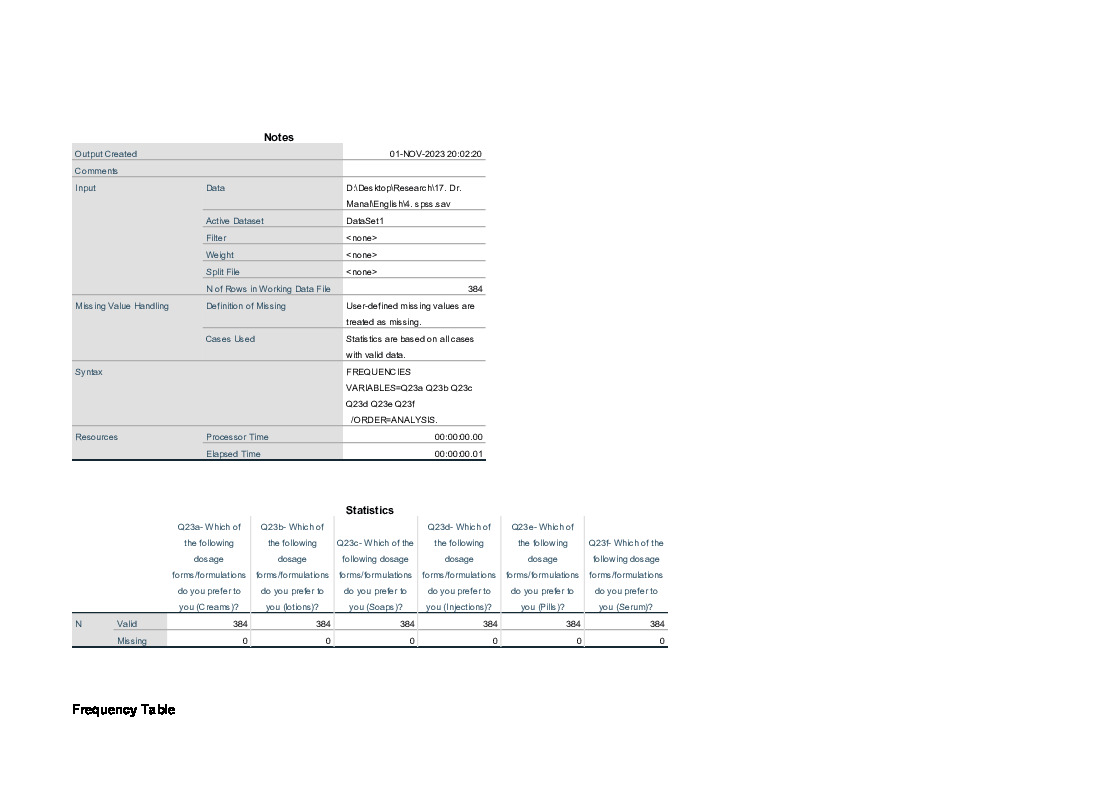

Supplement: S1 Fig — (ZIP) [file pone.0293896.s001.zip › Outputs - word file 36.tiff]

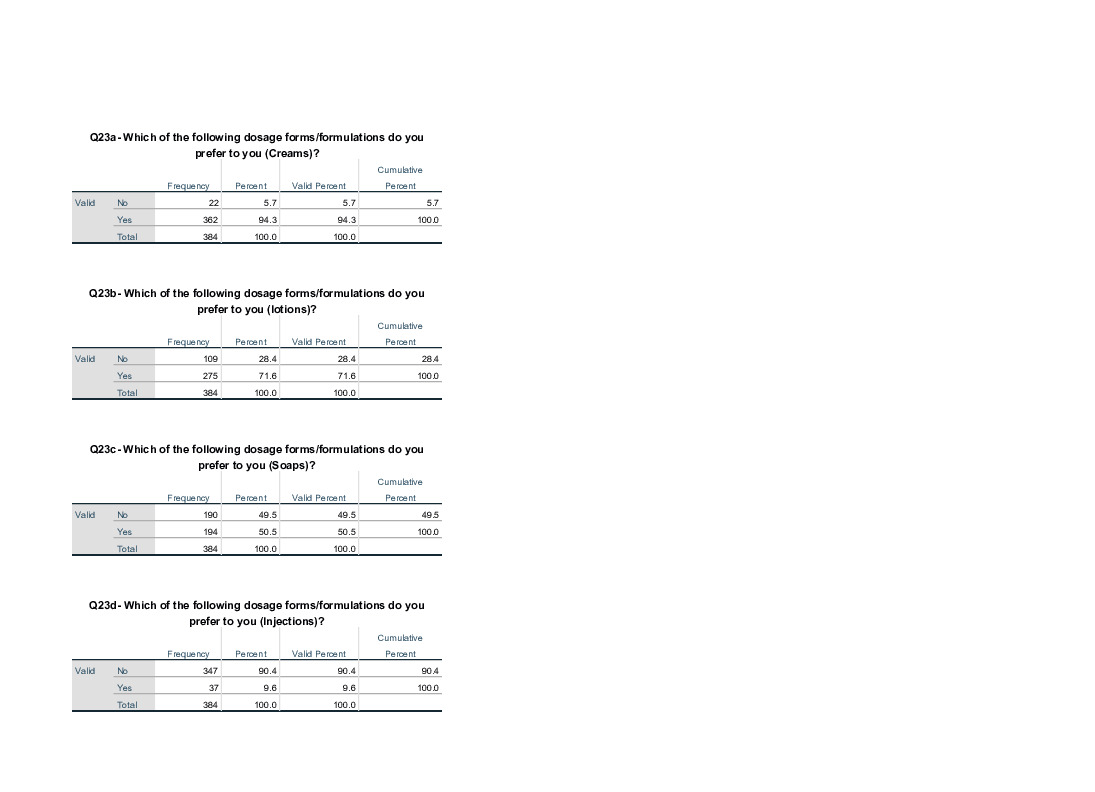

Supplement: S1 Fig — (ZIP) [file pone.0293896.s001.zip › Outputs - word file 37.tiff]

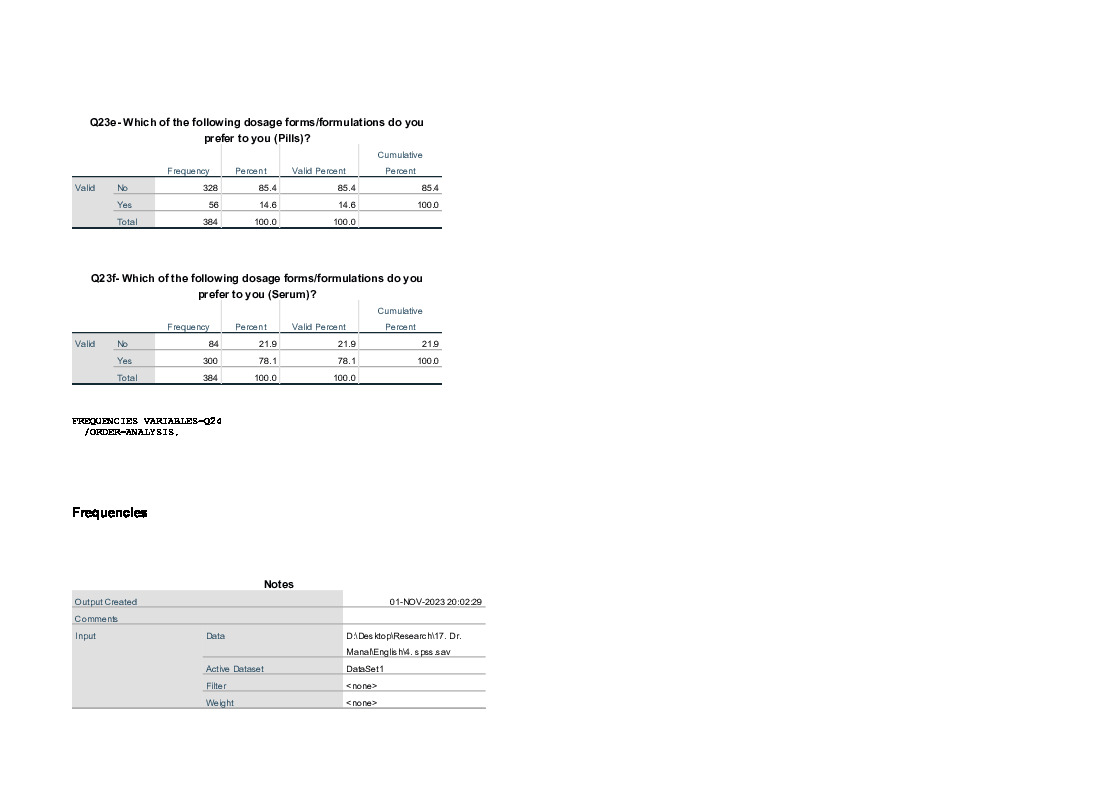

Supplement: S1 Fig — (ZIP) [file pone.0293896.s001.zip › Outputs - word file 38.tiff]

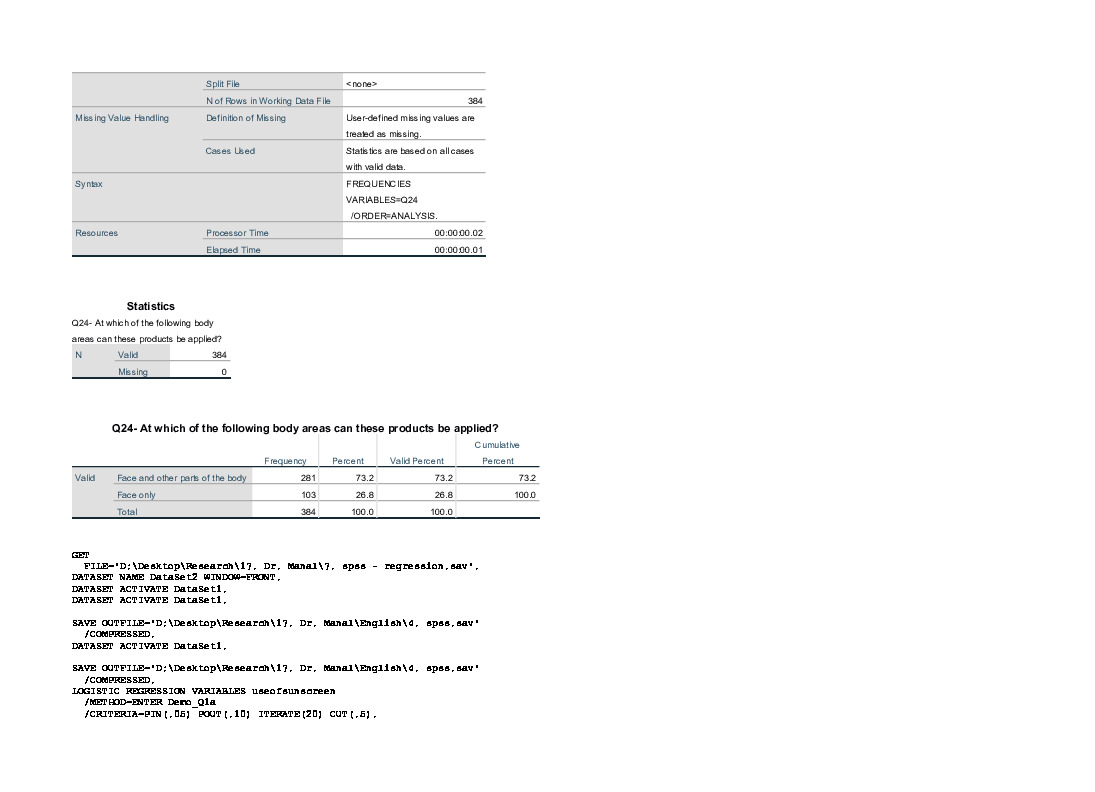

Supplement: S1 Fig — (ZIP) [file pone.0293896.s001.zip › Outputs - word file 39.tiff]

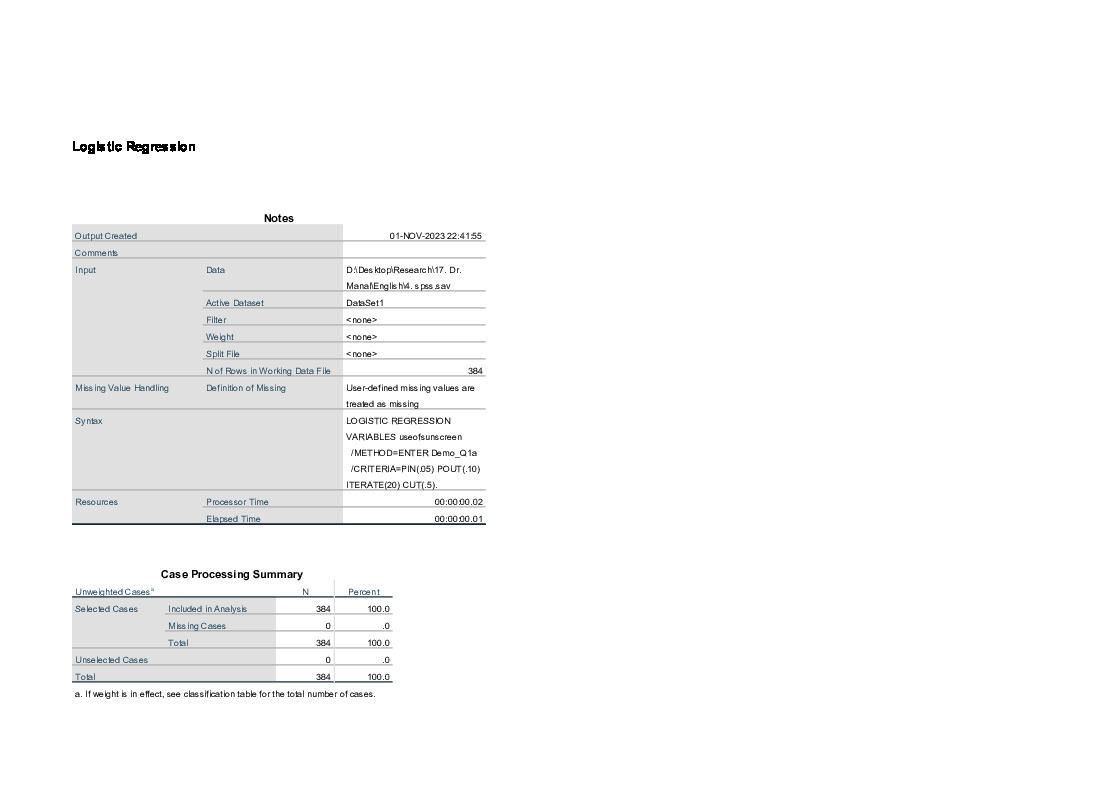

Supplement: S1 Fig — (ZIP) [file pone.0293896.s001.zip › Outputs - word file 40.tiff]

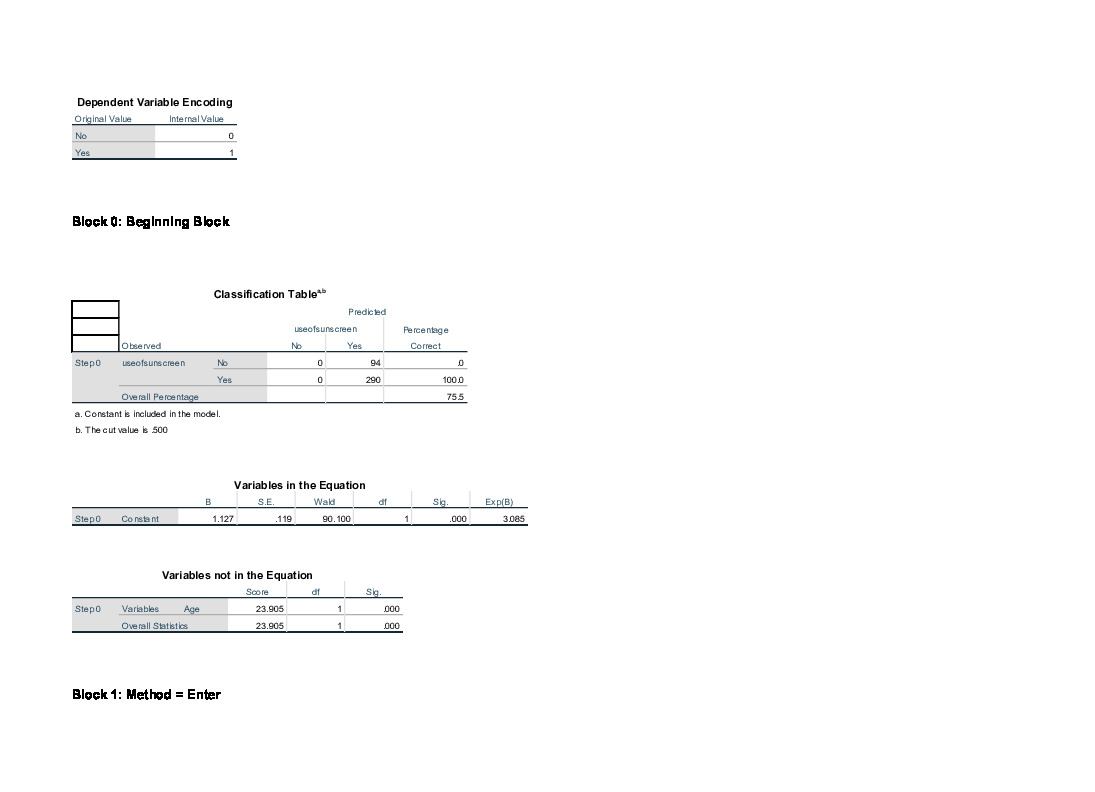

Supplement: S1 Fig — (ZIP) [file pone.0293896.s001.zip › Outputs - word file 41.tiff]

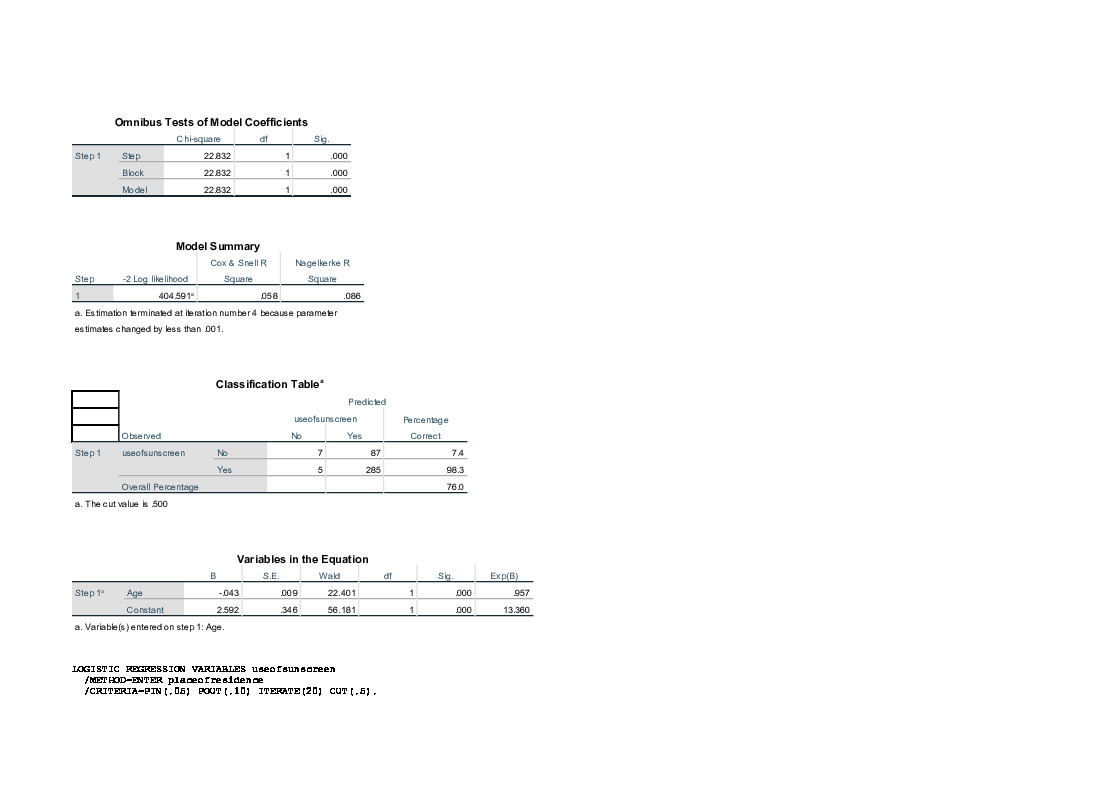

Supplement: S1 Fig — (ZIP) [file pone.0293896.s001.zip › Outputs - word file 42.tiff]

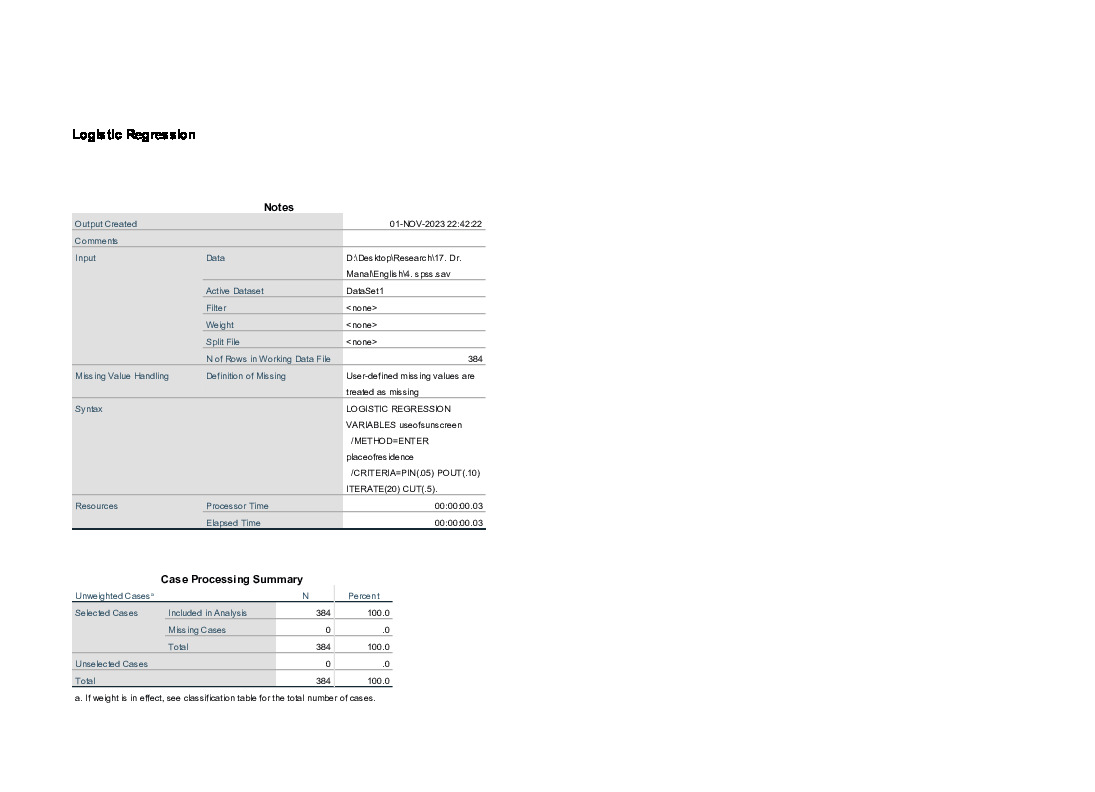

Supplement: S1 Fig — (ZIP) [file pone.0293896.s001.zip › Outputs - word file 43.tiff]

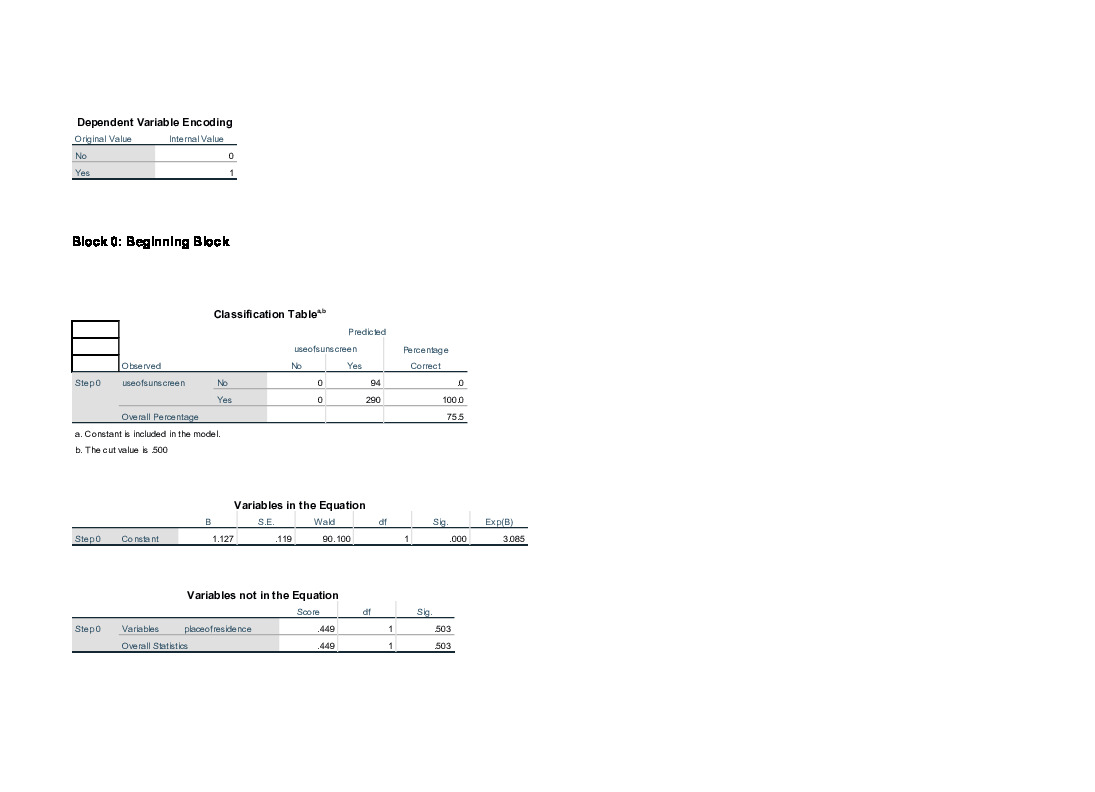

Supplement: S1 Fig — (ZIP) [file pone.0293896.s001.zip › Outputs - word file 44.tiff]

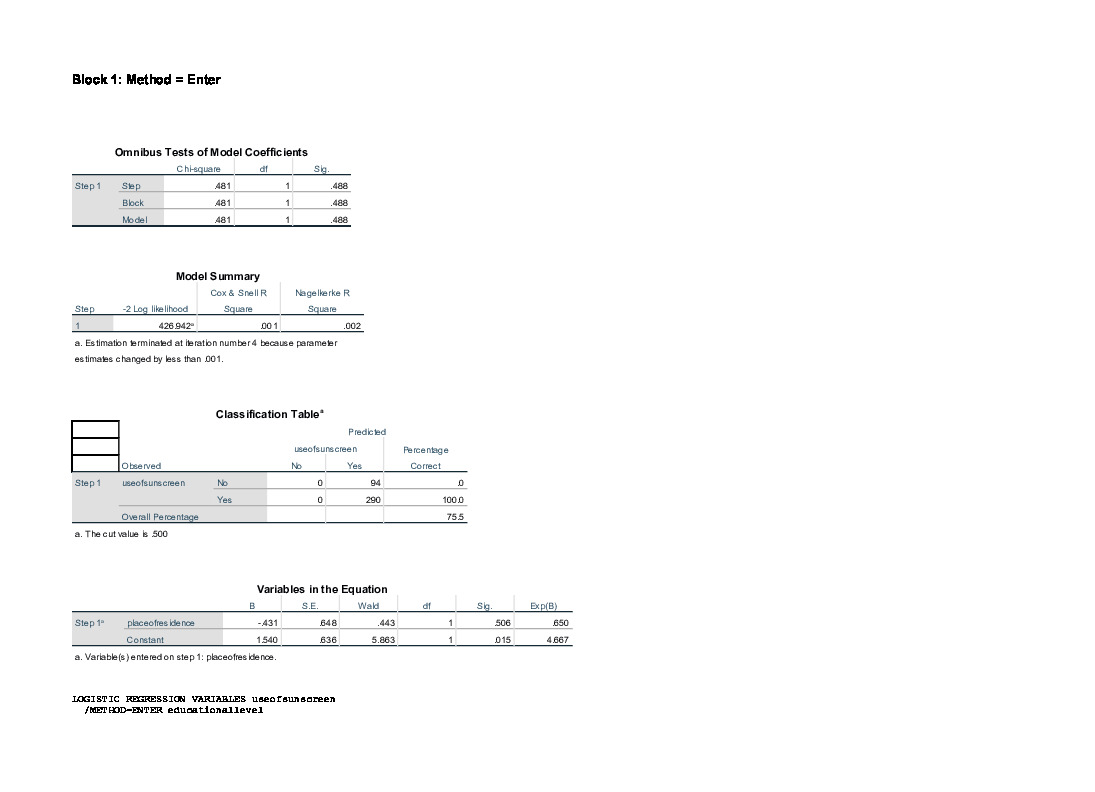

Supplement: S1 Fig — (ZIP) [file pone.0293896.s001.zip › Outputs - word file 45.tiff]

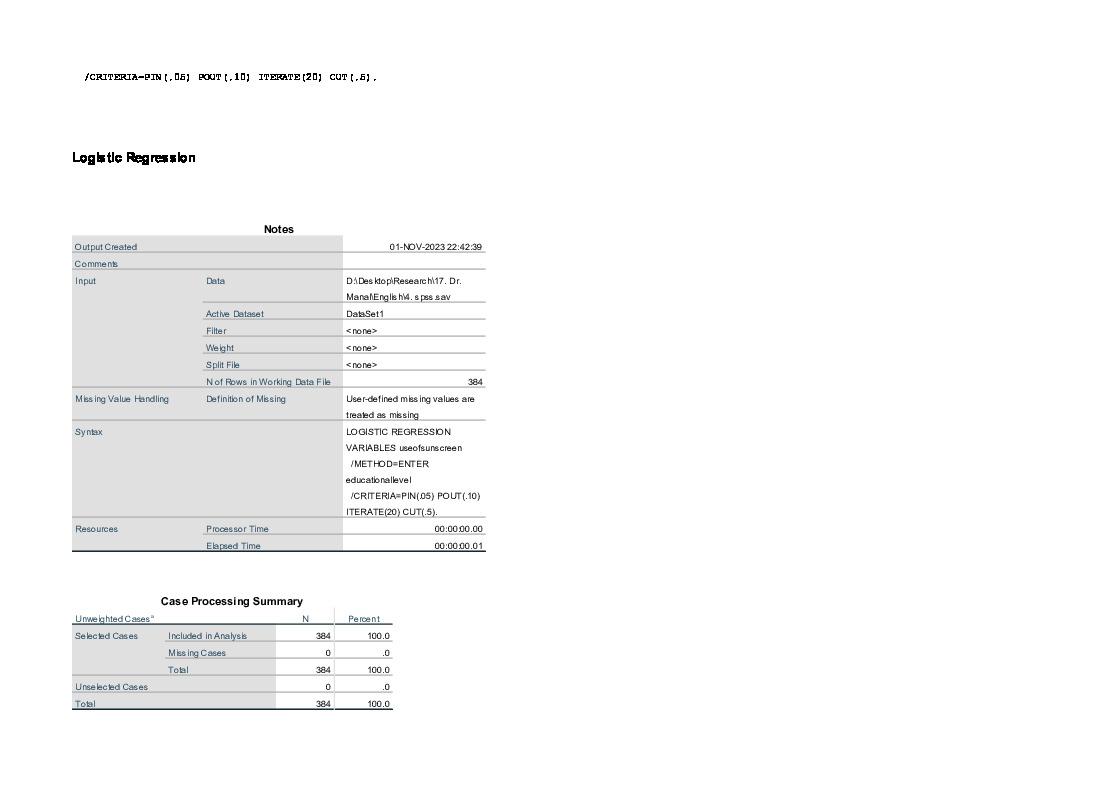

Supplement: S1 Fig — (ZIP) [file pone.0293896.s001.zip › Outputs - word file 46.tiff]

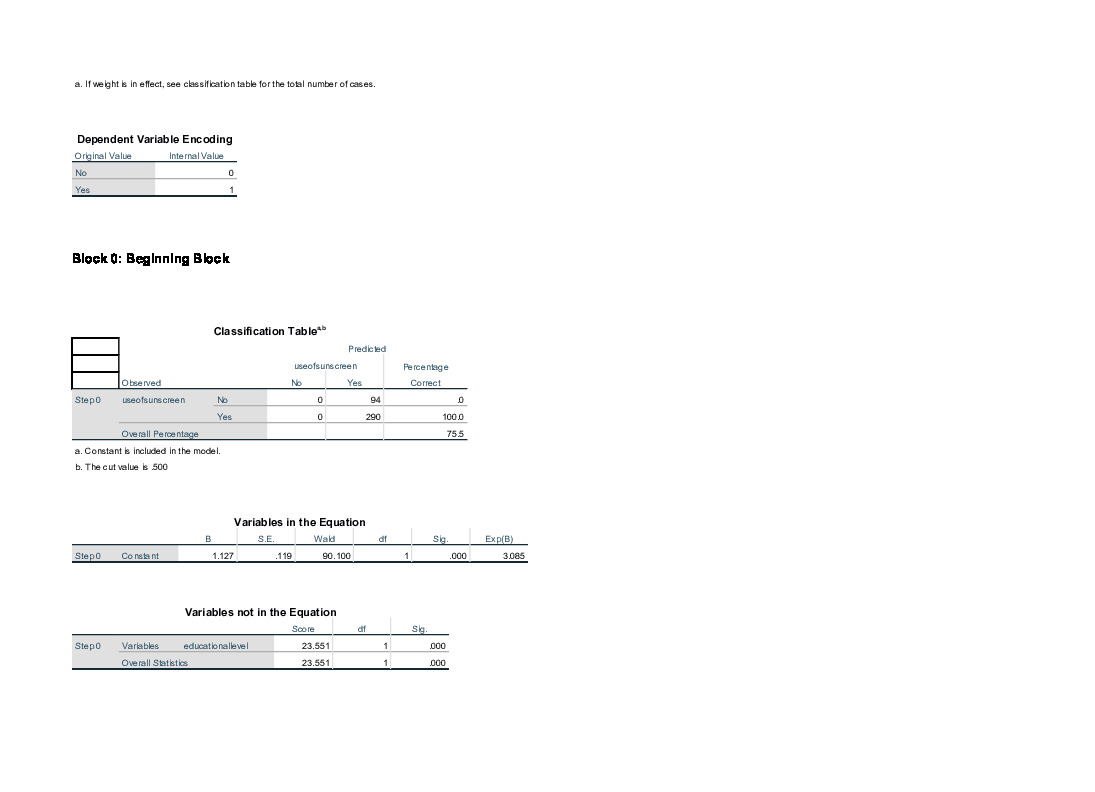

Supplement: S1 Fig — (ZIP) [file pone.0293896.s001.zip › Outputs - word file 47.tiff]

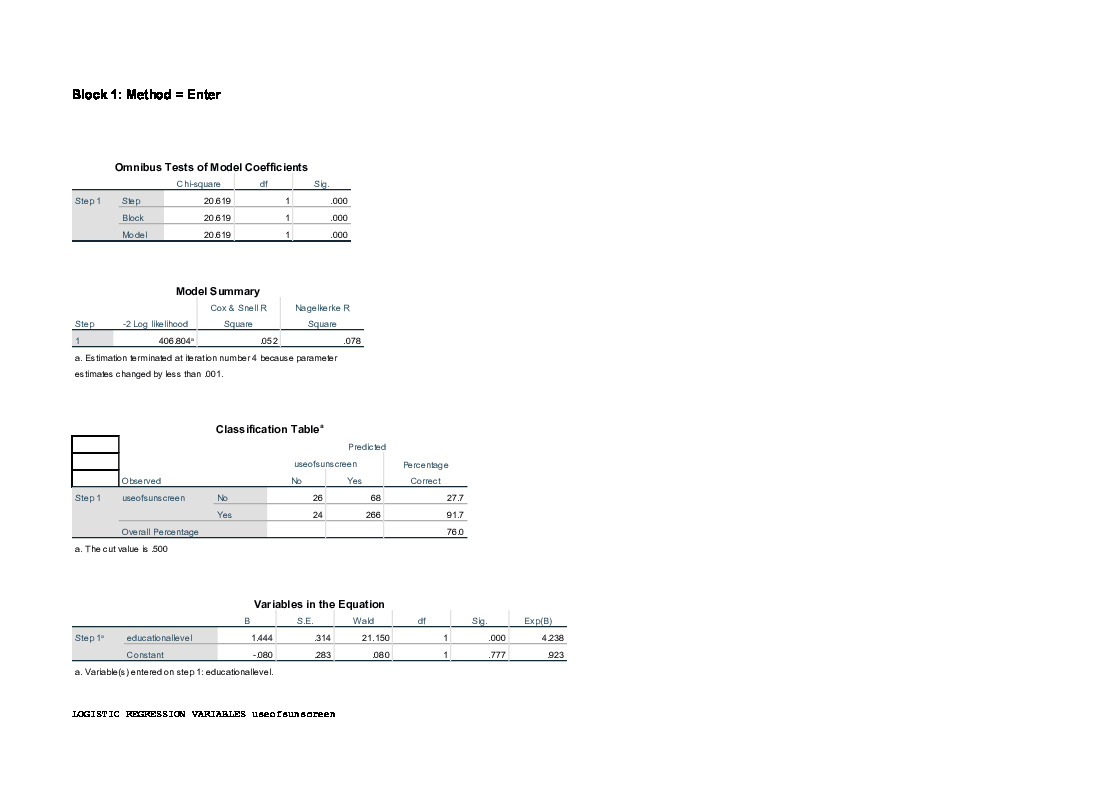

Supplement: S1 Fig — (ZIP) [file pone.0293896.s001.zip › Outputs - word file 48.tiff]

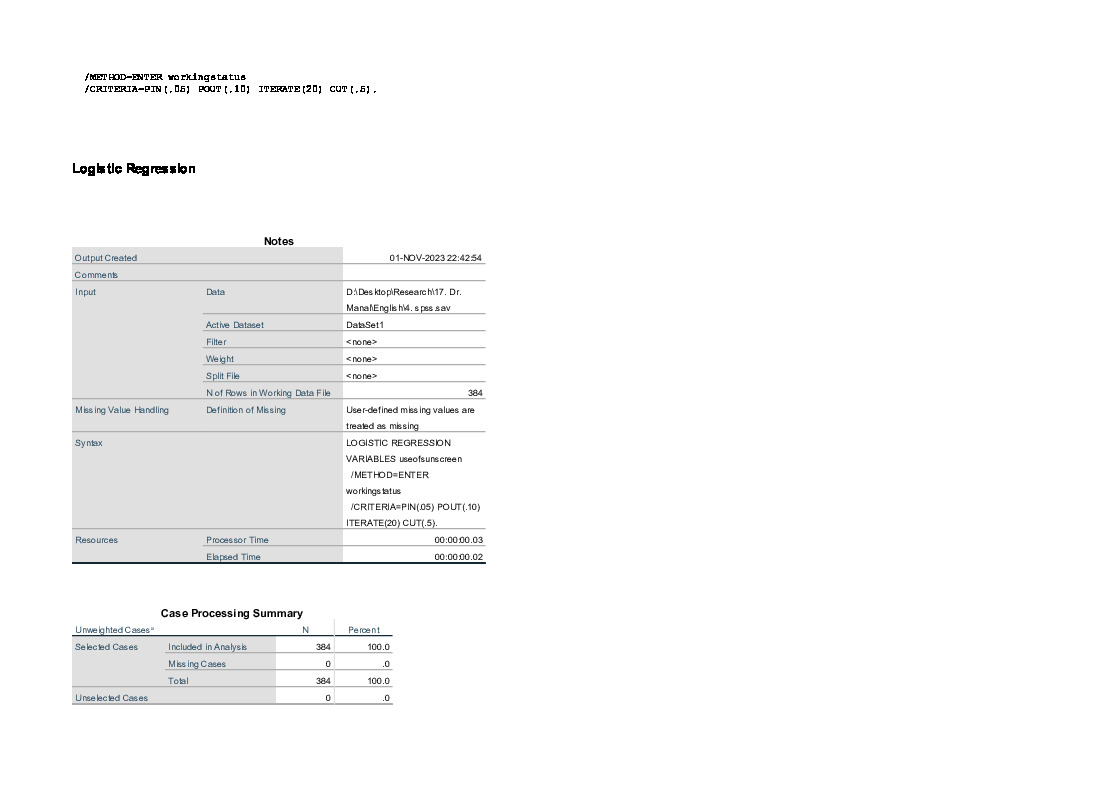

Supplement: S1 Fig — (ZIP) [file pone.0293896.s001.zip › Outputs - word file 49.tiff]

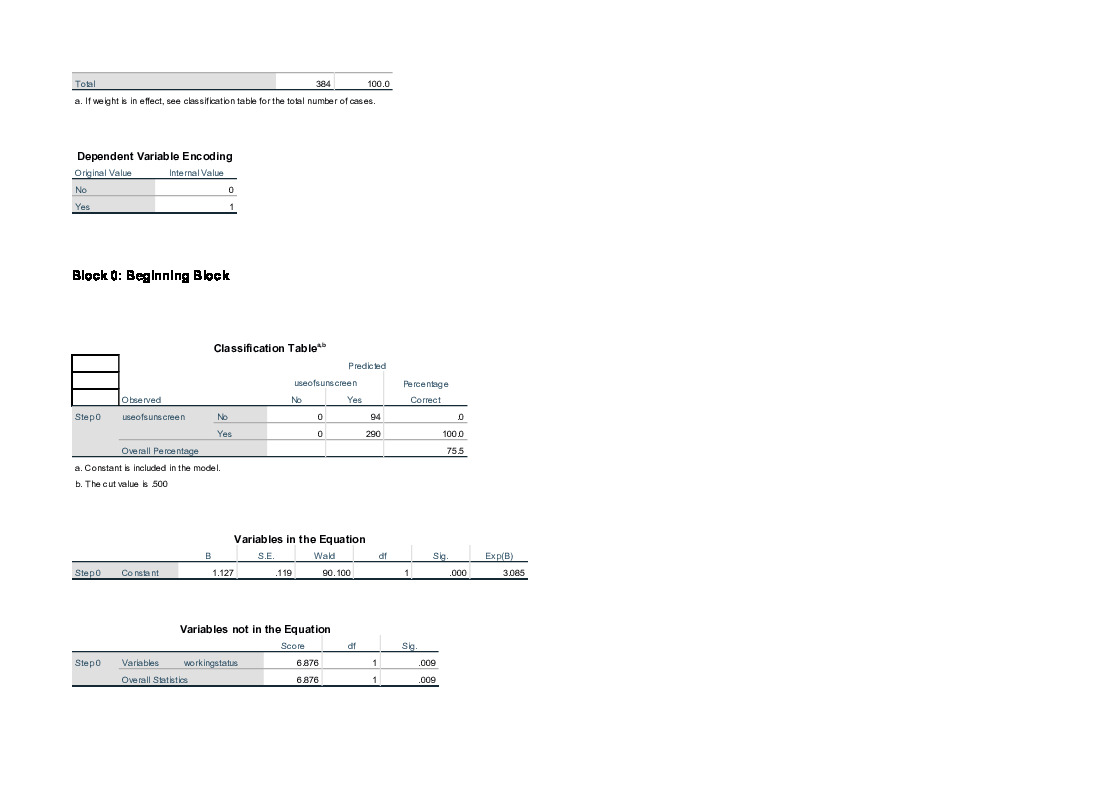

Supplement: S1 Fig — (ZIP) [file pone.0293896.s001.zip › Outputs - word file 50.tiff]

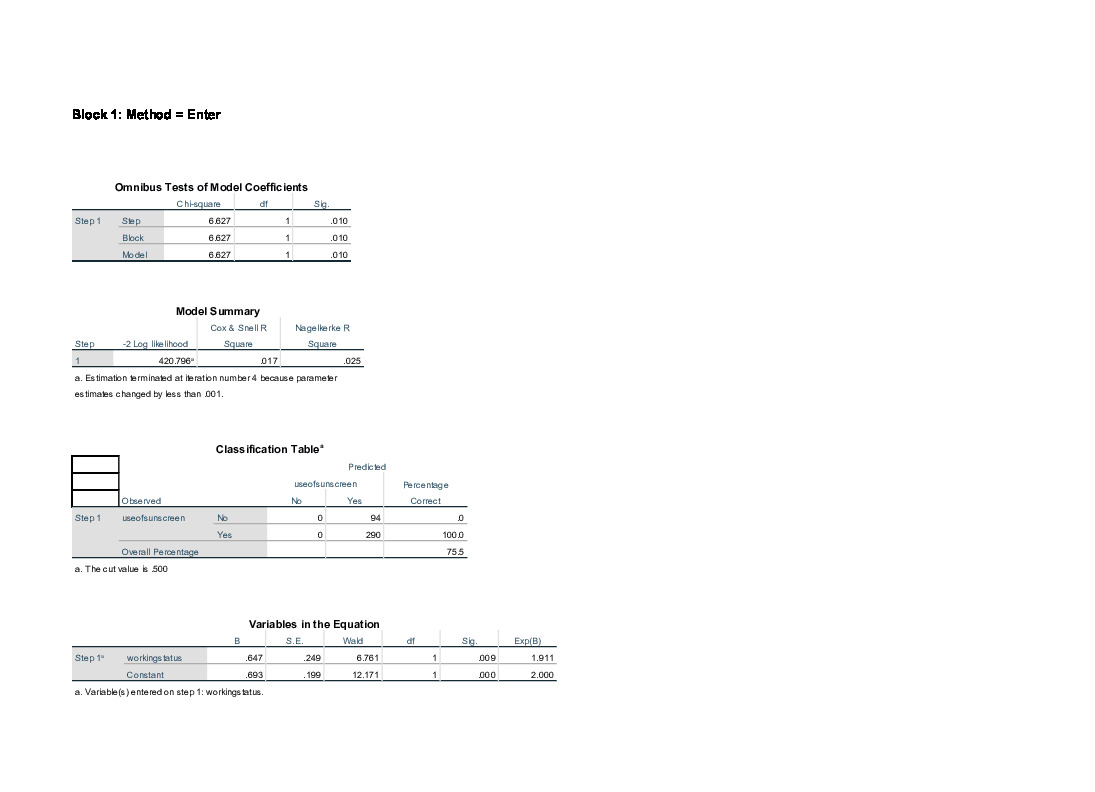

Supplement: S1 Fig — (ZIP) [file pone.0293896.s001.zip › Outputs - word file 51.tiff]

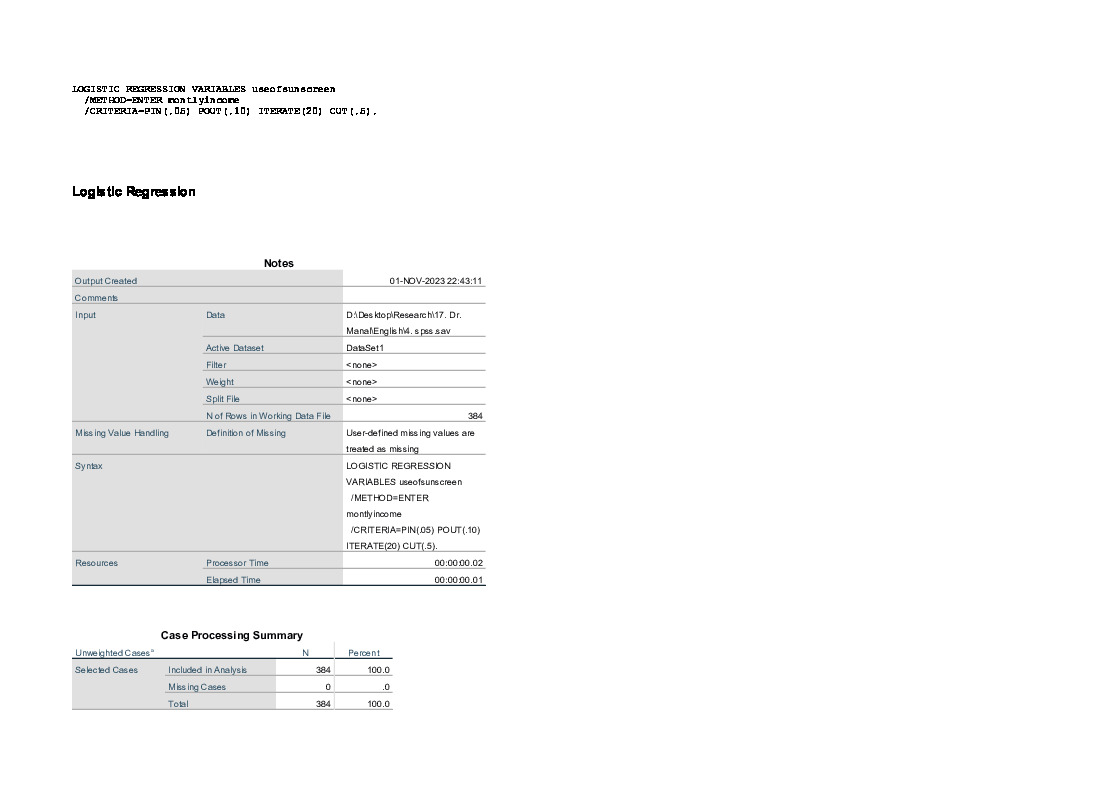

Supplement: S1 Fig — (ZIP) [file pone.0293896.s001.zip › Outputs - word file 52.tiff]

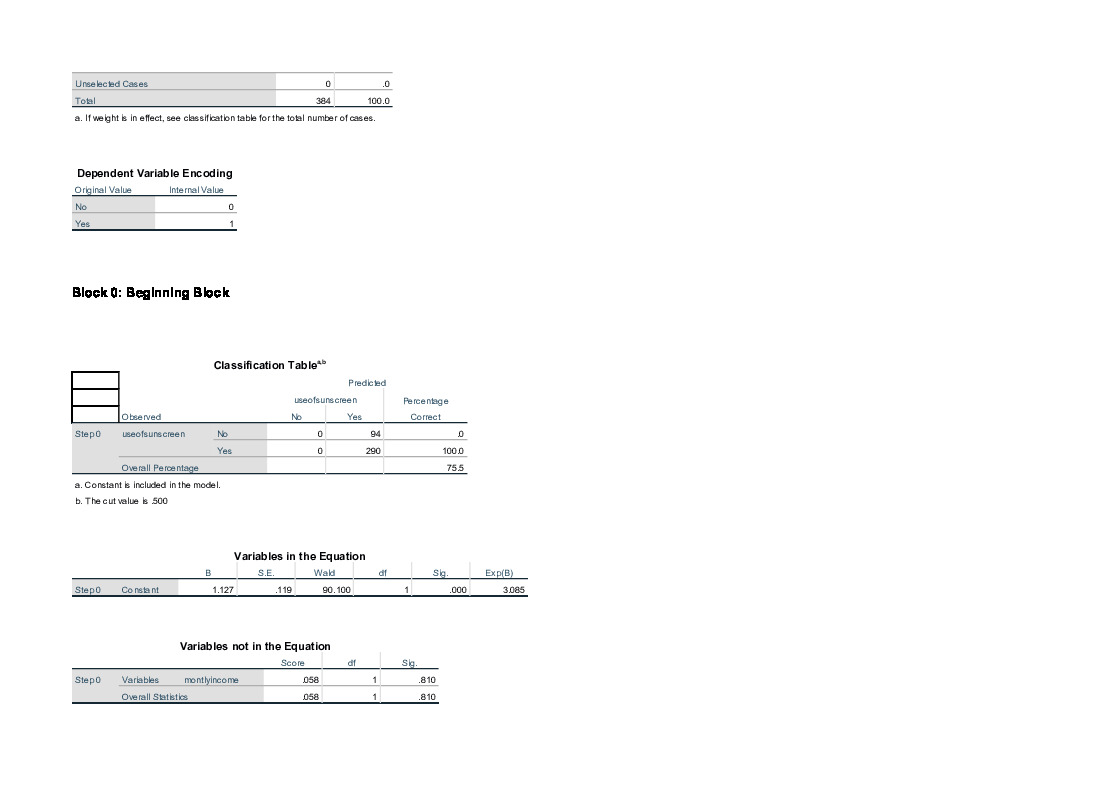

Supplement: S1 Fig — (ZIP) [file pone.0293896.s001.zip › Outputs - word file 53.tiff]

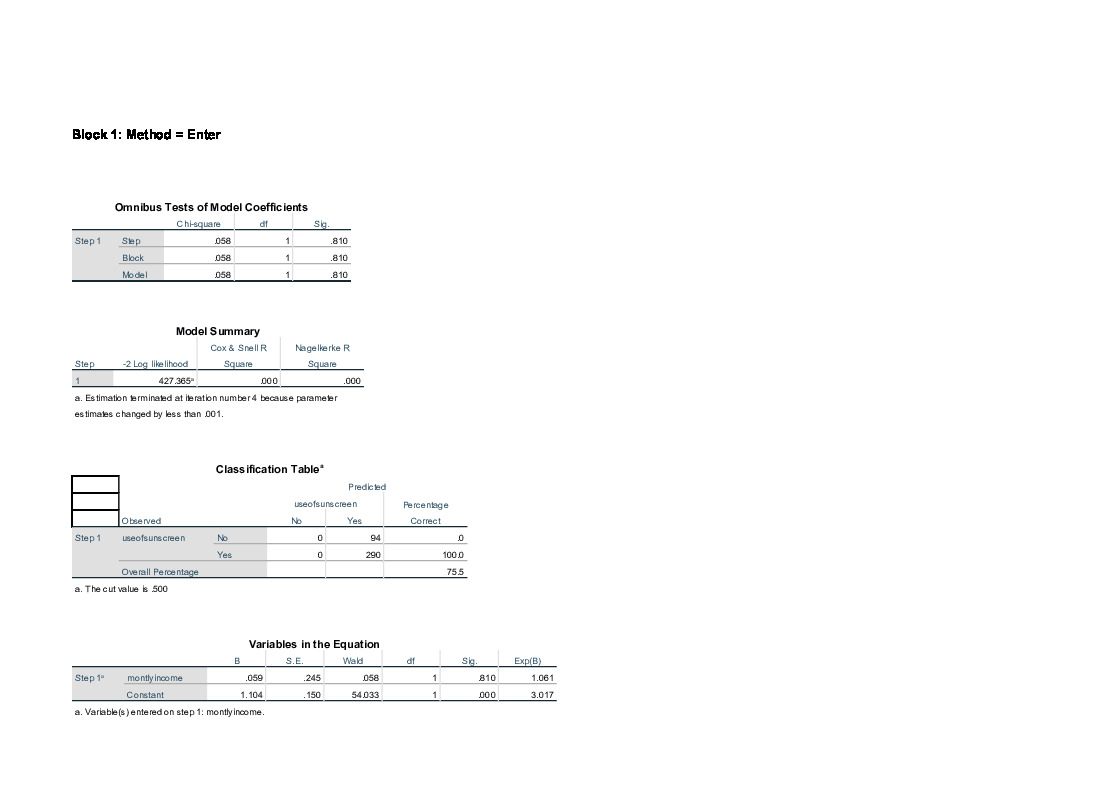

Supplement: S1 Fig — (ZIP) [file pone.0293896.s001.zip › Outputs - word file 54.tiff]

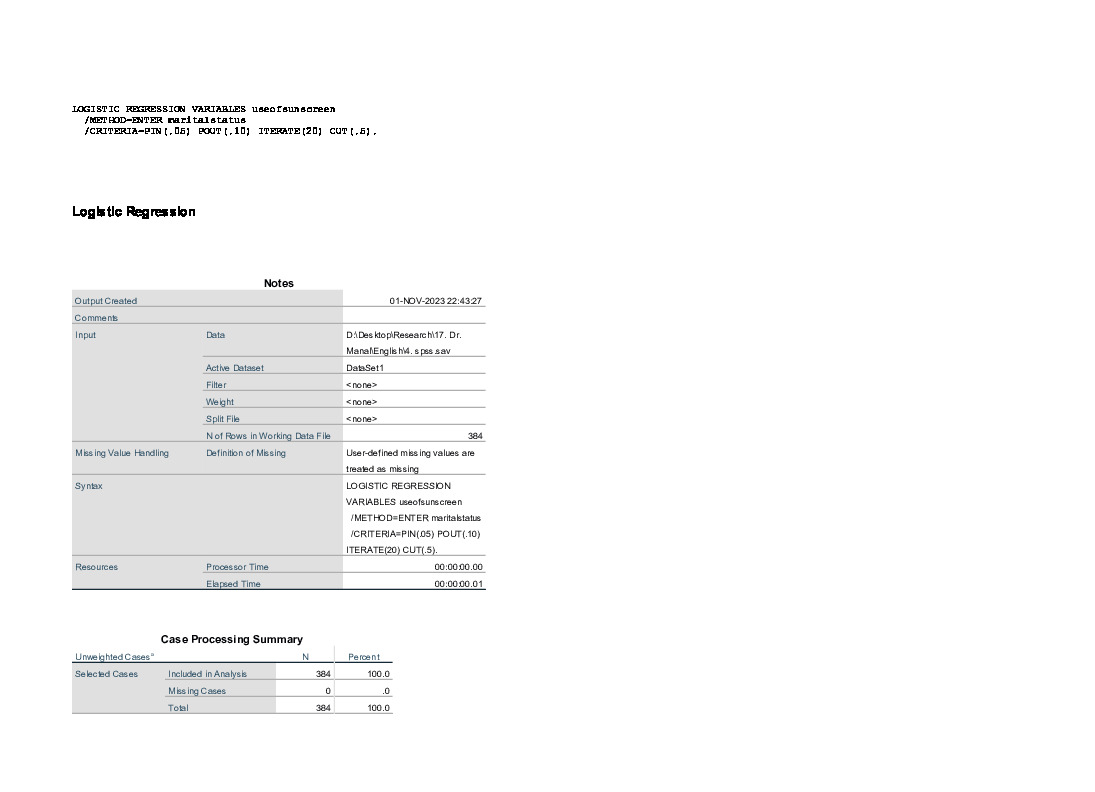

Supplement: S1 Fig — (ZIP) [file pone.0293896.s001.zip › Outputs - word file 55.tiff]

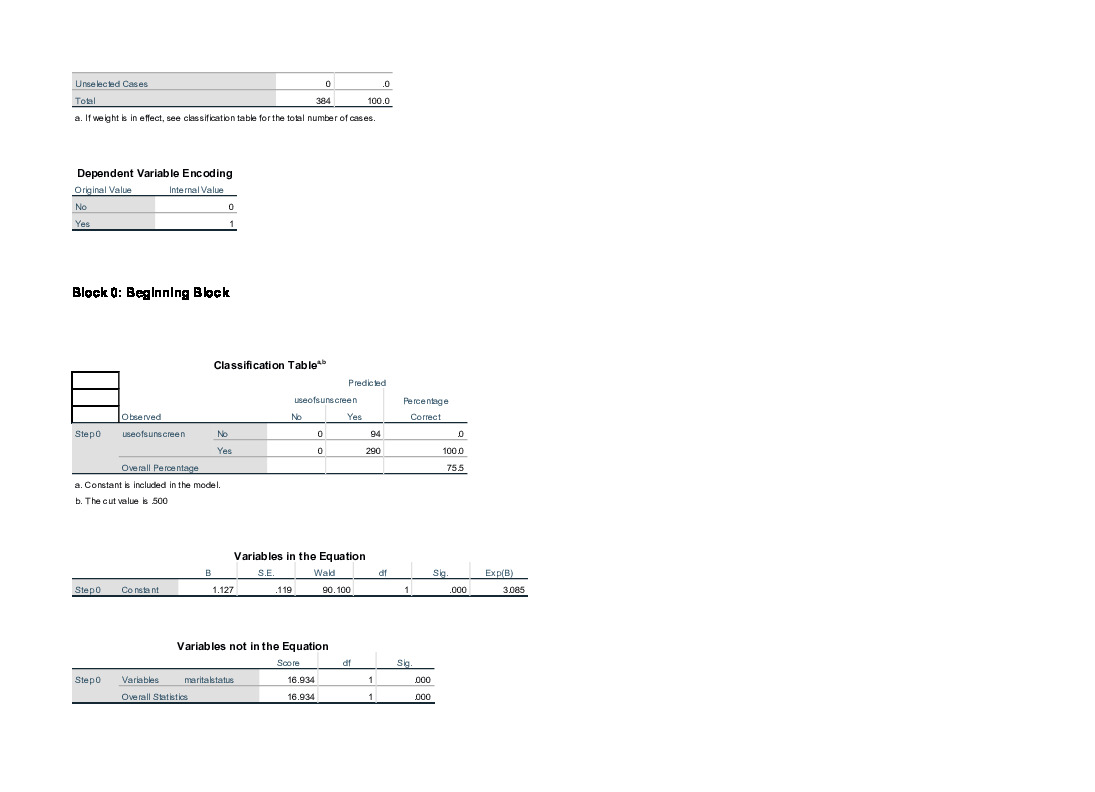

Supplement: S1 Fig — (ZIP) [file pone.0293896.s001.zip › Outputs - word file 56.tiff]

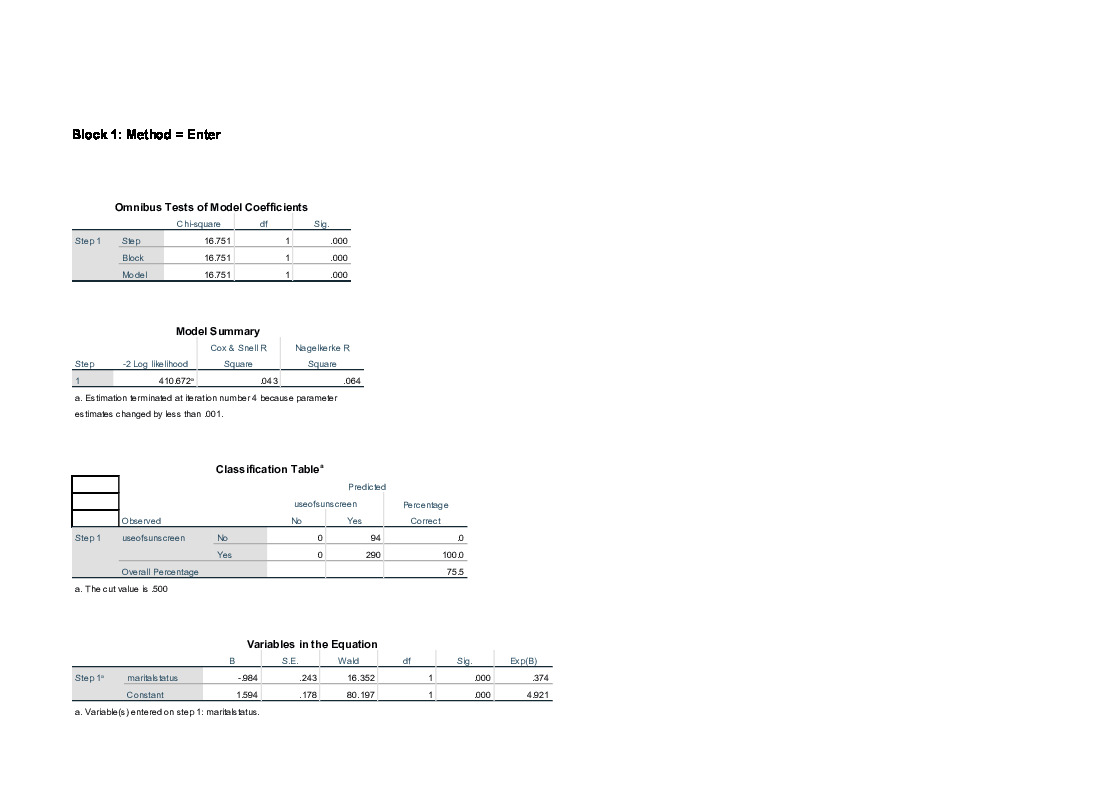

Supplement: S1 Fig — (ZIP) [file pone.0293896.s001.zip › Outputs - word file 57.tiff]

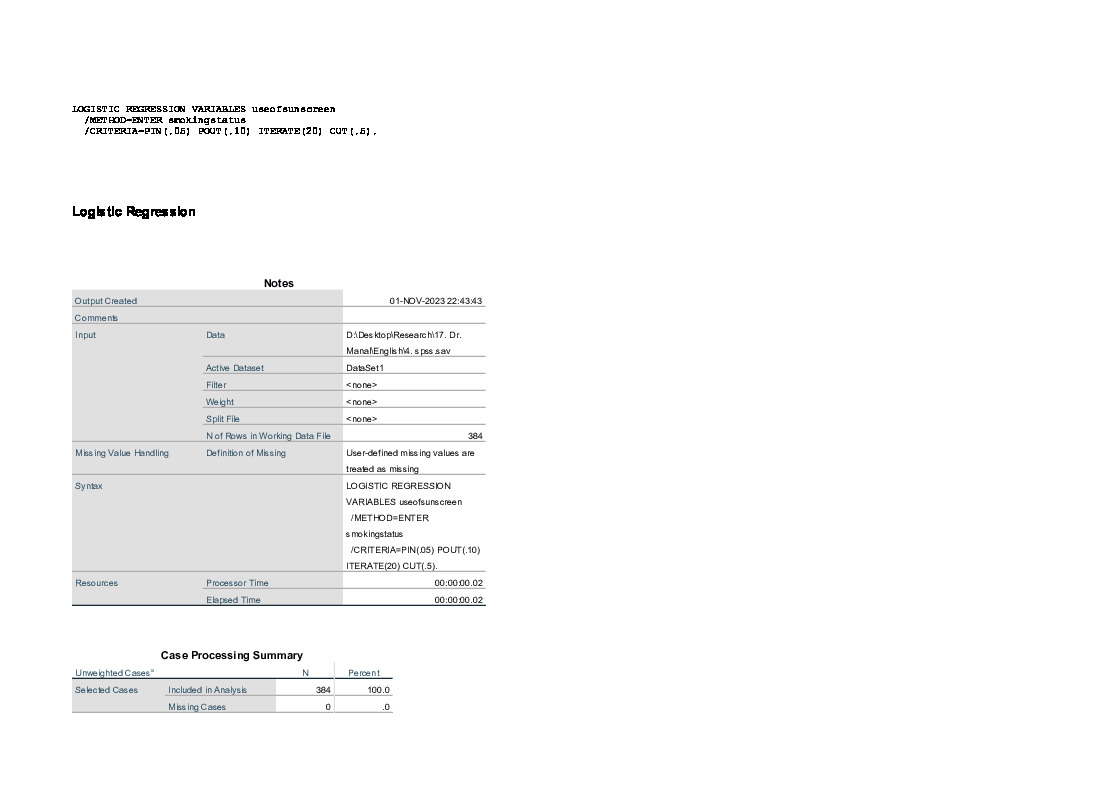

Supplement: S1 Fig — (ZIP) [file pone.0293896.s001.zip › Outputs - word file 58.tiff]

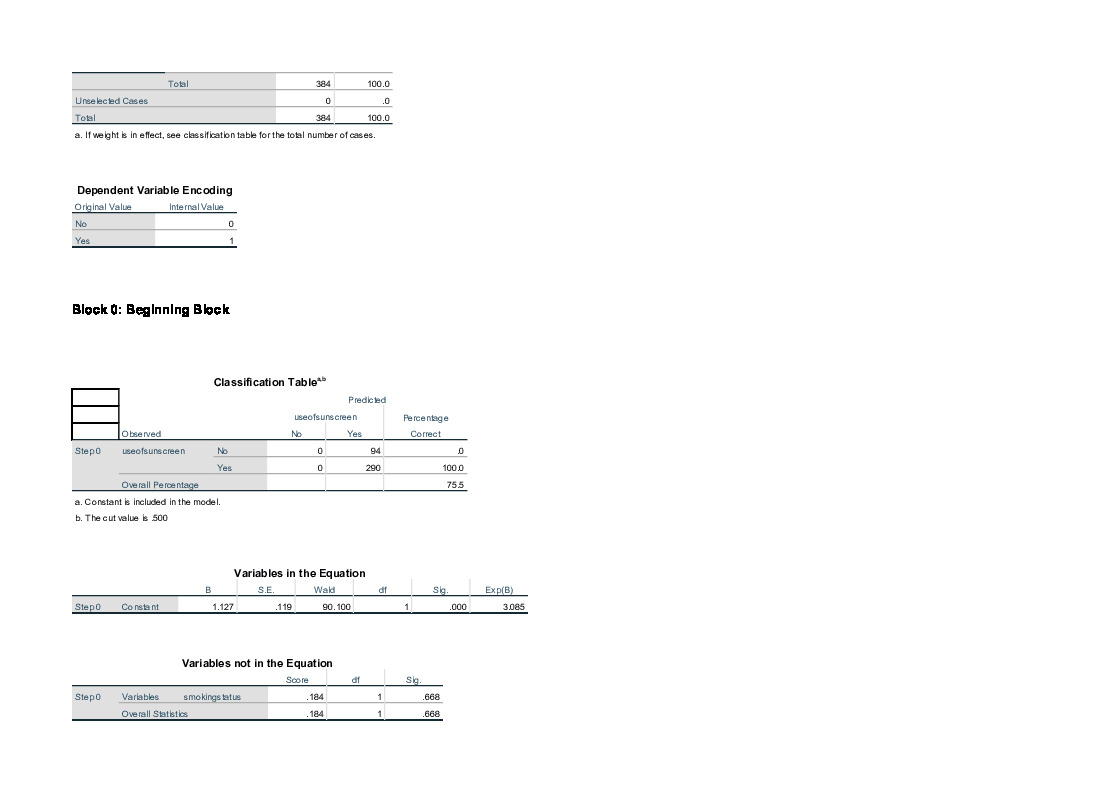

Supplement: S1 Fig — (ZIP) [file pone.0293896.s001.zip › Outputs - word file 59.tiff]

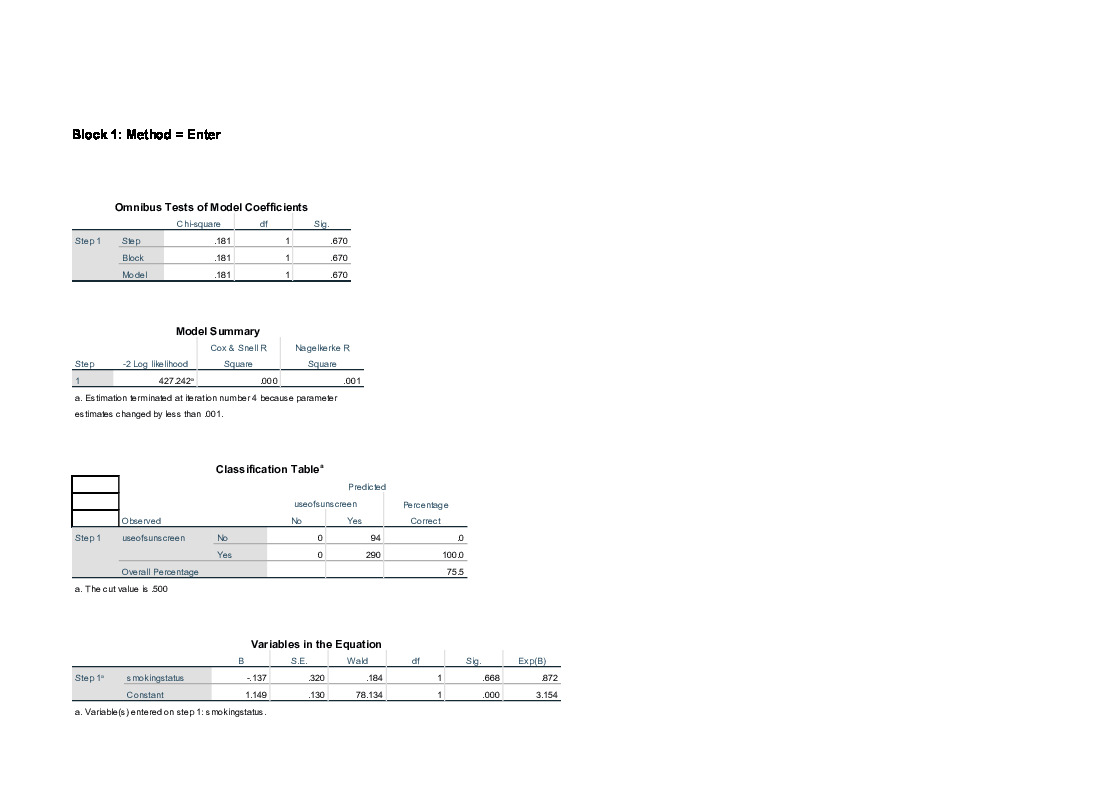

Supplement: S1 Fig — (ZIP) [file pone.0293896.s001.zip › Outputs - word file 60.tiff]

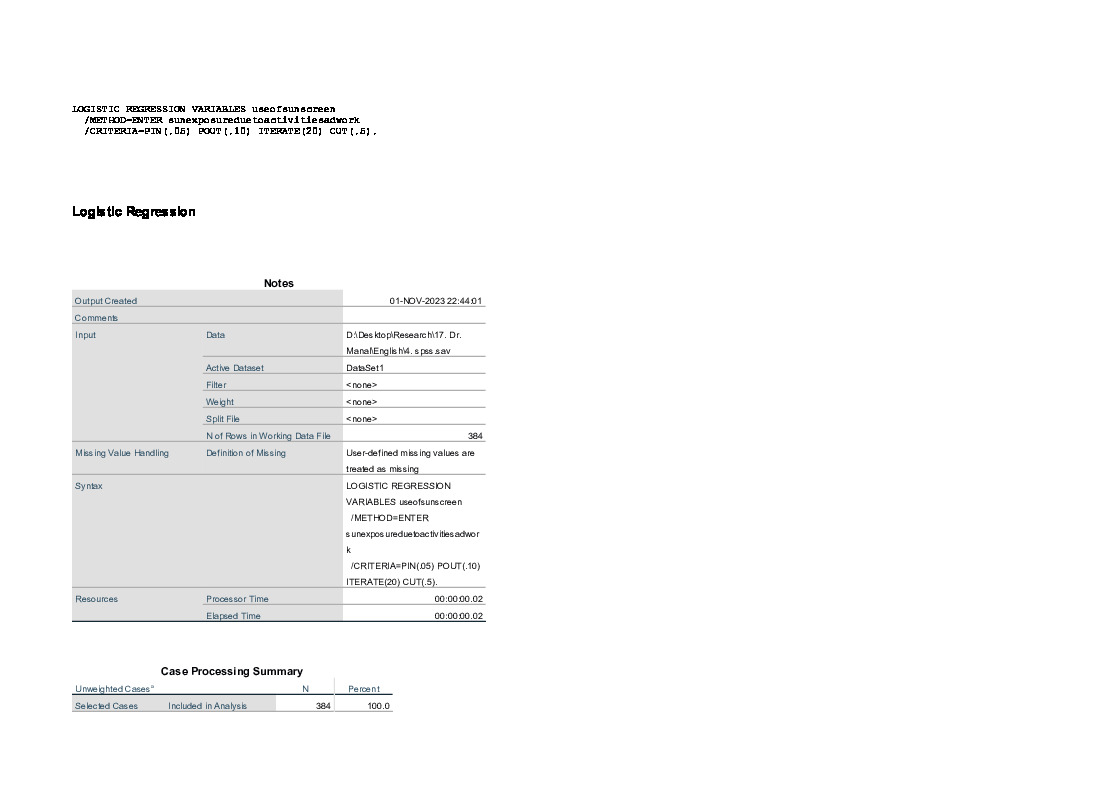

Supplement: S1 Fig — (ZIP) [file pone.0293896.s001.zip › Outputs - word file 61.tiff]

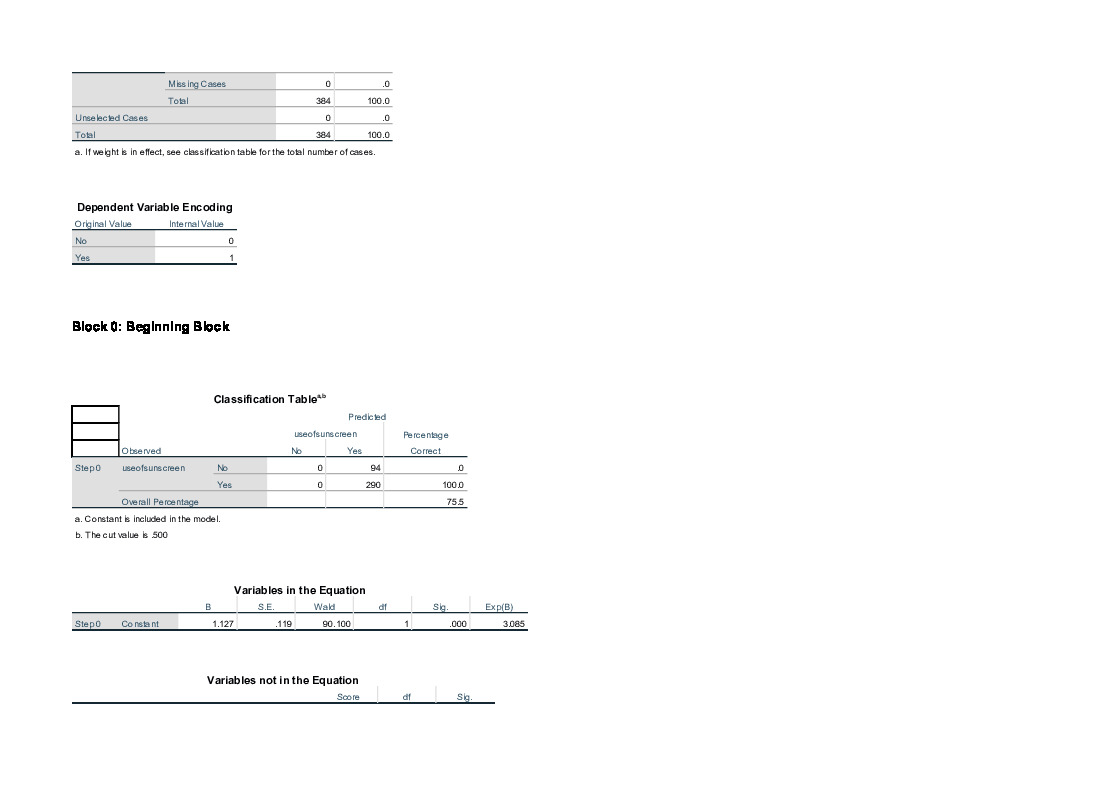

Supplement: S1 Fig — (ZIP) [file pone.0293896.s001.zip › Outputs - word file 62.tiff]

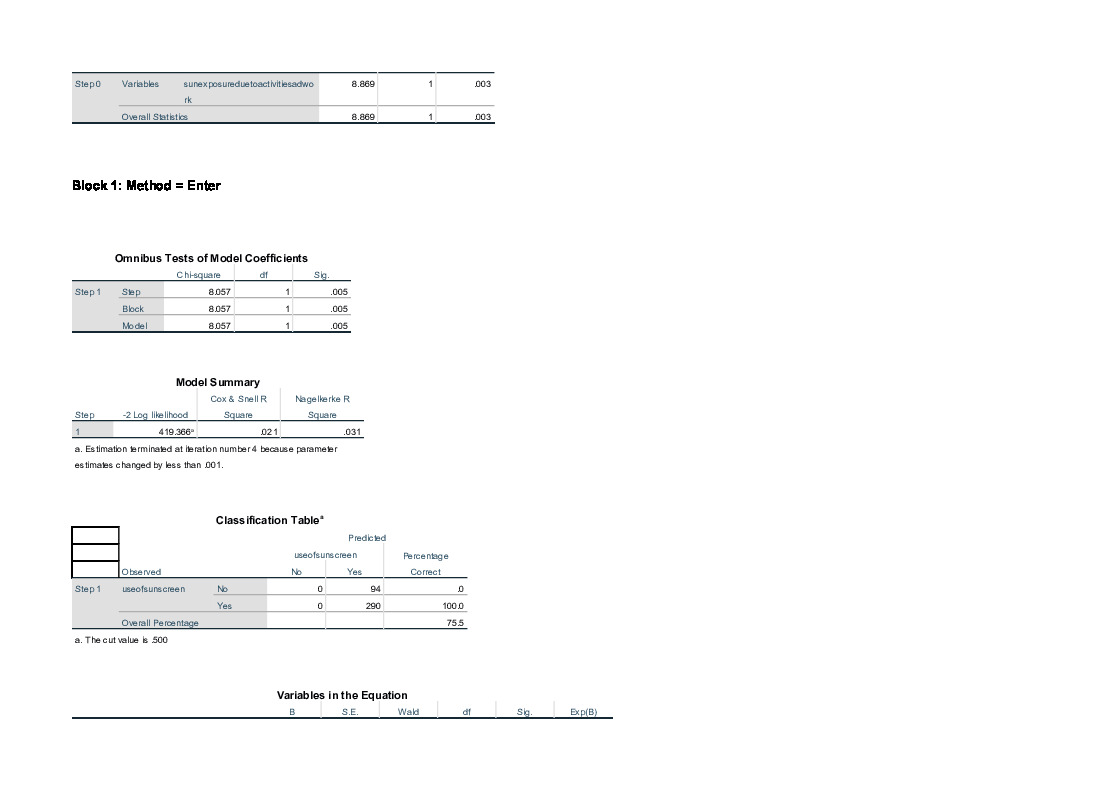

Supplement: S1 Fig — (ZIP) [file pone.0293896.s001.zip › Outputs - word file 63.tiff]

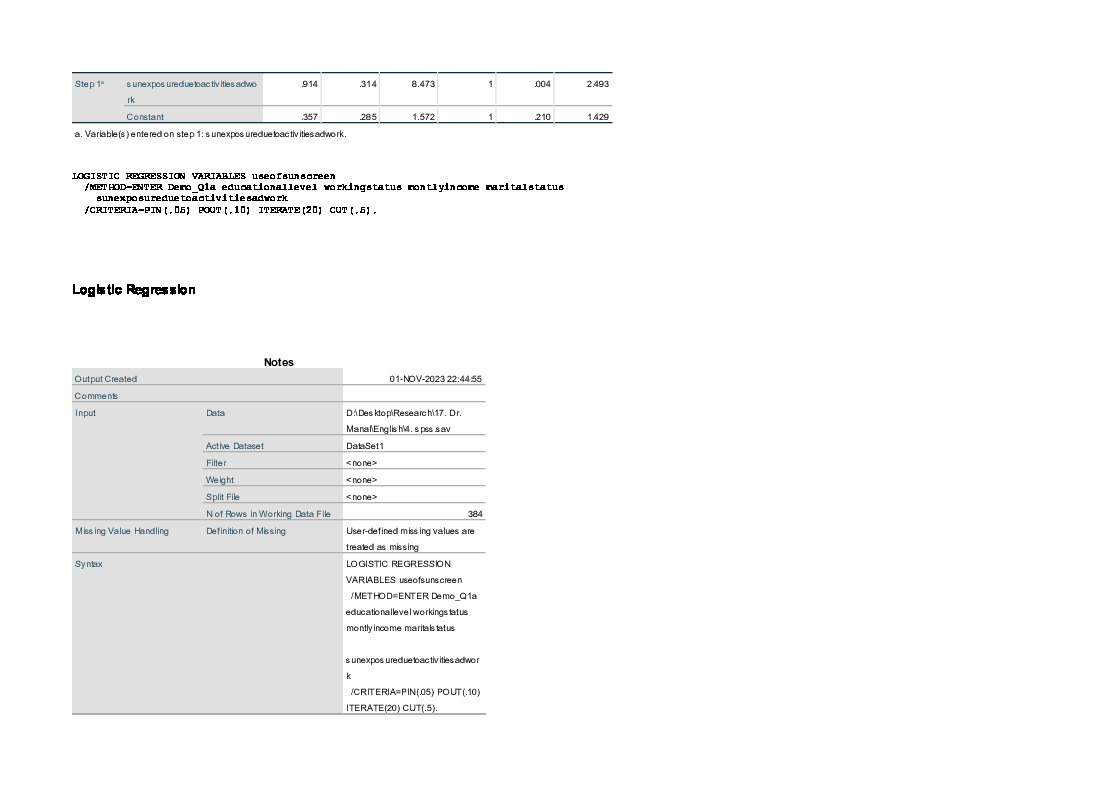

Supplement: S1 Fig — (ZIP) [file pone.0293896.s001.zip › Outputs - word file 64.tiff]

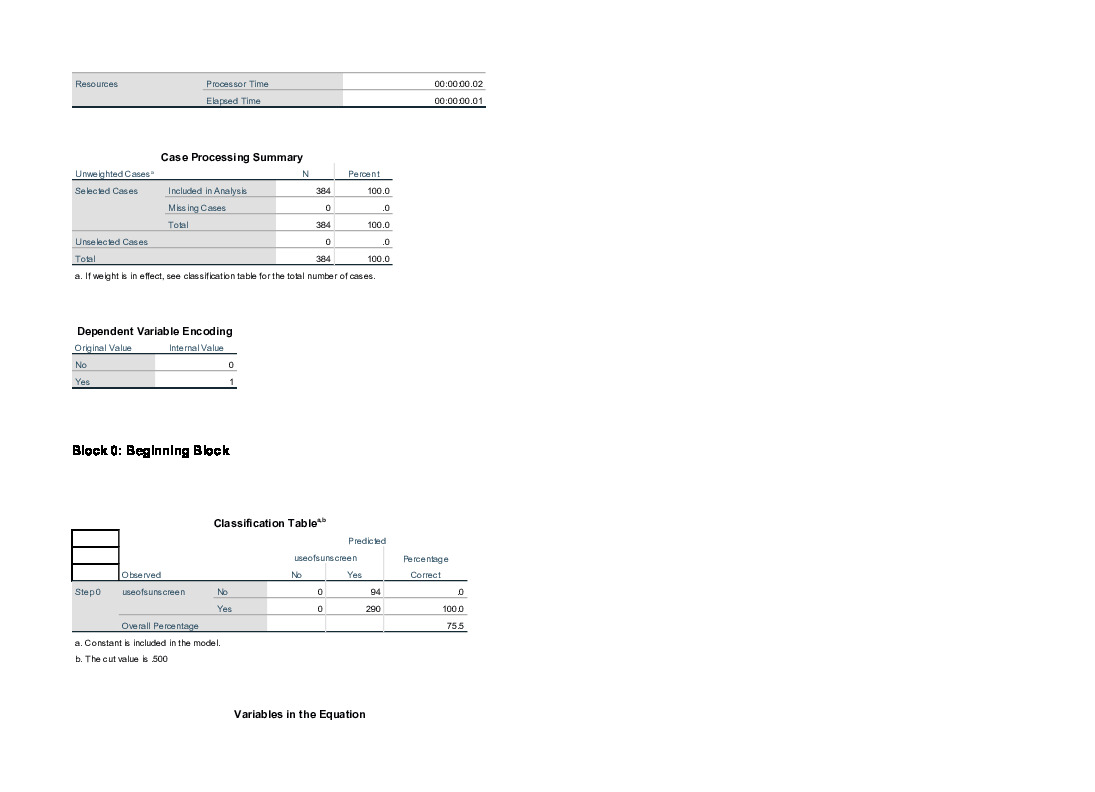

Supplement: S1 Fig — (ZIP) [file pone.0293896.s001.zip › Outputs - word file 65.tiff]

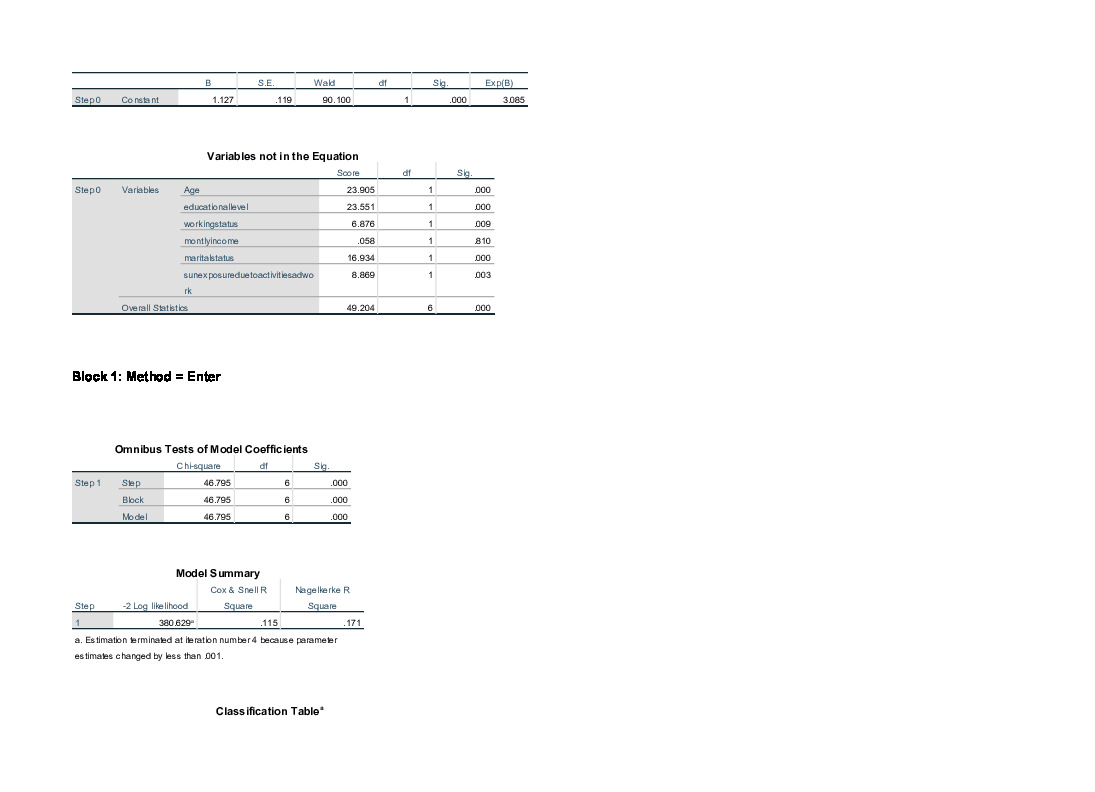

Supplement: S1 Fig — (ZIP) [file pone.0293896.s001.zip › Outputs - word file 66.tiff]

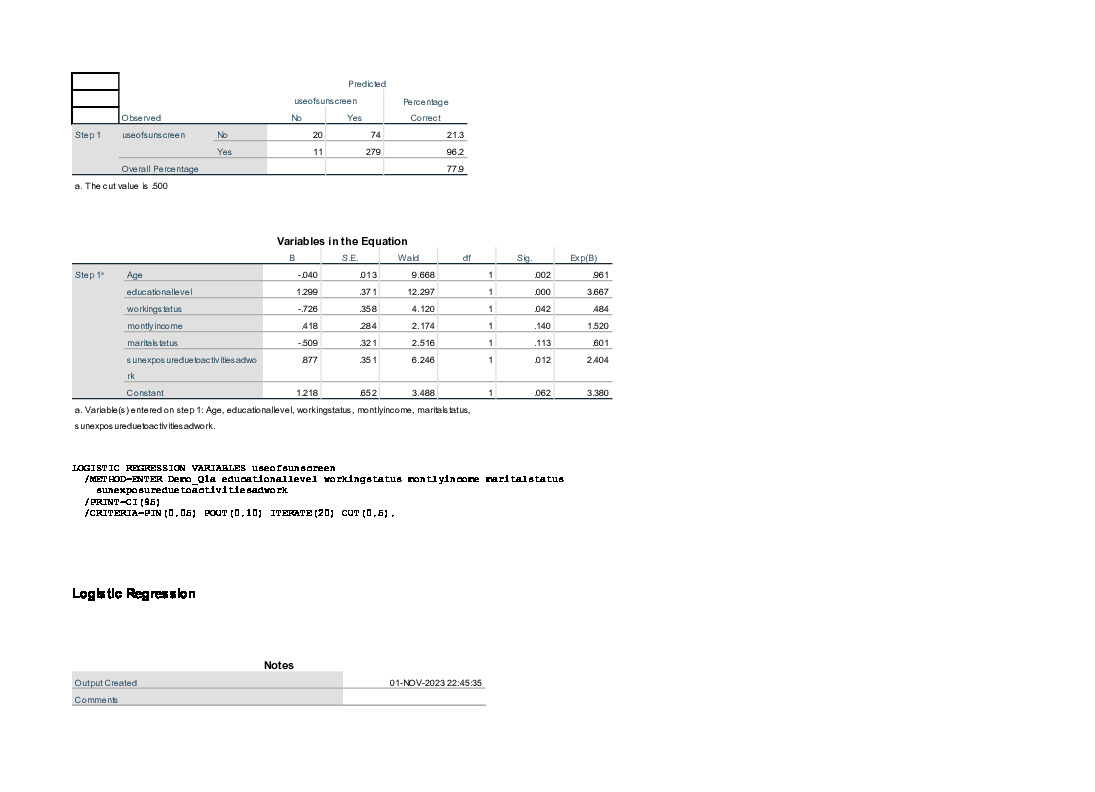

Supplement: S1 Fig — (ZIP) [file pone.0293896.s001.zip › Outputs - word file 67.tiff]

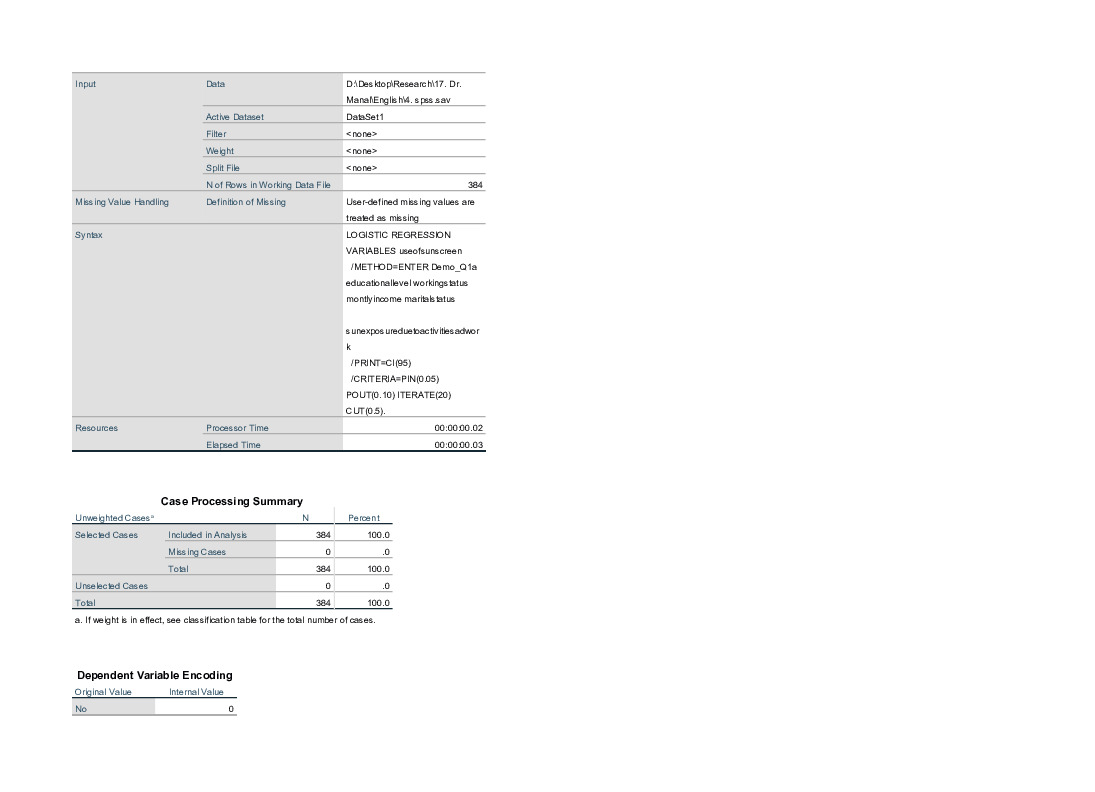

Supplement: S1 Fig — (ZIP) [file pone.0293896.s001.zip › Outputs - word file 68.tiff]

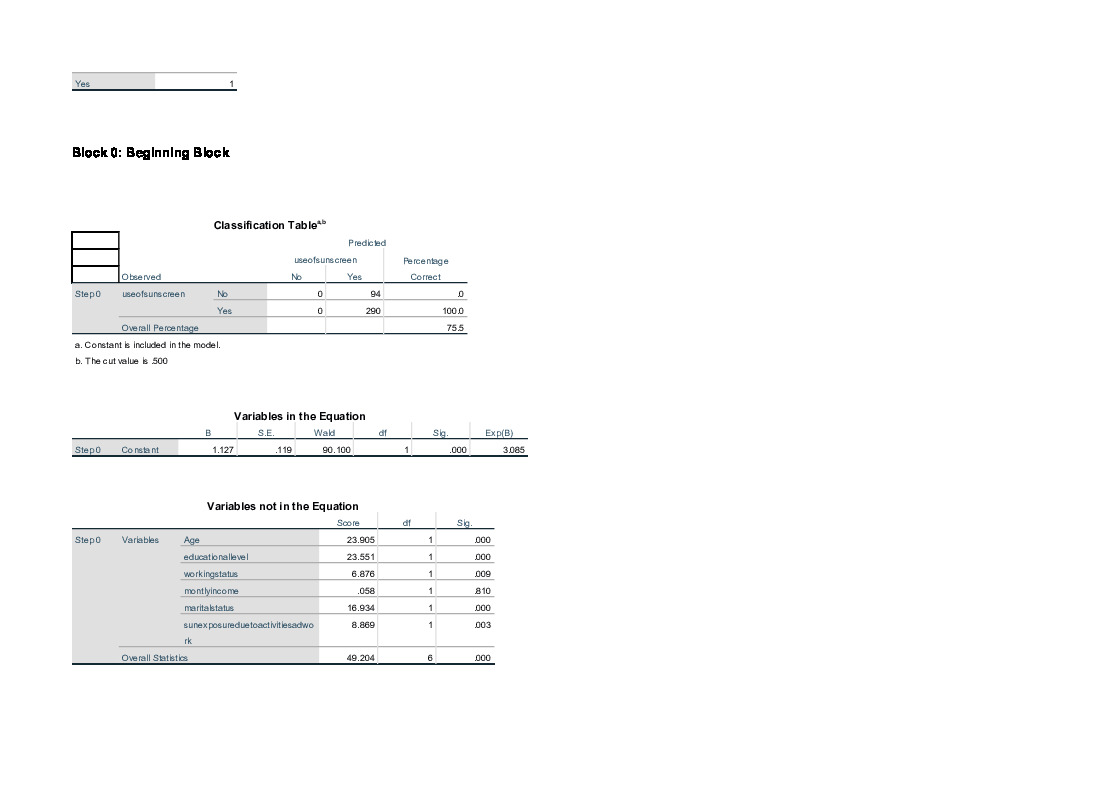

Supplement: S1 Fig — (ZIP) [file pone.0293896.s001.zip › Outputs - word file 69.tiff]

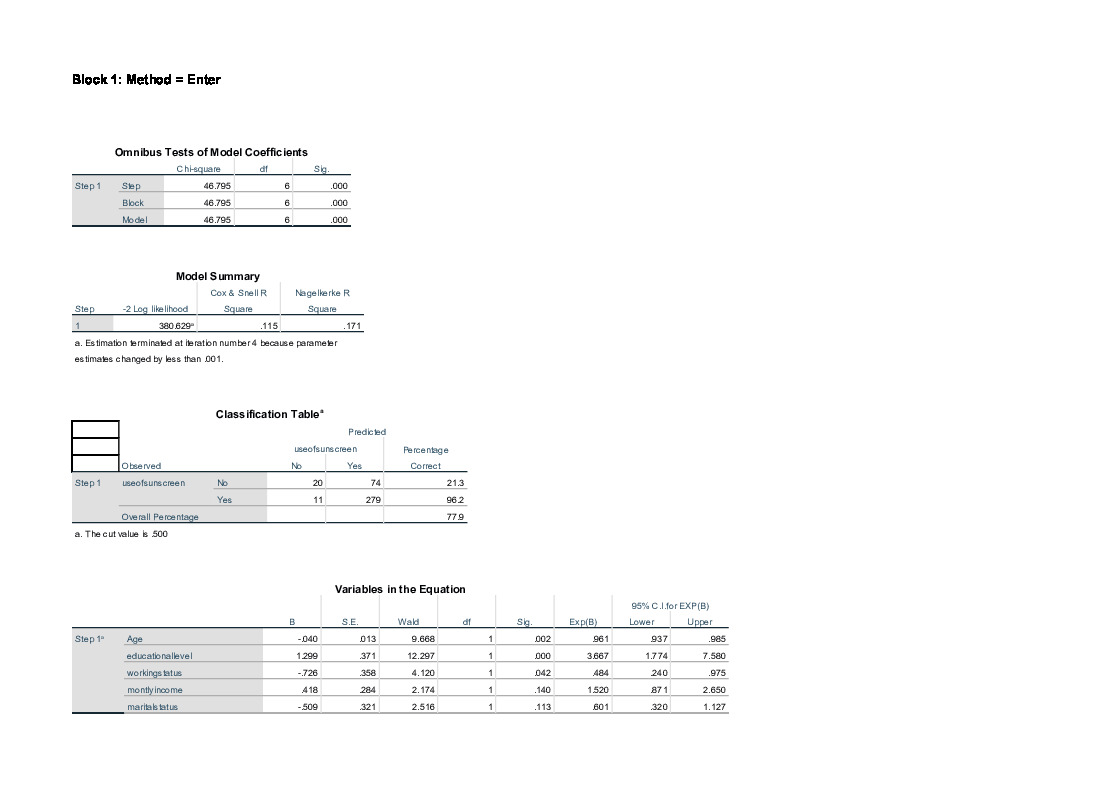

Supplement: S1 Fig — (ZIP) [file pone.0293896.s001.zip › Outputs - word file 70.tiff]

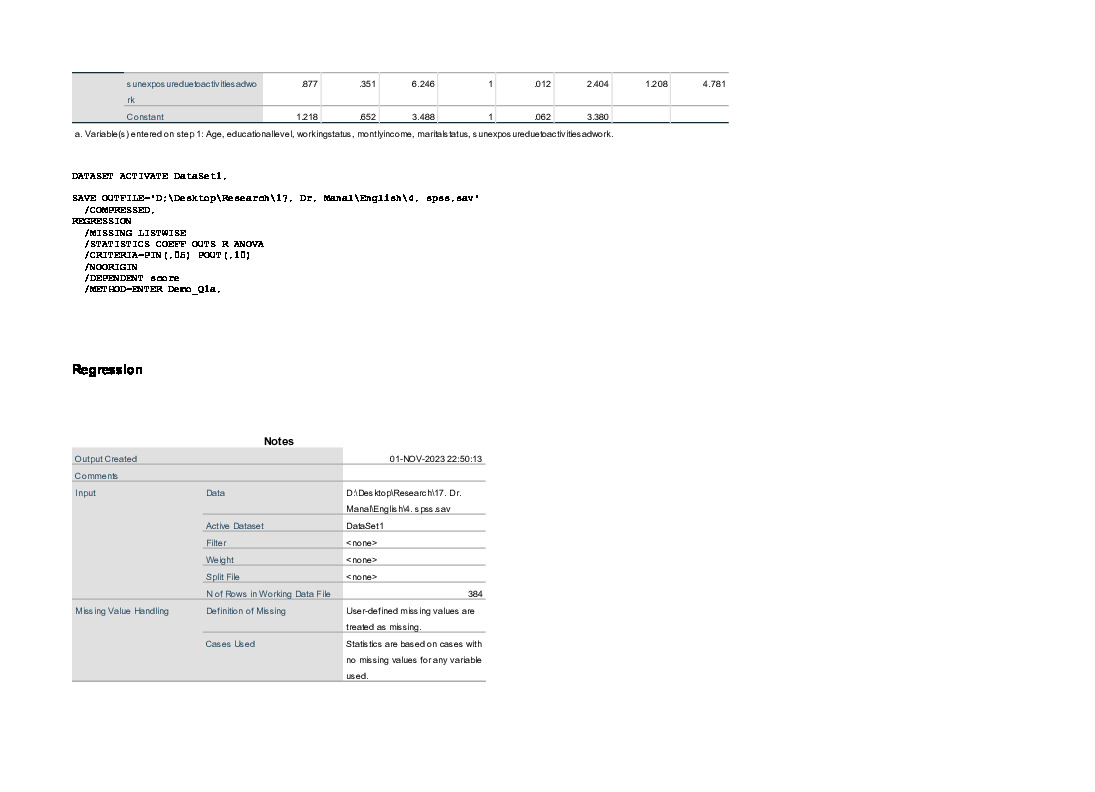

Supplement: S1 Fig — (ZIP) [file pone.0293896.s001.zip › Outputs - word file 71.tiff]

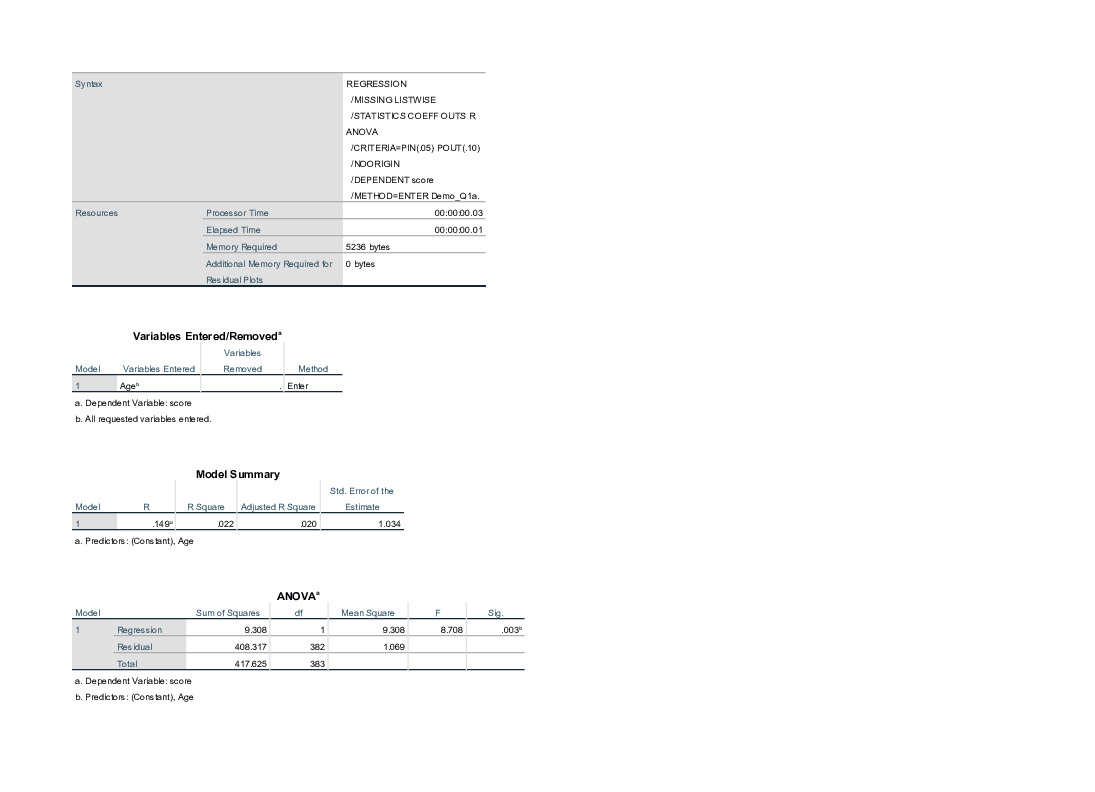

Supplement: S1 Fig — (ZIP) [file pone.0293896.s001.zip › Outputs - word file 72.tiff]

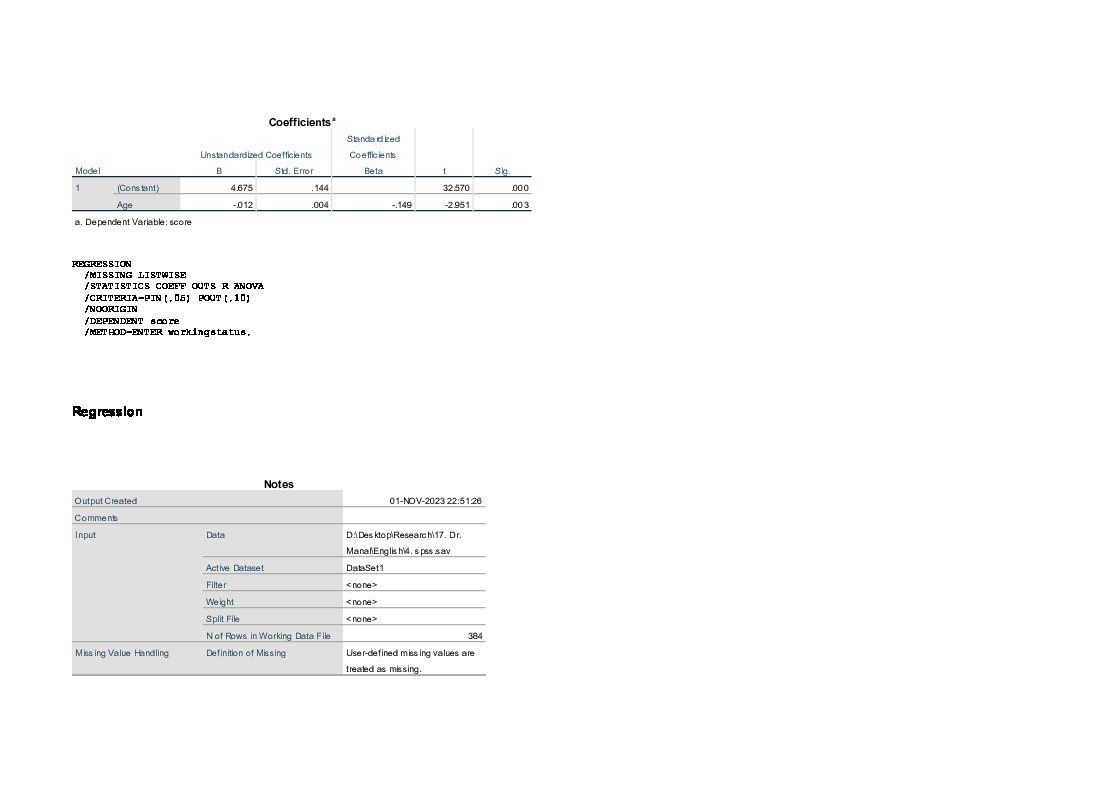

Supplement: S1 Fig — (ZIP) [file pone.0293896.s001.zip › Outputs - word file 73.tiff]

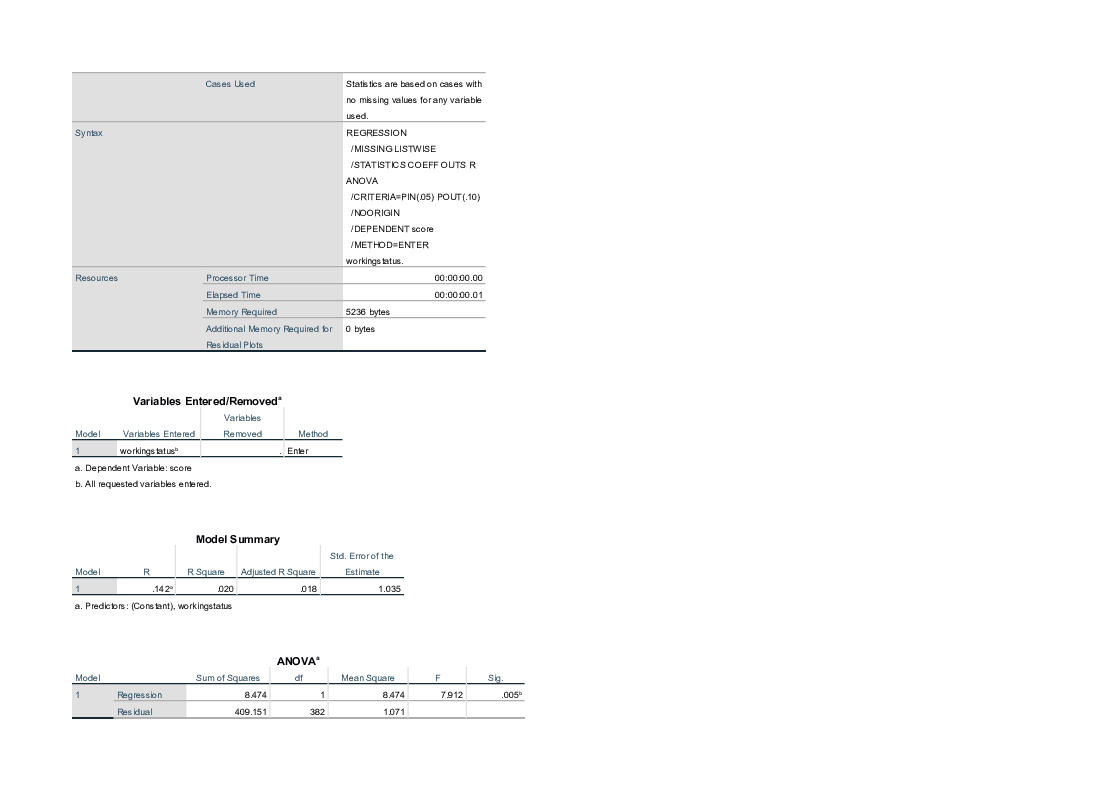

Supplement: S1 Fig — (ZIP) [file pone.0293896.s001.zip › Outputs - word file 74.tiff]

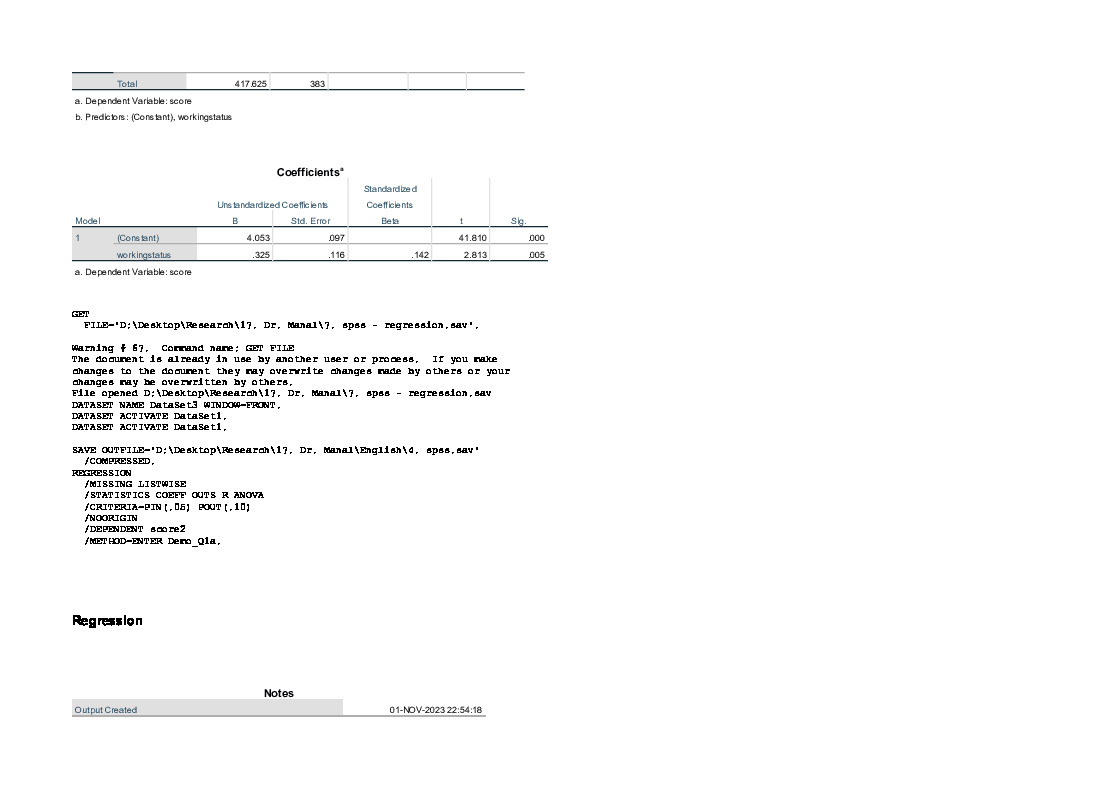

Supplement: S1 Fig — (ZIP) [file pone.0293896.s001.zip › Outputs - word file 75.tiff]

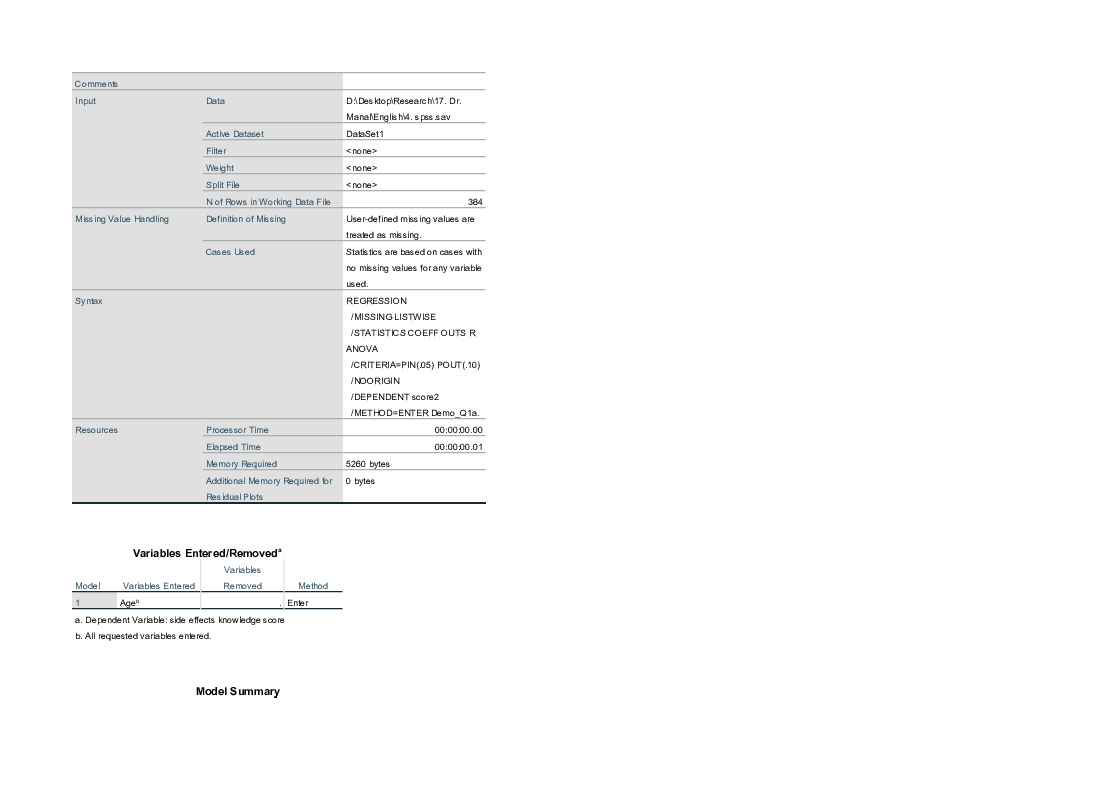

Supplement: S1 Fig — (ZIP) [file pone.0293896.s001.zip › Outputs - word file 76.tiff]

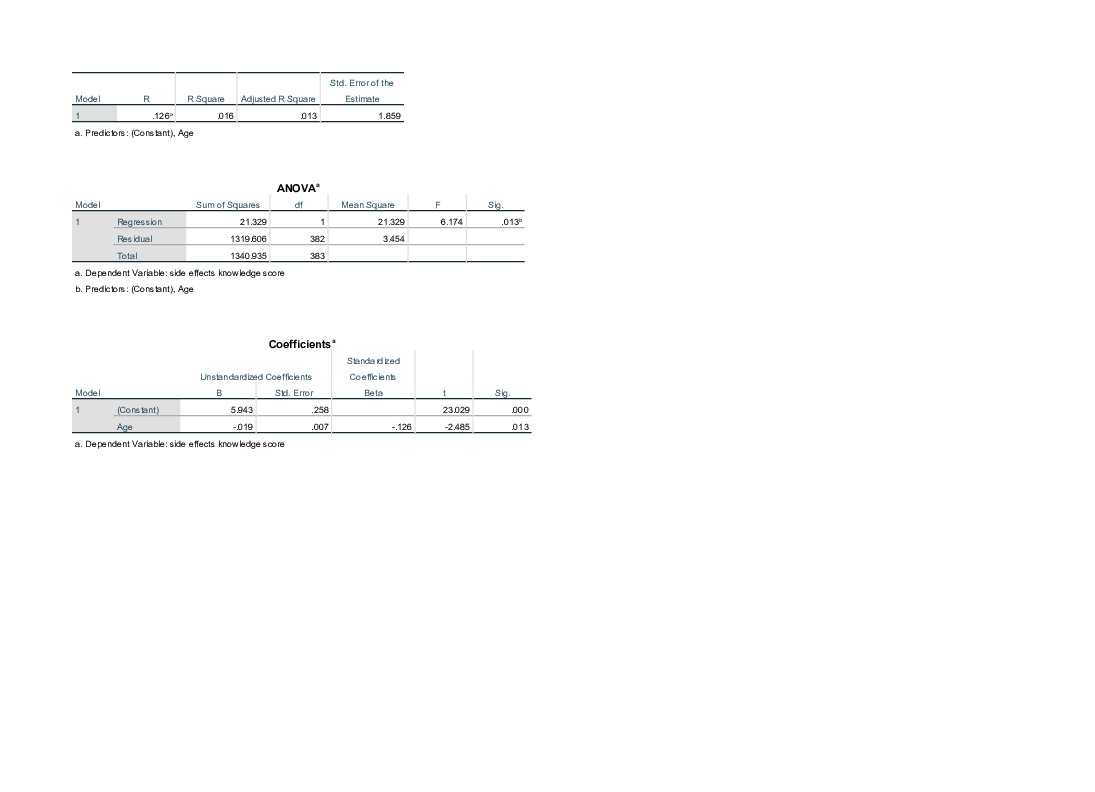

Supplement: S1 Fig — (ZIP) [file pone.0293896.s001.zip › Outputs - word file 77.tiff]
