# Supplementary material for: Skin-lightening products and Jordanian women: Beliefs and practice. A cross-sectional study
Source: PLoS One. 2023 Nov 21;18(11):e0293896. doi: 10.1371/journal.pone.0293896 (PMC10662732; doi:10.1371/journal.pone.0293896)
Supplement: S1 File — (PDF) [file pone.0293896.s002.pdf]

Descriptive Statistics

	N	Minimum	Maximum	Mean	Std. Deviation
Age	384	16	69	32.04	12.678
Valid N (listwise)	384				

Statistics

		Place of residence	Educational level	Working status	Monthly income	Marital status	Smoking status
N	Valid	384	384	384	384	384	384
	Missing	0	0	0	0	0	0

Place of residence

		Frequency	Percent	Valid Percent	Cumulative Percent
Valid	Central region (Amman, Zarqa, Balqa, Madaba)	367	95.6	95.6	95.6
	North region (Irbid, Ajloun, Jerash, Mafraq)	5	1.3	1.3	96.9
	South region (Karak, Tafilah, Ma`an, Aqaba)	12	3.1	3.1	100.0
	Total	384	100.0	100.0	

Educational level

		Frequency	Percent	Valid Percent	Cumulative Percent
Valid	Bachelor`s degree	288	75.0	75.0	75.0
	Diploma	29	7.6	7.6	82.6
	Master`s degree	33	8.6	8.6	91.1
	PhD	13	3.4	3.4	94.5
	Secondary education or les	21	5.5	5.5	100.0
	Total	384	100.0	100.0	

Working status

		Frequency	Percent	Valid Percent	Cumulative Percent
Valid	Not working	114	29.7	29.7	29.7
	Student	135	35.2	35.2	64.8
	working	135	35.2	35.2	100.0
	Total	384	100.0	100.0	

Monthly income

		Frequency	Percent	Valid Percent	Cumulative Percent
Valid	251-500 JDs	91	23.7	23.7	23.7
	501-750 JDs	56	14.6	14.6	38.3
	751-1000 JDs	36	9.4	9.4	47.7
	Less than or equal to 250 JDs	146	38.0	38.0	85.7
	More than 1000 JDs	55	14.3	14.3	100.0
	Total	384	100.0	100.0	

Marital status

		Frequency	Percent	Valid Percent	Cumulative Percent
Valid	Married	159	41.4	41.4	41.4
	Others(divorced/widowed)	18	4.7	4.7	46.1
	Single	207	53.9	53.9	100.0
	Total	384	100.0	100.0	

Smoking status

		Frequency	Percent	Valid Percent	Cumulative Percent
Valid	Ex-smoker	6	1.6	1.6	1.6
	Not a smoker	318	82.8	82.8	84.4
	Smoker	60	15.6	15.6	100.0
	Total	384	100.0	100.0	

Statistics

Q2- What is your natural skin tone?

N	Valid	384
	Missing	0

Q2- What is your natural skin tone?

		Frequency	Percent	Valid Percent	Cumulative Percent
Valid	Dark brown	2	.5	.5	.5
	Fair	177	46.1	46.1	46.6
	Light brown	146	38.0	38.0	84.6
	Very fair	59	15.4	15.4	100.0
	Total	384	100.0	100.0	

Statistics

		Q3- Does your daily life activities/work predispose you to high sunlight exposure?	Q4- Do you regularly use sunscreen or sunblock products?	Q5- Have you ever had skin discolorations such as melasma, freckles or sunburn?	Q6- Do you allocate part of your income for purchasing skin-lightening products?	Q7- Do you use skin-lightening/lightening agents?	Q8- Have you ever tried using home-made preparations (herbs, lemon) to whiten/lighten your skin?
N	Valid	384	384	384	384	384	384
	Missing	0	0	0	0	0	0

Q3- Does your daily life activities/work predispose you to high sunlight exposure?

		Frequency	Percent	Valid Percent	Cumulative Percent
Valid	No	51	13.3	13.3	13.3
	Yes, largely	43	11.2	11.2	24.5
	yes, moderately	161	41.9	41.9	66.4
	yes, partially	129	33.6	33.6	100.0
	Total	384	100.0	100.0	

Q4- Do you regularly use sunscreen or sunblock products?

		Frequency	Percent	Valid Percent	Cumulative Percent
Valid	No	94	24.5	24.5	24.5
	Yes	290	75.5	75.5	100.0
	Total	384	100.0	100.0	

Q5- Have you ever had skin discolorations such as melasma, freckles or sunburn?

		Frequency	Percent	Valid Percent	Cumulative Percent
Valid	Never	82	21.4	21.4	21.4
	Yes, largely	28	7.3	7.3	28.6
	Yes, moderately	109	28.4	28.4	57.0
	Yes, partially	165	43.0	43.0	100.0
	Total	384	100.0	100.0	

Q6- Do you allocate part of your income for purchasing skin-lightening products?

		Frequency	Percent	Valid Percent	Cumulative Percent
Valid	No	277	72.1	72.1	72.1
	Yes	107	27.9	27.9	100.0
	Total	384	100.0	100.0	

Q7- Do you use skin-lightening/lightening agents?

		Frequency	Percent	Valid Percent	Cumulative Percent
Valid	I used it before	124	32.3	32.3	32.3
	Never used it	191	49.7	49.7	82.0
	Yes, I use it currently	69	18.0	18.0	100.0
	Total	384	100.0	100.0	

Q8- Have you ever tried using home-made preparations (herbs, lemon) to whiten/lighten your skin?

		Frequency	Percent	Valid Percent	Cumulative Percent
Valid	No	262	68.2	68.2	68.2
	Yes	122	31.8	31.8	100.0
	Total	384	100.0	100.0	

Statistics

		Q9a- Which of the following factors most impact your choice of the skin-lightening products (Cost of the product)?	Q9b- Which of the following factors most impact your choice of the skin-lightening products (Brand name)?	Q9c- Which of the following factors most impact your choice of the skin-lightening products (Active ingredients)?
N	Valid	384	384	384
	Missing	0	0	0

Q9a- Which of the following factors most impact your choice of the skin-lightening products (Cost of the product)?

		Frequency	Percent	Valid Percent	Cumulative Percent
Valid	No	96	25.0	25.0	25.0
	Yes	288	75.0	75.0	100.0
	Total	384	100.0	100.0	

Q9b- Which of the following factors most impact your choice of the skin-lightening products (Brand name)?

		Frequency	Percent	Valid Percent	Cumulative Percent
Valid	No	59	15.4	15.4	15.4
	Yes	325	84.6	84.6	100.0
	Total	384	100.0	100.0	

Q9c- Which of the following factors most impact your choice of the skin-lightening products (Active ingredients)?

		Frequency	Percent	Valid Percent	Cumulative Percent
Valid	No	59	15.4	15.4	15.4
	Yes	325	84.6	84.6	100.0
	Total	384	100.0	100.0	

Statistics

		Q10a- Which of the following sources you rely on to obtain information about skin-lightening products (TV)?	Q10b- Which of the following sources you rely on to obtain information about skin-lightening products (Dermatologists)?	Q10c- Which of the following sources you rely on to obtain information about skin-lightening products (community pharmacists)?	Q10d- Which of the following sources you rely on to obtain information about skin-lightening products (beauty experts)?	Q10e- Which of the following sources you rely on to obtain information about skin-lightening products (cosmetics websites)?	Q10f- Which of the following sources you rely on to obtain information about skin-lightening products (social media)?	Q10g- Which of the following sources you rely on to obtain information about skin-lightening products (Friends)?
N	Valid	384	384	384	384	384	384	384
	Missing	0	0	0	0	0	0	0

Q10a- Which of the following sources you rely on to obtain information about skin-lightening products (TV)?

		Frequency	Percent	Valid Percent	Cumulative Percent
Valid	No	347	90.4	90.4	90.4
	Yes	37	9.6	9.6	100.0
	Total	384	100.0	100.0	

Q10b- Which of the following sources you rely on to obtain information about skin-lightening products (Dermatologists)?

		Frequency	Percent	Valid Percent	Cumulative Percent
Valid	No	79	20.6	20.6	20.6
	Yes	305	79.4	79.4	100.0
	Total	384	100.0	100.0	

Q10c- Which of the following sources you rely on to obtain information about skin-lightening products (community pharmacists)?

		Frequency	Percent	Valid Percent	Cumulative Percent
Valid	No	92	24.0	24.0	24.0
	Yes	292	76.0	76.0	100.0
	Total	384	100.0	100.0	

Q10d- Which of the following sources you rely on to obtain information about skin-lightening products (beauty experts)?

		Frequency	Percent	Valid Percent	Cumulative Percent
Valid	No	153	39.8	39.8	39.8
	Yes	231	60.2	60.2	100.0
	Total	384	100.0	100.0	

Q10e- Which of the following sources you rely on to obtain information about skin-lightening products (cosmetics websites)?

		Frequency	Percent	Valid Percent	Cumulative Percent
Valid	No	188	49.0	49.0	49.0
	Yes	196	51.0	51.0	100.0
	Total	384	100.0	100.0	

Q10f- Which of the following sources you rely on to obtain information about skin-lightening products (social media)?

		Frequency	Percent	Valid Percent	Cumulative Percent
Valid	No	200	52.1	52.1	52.1
	Yes	184	47.9	47.9	100.0
	Total	384	100.0	100.0	

Q10g- Which of the following sources you rely on to obtain information about skin-lightening products (Friends)?

		Frequency	Percent	Valid Percent	Cumulative Percent
Valid	No	144	37.5	37.5	37.5
	Yes	240	62.5	62.5	100.0
	Total	384	100.0	100.0	

Statistics

		Q11a- Which of the following substances do you think are used in skin-lightening products (glutahione)?	Q11b- Which of the following substances do you think are used in skin-lightening products (vitamin C)?	Q11c- Which of the following substances do you think are used in skin-lightening products (Hydroquinone) ?	Q11d- Which of the following substances do you think are used in skin-lightening products (Arbutin)?	Q11e- Which of the following substances do you think are used in skin-lightening products (Kojic acid)?
N	Valid	384	384	384	384	384

Missing	0	0	0	0	0

Q11a- Which of the following substances do you think are used in skin-lightening products (glutahione)?

		Frequency	Percent	Valid Percent	Cumulative Percent
Valid	No	242	63.0	63.0	63.0
	Yes	142	37.0	37.0	100.0
	Total	384	100.0	100.0	

Q11b- Which of the following substances do you think are used in skin-lightening products (vitamin C)?

		Frequency	Percent	Valid Percent	Cumulative Percent
Valid	No	47	12.2	12.2	12.2
	Yes	337	87.8	87.8	100.0
	Total	384	100.0	100.0	

Q11c- Which of the following substances do you think are used in skin-lightening products (Hydroquinone)?

		Frequency	Percent	Valid Percent	Cumulative Percent
Valid	No	146	38.0	38.0	38.0
	Yes	238	62.0	62.0	100.0
	Total	384	100.0	100.0	

Q11d- Which of the following substances do you think are used in skin-lightening products (Arbutin)?

		Frequency	Percent	Valid Percent	Cumulative Percent
Valid	No	251	65.4	65.4	65.4
	Yes	133	34.6	34.6	100.0

	Total	384	100.0	100.0	

Q11e- Which of the following substances do you think are used in skin-lightening products (Kojic acid)?

		Frequency	Percent	Valid Percent	Cumulative Percent
Valid	No	192	50.0	50.0	50.0
	Yes	192	50.0	50.0	100.0
	Total	384	100.0	100.0	

Statistics

		Q12a-What is the source of your information about the substances mentioned in the previous question (Information provided on the product`s leaflet)?	Q12b-What is the source of your information about the substances mentioned in the previous question (Friends)?	Q12c-What is the source of your information about the substances mentioned in the previous question (Advertisements)?	Q12d-What is the source of your information about the substances mentioned in the previous question (Social media)?	Q12e-What is the source of your information about the substances mentioned in the previous question (Beauty centers)?	Q12f-What is the source of your information about the substances mentioned in the previous question (Educational institutions (universities, training centers))?
N	Valid	384	384	384	384	384	384
	Missing	0	0	0	0	0	0

Q12a-What is the source of your information about the substances mentioned in the previous question (Information provided on the product`s leaflet)?

		Frequency	Percent	Valid Percent	Cumulative Percent
Valid	No	91	23.7	23.7	23.7

		Frequency	Percent	Valid Percent	Cumulative Percent
	Yes	293	76.3	76.3	100.0
	Total	384	100.0	100.0	

Q12b-What is the source of your information about the substances mentioned in the previous question (Friends)?

		Frequency	Percent	Valid Percent	Cumulative Percent
Valid	No	212	55.2	55.2	55.2
	Yes	172	44.8	44.8	100.0
	Total	384	100.0	100.0	

Q12c-What is the source of your information about the substances mentioned in the previous question (Advertisements)?

		Frequency	Percent	Valid Percent	Cumulative Percent
Valid	No	224	58.3	58.3	58.3
	Yes	160	41.7	41.7	100.0
	Total	384	100.0	100.0	

Q12d-What is the source of your information about the substances mentioned in the previous question (Social media)?

		Frequency	Percent	Valid Percent	Cumulative Percent
Valid	No	182	47.4	47.4	47.4
	Yes	202	52.6	52.6	100.0
	Total	384	100.0	100.0	

Q12e-What is the source of your information about the substances mentioned in the previous question (Beauty centers)?

		Frequency	Percent	Valid Percent	Cumulative Percent
Valid	No	213	55.5	55.5	55.5
	Yes	171	44.5	44.5	100.0
	Total	384	100.0	100.0	

Q12f-What is the source of your information about the substances mentioned in the previous question (Educational institutions (universities, training centers))?

		Frequency	Percent	Valid Percent	Cumulative Percent
Valid	No	210	54.7	54.7	54.7
	Yes	174	45.3	45.3	100.0
	Total	384	100.0	100.0	

Statistics

Q-13 Do you think there are side effects to skin-lightening/lightening products?

N	Valid	384
	Missing	0

Q-13 Do you think there are side effects to skin-lightening/lightening products?

		Frequency	Percent	Valid Percent	Cumulative Percent
Valid	No	45	11.7	11.7	11.7
	Yes	339	88.3	88.3	100.0
	Total	384	100.0	100.0	

Statistics

Q14- Have you suffered any side effects upon using these products?

N	Valid	384
	Missing	0

Q14- Have you suffered any side effects upon using these products?

		Frequency	Percent	Valid Percent	Cumulative Percent
Valid	No	314	81.8	81.8	81.8
	Yes	70	18.2	18.2	100.0
	Total	384	100.0	100.0	

Statistics

		Q15a- Which of the following do you think may arise as a side effect as a result of using skin-lightening/lightening products (Skin atrophy)?	Q15b- Which of the following do you think may arise as a side effect as a result of using skin-lightening/lightening products (Dermatitis)?	Q15c- Which of the following do you think may arise as a side effect as a result of using skin-lightening/lightening products (Stomatitis)?	Q15d- Which of the following do you think may arise as a side effect as a result of using skin-lightening/lightening products (Acne)?	Q15e- Which of the following do you think may arise as a side effect as a result of using skin-lightening/lightening products (Skin redness)?	Q15f- Which of the following do you think may arise as a side effect as a result of using skin-lightening/lightening products (Light sensitivity)?	Q15g- Which of the following do you think may arise as a side effect as a result of using skin-lightening/lightening products (Skin irritation)?	Q15h- Which of the following do you think may arise as a side effect as a result of using skin-lightening/lightening products (New dark spots)?
N	Valid	384	384	384	384	384	384	384	384
	Missing	0	0	0	0	0	0	0	0

Q15a- Which of the following do you think may arise as a side effect as a result of using skin-lightening/lightening products (Skin atrophy)?

		Frequency	Percent	Valid Percent	Cumulative Percent
Valid	No	282	73.4	73.4	73.4
	Yes	102	26.6	26.6	100.0
	Total	384	100.0	100.0	

Q15b- Which of the following do you think may arise as a side effect as a result of using skin-lightening/lightening products (Dermatitis)?

		Frequency	Percent	Valid Percent	Cumulative Percent
Valid	No	134	34.9	34.9	34.9
	Yes	250	65.1	65.1	100.0
	Total	384	100.0	100.0	

Q15c- Which of the following do you think may arise as a side effect as a result of using skin-lightening/lightening products (Stomatitis)?

		Frequency	Percent	Valid Percent	Cumulative Percent
Valid	No	172	44.8	44.8	44.8
	Yes	212	55.2	55.2	100.0
	Total	384	100.0	100.0	

Q15d- Which of the following do you think may arise as a side effect as a result of using skin-lightening/lightening products (Acne)?

		Frequency	Percent	Valid Percent	Cumulative Percent

		Frequency	Percent	Valid Percent	Cumulative Percent
Valid	No	109	28.4	28.4	28.4
	Yes	275	71.6	71.6	100.0
	Total	384	100.0	100.0	

Q15e- Which of the following do you think may arise as a side effect as a result of using skin-lightening/lightening products (Skin redness)?

		Frequency	Percent	Valid Percent	Cumulative Percent
Valid	No	35	9.1	9.1	9.1
	Yes	349	90.9	90.9	100.0
	Total	384	100.0	100.0	

Q15f- Which of the following do you think may arise as a side effect as a result of using skin-lightening/lightening products (Light sensitivity)?

		Frequency	Percent	Valid Percent	Cumulative Percent
Valid	No	109	28.4	28.4	28.4
	Yes	275	71.6	71.6	100.0
	Total	384	100.0	100.0	

Q15g- Which of the following do you think may arise as a side effect as a result of using skin-lightening/lightening products (Skin irritation)?

		Frequency	Percent	Valid Percent	Cumulative Percent
Valid	No	27	7.0	7.0	7.0
	Yes	357	93.0	93.0	100.0
	Total	384	100.0	100.0	

Q15h- Which of the following do you think may arise as a side effect as a result of using skin-lightening/lightening products (New dark spots)?

		Frequency	Percent	Valid Percent	Cumulative Percent
Valid	No	151	39.3	39.3	39.3
	Yes	233	60.7	60.7	100.0
	Total	384	100.0	100.0	

Statistics

Q16- In case any of these side effects arise, what would you do?

N	Valid	384
	Missing	0

Q16- In case any of these side effects arise, what would you do?

		Frequency	Percent	Valid Percent	Cumulative Percent
Valid		3	.8	.8	.8
	continue using the product	1	.3	.3	1.0
	stop using the product immediately and consult a dermatologist/ pharmacist/ beauty expert	241	62.8	62.8	63.8
	stop using the product immediately and never use it again	90	23.4	23.4	87.2
	stop using the product immediately and purchase a different product	49	12.8	12.8	100.0
	Total	384	100.0	100.0	

Statistics

		Q17a- In your opinion, the side effects result due to which of the following (The active ingredient/s)?	Q17b- In your opinion, the side effects result due to which of the following (Improper time of use of the product)?	Q17c- In your opinion, the side effects result due to which of the following (Excessive use of the product)?	Q17d- In your opinion, the side effects result due to which of the following (Lack of information on how to use the product)?	Q17e- In your opinion, the side effects result due to which of the following (Using more than one product at the same time)?
N	Valid	384	384	384	384	384
	Missing	0	0	0	0	0

Q17a- In your opinion, the side effects result due to which of the following (The active ingredient/s)?

		Frequency	Percent	Valid Percent	Cumulative Percent
Valid	No	38	9.9	9.9	9.9
	Yes	346	90.1	90.1	100.0
	Total	384	100.0	100.0	

Q17b- In your opinion, the side effects result due to which of the following (Improper time of use of the product)?

		Frequency	Percent	Valid Percent	Cumulative Percent
Valid	No	99	25.8	25.8	25.8
	Yes	285	74.2	74.2	100.0
	Total	384	100.0	100.0	

Q17c- In your opinion, the side effects result due to which of the following (Excessive use of the product)?

		Frequency	Percent	Valid Percent	Cumulative Percent
Valid	No	56	14.6	14.6	14.6
	Yes	328	85.4	85.4	100.0
	Total	384	100.0	100.0	

Q17d- In your opinion, the side effects result due to which of the following (Lack of information on how to use the product)?

		Frequency	Percent	Valid Percent	Cumulative Percent
Valid	No	39	10.2	10.2	10.2
	Yes	345	89.8	89.8	100.0
	Total	384	100.0	100.0	

Q17e- In your opinion, the side effects result due to which of the following (Using more than one product at the same time)?

		Frequency	Percent	Valid Percent	Cumulative Percent
Valid	No	44	11.5	11.5	11.5
	Yes	340	88.5	88.5	100.0
	Total	384	100.0	100.0	

Statistics

Q18- Which of the following interventions do you prefer to use to lighten your skin?

N	Valid	384
	Missing	0

Q18- Which of the following interventions do you prefer to use to lighten your skin?

		Frequency	Percent	Valid Percent	Cumulative Percent
Valid	Laser	56	14.6	14.6	14.6
	other	42	10.9	10.9	25.5
	Peeling	144	37.5	37.5	63.0
	Skin Whitening Products	142	37.0	37.0	100.0
	Total	384	100.0	100.0	

Statistics

Q19- Do you mind using these products if recommended by your dermatologist?

N	Valid	384
	Missing	0

Q19- Do you mind using these products if recommended by your dermatologist?

		Frequency	Percent	Valid Percent	Cumulative Percent
Valid	No	208	54.2	54.2	54.2
	Yes	176	45.8	45.8	100.0
	Total	384	100.0	100.0	

Statistics

Q20- What is the most suitable duration for using these products?

N	Valid	384
	Missing	0

Q20- What is the most suitable duration for using these products?

		Frequency	Percent	Valid Percent	Cumulative Percent
Valid	3-5 months	109	28.4	28.4	28.4
	6-12 months	41	10.7	10.7	39.1
	all the time	49	12.8	12.8	51.8
	I don't know	100	26.0	26.0	77.9
	less than 3 months	85	22.1	22.1	100.0
	Total	384	100.0	100.0	

Statistics

Q21- How frequent should these products be used?

N	Valid	384
	Missing	0

Q21- How frequent should these products be used?

		Frequency	Percent	Valid Percent	Cumulative Percent
Valid	I don't know	61	15.9	15.9	15.9
	once daily	179	46.6	46.6	62.5
	once weekly	44	11.5	11.5	74.0
	twice daily	100	26.0	26.0	100.0
	Total	384	100.0	100.0	

Statistics

		Q22a- Which of the following made you use/ would make you use these products (To lighten my skin cosmetically)?	Q22b- Which of the following made you use/ would make you use these products (To remove any skin pigmentation (melasma, dark spots, freckles))?	Q22c- Which of the following made you use/ would make you use these products (Others)?
N	Valid	384	384	384
	Missing	0	0	0

Q22a- Which of the following made you use/ would make you use these products (To lighten my skin cosmetically)?

		Frequency	Percent	Valid Percent	Cumulative Percent
Valid	No	194	50.5	50.5	50.5
	Yes	190	49.5	49.5	100.0
	Total	384	100.0	100.0	

Q22b- Which of the following made you use/ would make you use these products (To remove any skin pigmentation (melasma, dark spots, freckles))?

		Frequency	Percent	Valid Percent	Cumulative Percent
Valid	No	47	12.2	12.2	12.2
	Yes	337	87.8	87.8	100.0
	Total	384	100.0	100.0	

Q22c- Which of the following made you use/ would make you use these products (Others)?

		Frequency	Percent	Valid Percent	Cumulative Percent
Valid	No	282	73.4	73.4	73.4
	Yes	102	26.6	26.6	100.0
	Total	384	100.0	100.0	

Statistics

		Q23a- Which of the following dosage forms/formulations do you prefer to you (Creams)?	Q23b- Which of the following dosage forms/formulations do you prefer to you (lotions)?	Q23c- Which of the following dosage forms/formulations do you prefer to you (Soaps)?	Q23d- Which of the following dosage forms/formulations do you prefer to you (Injections)?	Q23e- Which of the following dosage forms/formulations do you prefer to you (Pills)?	Q23f- Which of the following dosage forms/formulations do you prefer to you (Serum)?
N	Valid	384	384	384	384	384	384
	Missing	0	0	0	0	0	0

Q23a- Which of the following dosage forms/formulations do you prefer to you (Creams)?

		Frequency	Percent	Valid Percent	Cumulative Percent
Valid	No	22	5.7	5.7	5.7
	Yes	362	94.3	94.3	100.0
	Total	384	100.0	100.0	

Q23b- Which of the following dosage forms/formulations do you prefer to you (lotions)?

		Frequency	Percent	Valid Percent	Cumulative Percent
Valid	No	109	28.4	28.4	28.4
	Yes	275	71.6	71.6	100.0
	Total	384	100.0	100.0	

Q23c- Which of the following dosage forms/formulations do you prefer to you (Soaps)?

		Frequency	Percent	Valid Percent	Cumulative Percent
Valid	No	190	49.5	49.5	49.5
	Yes	194	50.5	50.5	100.0
	Total	384	100.0	100.0	

Q23d- Which of the following dosage forms/formulations do you prefer to you (Injections)?

		Frequency	Percent	Valid Percent	Cumulative Percent
Valid	No	347	90.4	90.4	90.4
	Yes	37	9.6	9.6	100.0
	Total	384	100.0	100.0	

Q23e- Which of the following dosage forms/formulations do you prefer to you (Pills)?

		Frequency	Percent	Valid Percent	Cumulative Percent
Valid	No	328	85.4	85.4	85.4
	Yes	56	14.6	14.6	100.0
	Total	384	100.0	100.0	

Q23f- Which of the following dosage forms/formulations do you prefer to you (Serum)?

		Frequency	Percent	Valid Percent	Cumulative Percent
Valid	No	84	21.9	21.9	21.9
	Yes	300	78.1	78.1	100.0
	Total	384	100.0	100.0	

Statistics

Q24- At which of the following body areas can these products be applied?

N	Valid	384
	Missing	0

Q24- At which of the following body areas can these products be applied?

		Frequency	Percent	Valid Percent	Cumulative Percent
Valid	Face and other parts of the body	281	73.2	73.2	73.2
	Face only	103	26.8	26.8	100.0
	Total	384	100.0	100.0	

Logistic Regression

Case Processing Summary

Unweighted Cases[a]		N	Percent
Selected Cases	Included in Analysis	384	100.0
	Missing Cases	0	.0
	Total	384	100.0
Unselected Cases		0	.0
Total		384	100.0

a. If weight is in effect, see classification table for the total number of cases.

Dependent Variable Encoding

Original Value	Internal Value
No	0

Yes	1

Classification Table[a,b]

Observed			Predicted: useofsunscreen No	Predicted: useofsunscreen Yes	Percentage Correct
Step 0	useofsunscreen	No	0	94	.0
		Yes	0	290	100.0
	Overall Percentage				75.5

a. Constant is included in the model.

b. The cut value is .500

Variables in the Equation

		B	S.E.	Wald	df	Sig.	Exp(B)
Step 0	Constant	1.127	.119	90.100	1	.000	3.085

Variables not in the Equation

			Score	df	Sig.
Step 0	Variables	Age	23.905	1	.000
	Overall Statistics		23.905	1	.000

Omnibus Tests of Model Coefficients

		Chi-square	df	Sig.
Step 1	Step	22.832	1	.000
	Block	22.832	1	.000
	Model	22.832	1	.000

Model Summary

Step	-2 Log likelihood	Cox & Snell R Square	Nagelkerke R Square
1	404.591[a]	.058	.086

a. Estimation terminated at iteration number 4 because parameter estimates changed by less than .001.

Classification Table[a]

Observed			Predicted: useofsunscreen No	Predicted: useofsunscreen Yes	Percentage Correct
Step 1	useofsunscreen	No	7	87	7.4
		Yes	5	285	98.3
	Overall Percentage				76.0

a. The cut value is .500

Variables in the Equation

		B	S.E.	Wald	df	Sig.	Exp(B)
Step 1[a]	Age	-.043	.009	22.401	1	.000	.957
	Constant	2.592	.346	56.181	1	.000	13.360

a. Variable(s) entered on step 1: Age.

Logistic Regression

Case Processing Summary

Unweighted Cases[a]		N	Percent
Selected Cases	Included in Analysis	384	100.0
	Missing Cases	0	.0
	Total	384	100.0
Unselected Cases		0	.0
Total		384	100.0

a. If weight is in effect, see classification table for the total number of cases.

Dependent Variable Encoding

Original Value	Internal Value
No	0

Yes	1

Classification Table[a,b]

Observed			Predicted: useofsunscreen No	Predicted: useofsunscreen Yes	Percentage Correct
Step 0	useofsunscreen	No	0	94	.0
		Yes	0	290	100.0
	Overall Percentage				75.5

a. Constant is included in the model.

b. The cut value is .500

Variables in the Equation

		B	S.E.	Wald	df	Sig.	Exp(B)
Step 0	Constant	1.127	.119	90.100	1	.000	3.085

Variables not in the Equation

			Score	df	Sig.
Step 0	Variables	placeofresidence	.449	1	.503
	Overall Statistics		.449	1	.503

Omnibus Tests of Model Coefficients

		Chi-square	df	Sig.
Step 1	Step	.481	1	.488
	Block	.481	1	.488
	Model	.481	1	.488

Model Summary

Step	-2 Log likelihood	Cox & Snell R Square	Nagelkerke R Square
1	426.942[a]	.001	.002

a. Estimation terminated at iteration number 4 because parameter estimates changed by less than .001.

Classification Table[a]

Observed			Predicted: useofsunscreen No	Predicted: useofsunscreen Yes	Percentage Correct
Step 1	useofsunscreen	No	0	94	.0
		Yes	0	290	100.0
	Overall Percentage				75.5

a. The cut value is .500

Variables in the Equation

		B	S.E.	Wald	df	Sig.	Exp(B)
Step 1[a]	placeofresidence	-.431	.648	.443	1	.506	.650
	Constant	1.540	.636	5.863	1	.015	4.667

a. Variable(s) entered on step 1: placeofresidence.

Logistic Regression

Case Processing Summary

Unweighted Cases[a]		N	Percent
Selected Cases	Included in Analysis	384	100.0
	Missing Cases	0	.0
	Total	384	100.0
Unselected Cases		0	.0
Total		384	100.0

a. If weight is in effect, see classification table for the total number of cases.

Dependent Variable Encoding

Original Value	Internal Value

No	0
Yes	1

Classification Table[a,b]

Observed			Predicted: useofsunscreen No	Predicted: useofsunscreen Yes	Percentage Correct
Step 0	useofsunscreen	No	0	94	.0
		Yes	0	290	100.0
	Overall Percentage				75.5

a. Constant is included in the model.

b. The cut value is .500

Variables in the Equation

		B	S.E.	Wald	df	Sig.	Exp(B)
Step 0	Constant	1.127	.119	90.100	1	.000	3.085

Variables not in the Equation

			Score	df	Sig.
Step 0	Variables	educationallevel	23.551	1	.000
	Overall Statistics		23.551	1	.000

Omnibus Tests of Model Coefficients

		Chi-square	df	Sig.
Step 1	Step	20.619	1	.000
	Block	20.619	1	.000
	Model	20.619	1	.000

Model Summary

Step	-2 Log likelihood	Cox & Snell R Square	Nagelkerke R Square
1	406.804[a]	.052	.078

a. Estimation terminated at iteration number 4 because parameter estimates changed by less than .001.

Classification Table[a]

Observed			Predicted: useofsunscreen No	Predicted: useofsunscreen Yes	Predicted: Percentage Correct
Step 1	useofsunscreen	No	26	68	27.7
		Yes	24	266	91.7
	Overall Percentage				76.0

a. The cut value is .500

Variables in the Equation

		B	S.E.	Wald	df	Sig.	Exp(B)
Step 1[a]	educationallevel	1.444	.314	21.150	1	.000	4.238
	Constant	-.080	.283	.080	1	.777	.923

a. Variable(s) entered on step 1: educationallevel.

Logistic Regression

Case Processing Summary

Unweighted Cases[a]		N	Percent
Selected Cases	Included in Analysis	384	100.0
	Missing Cases	0	.0
	Total	384	100.0
Unselected Cases		0	.0
Total		384	100.0

a. If weight is in effect, see classification table for the total number of cases.

Dependent Variable Encoding

Original Value	Internal Value
No	0
Yes	1

Classification Table[a,b]

Observed			Predicted		
			useofsunscreen		Percentage Correct
			No	Yes	
Step 0	useofsunscreen	No	0	94	.0
		Yes	0	290	100.0
	Overall Percentage				75.5

a. Constant is included in the model.

b. The cut value is .500

Variables in the Equation

		B	S.E.	Wald	df	Sig.	Exp(B)
Step 0	Constant	1.127	.119	90.100	1	.000	3.085

Variables not in the Equation

			Score	df	Sig.
Step 0	Variables	workingstatus	6.876	1	.009
	Overall Statistics		6.876	1	.009

Omnibus Tests of Model Coefficients

		Chi-square	df	Sig.
Step 1	Step	6.627	1	.010
	Block	6.627	1	.010
	Model	6.627	1	.010

Model Summary

Step	-2 Log likelihood	Cox & Snell R Square	Nagelkerke R Square
1	420.796[a]	.017	.025

a. Estimation terminated at iteration number 4 because parameter estimates changed by less than .001.

Classification Table[a]

Observed			Predicted: useofsunscreen No	Predicted: useofsunscreen Yes	Percentage Correct
Step 1	useofsunscreen	No	0	94	.0
		Yes	0	290	100.0
	Overall Percentage				75.5

a. The cut value is .500

Variables in the Equation

		B	S.E.	Wald	df	Sig.	Exp(B)
Step 1[a]	workingstatus	.647	.249	6.761	1	.009	1.911
	Constant	.693	.199	12.171	1	.000	2.000

a. Variable(s) entered on step 1: workingstatus.

Logistic Regression

Case Processing Summary

Unweighted Cases[a]		N	Percent
Selected Cases	Included in Analysis	384	100.0
	Missing Cases	0	.0
	Total	384	100.0
Unselected Cases		0	.0
Total		384	100.0

a. If weight is in effect, see classification table for the total number of cases.

Dependent Variable Encoding

Original Value	Internal Value
No	0
Yes	1

Classification Table[a,b]

			Predicted		
			useofsunscreen		Percentage
Observed			No	Yes	Correct
Step 0	useofsunscreen	No	0	94	.0
		Yes	0	290	100.0
	Overall Percentage				75.5

a. Constant is included in the model.

b. The cut value is .500

Variables in the Equation

		B	S.E.	Wald	df	Sig.	Exp(B)
Step 0	Constant	1.127	.119	90.100	1	.000	3.085

Variables not in the Equation

			Score	df	Sig.
Step 0	Variables	montlyincome	.058	1	.810
	Overall Statistics		.058	1	.810

Omnibus Tests of Model Coefficients

		Chi-square	df	Sig.
Step 1	Step	.058	1	.810
	Block	.058	1	.810
	Model	.058	1	.810

Model Summary

Step	-2 Log likelihood	Cox & Snell R Square	Nagelkerke R Square
1	427.365[a]	.000	.000

a. Estimation terminated at iteration number 4 because parameter estimates changed by less than .001.

Classification Table[a]

Observed			Predicted: useofsunscreen No	Predicted: useofsunscreen Yes	Predicted: Percentage Correct
Step 1	useofsunscreen	No	0	94	.0
		Yes	0	290	100.0
	Overall Percentage				75.5

a. The cut value is .500

Variables in the Equation

		B	S.E.	Wald	df	Sig.	Exp(B)
Step 1[a]	montlyincome	.059	.245	.058	1	.810	1.061
	Constant	1.104	.150	54.033	1	.000	3.017

a. Variable(s) entered on step 1: montlyincome.

Logistic Regression

Case Processing Summary

Unweighted Cases[a]		N	Percent
Selected Cases	Included in Analysis	384	100.0
	Missing Cases	0	.0
	Total	384	100.0
Unselected Cases		0	.0
Total		384	100.0

a. If weight is in effect, see classification table for the total number of cases.

Dependent Variable Encoding

Original Value	Internal Value
No	0
Yes	1

Classification Table[a,b]

	Observed		Predicted: useofsunscreen No	Predicted: useofsunscreen Yes	Percentage Correct
Step 0	useofsunscreen	No	0	94	.0
		Yes	0	290	100.0
	Overall Percentage				75.5

a. Constant is included in the model.

b. The cut value is .500

Variables in the Equation

		B	S.E.	Wald	df	Sig.	Exp(B)
Step 0	Constant	1.127	.119	90.100	1	.000	3.085

Variables not in the Equation

			Score	df	Sig.
Step 0	Variables	maritalstatus	16.934	1	.000
	Overall Statistics		16.934	1	.000

Omnibus Tests of Model Coefficients

		Chi-square	df	Sig.
Step 1	Step	16.751	1	.000
	Block	16.751	1	.000
	Model	16.751	1	.000

Model Summary

Step	-2 Log likelihood	Cox & Snell R Square	Nagelkerke R Square
1	410.672[a]	.043	.064

a. Estimation terminated at iteration number 4 because parameter estimates changed by less than .001.

Classification Table[a]

Observed			Predicted: useofsunscreen No	Predicted: useofsunscreen Yes	Percentage Correct
Step 1	useofsunscreen	No	0	94	.0
		Yes	0	290	100.0
	Overall Percentage				75.5

a. The cut value is .500

Variables in the Equation

		B	S.E.	Wald	df	Sig.	Exp(B)
Step 1[a]	maritalstatus	-.984	.243	16.352	1	.000	.374
	Constant	1.594	.178	80.197	1	.000	4.921

a. Variable(s) entered on step 1: maritalstatus.

Logistic Regression

Classification Table[a,b]

Observed			Predicted: useofsunscreen No	Predicted: useofsunscreen Yes	Percentage Correct
Step 0	useofsunscreen	No	0	94	.0
		Yes	0	290	100.0
	Overall Percentage				75.5

a. Constant is included in the model.

b. The cut value is .500

Variables in the Equation

	B	S.E.	Wald	df	Sig.	Exp(B)

Step 0	Constant	1.127	.119	90.100	1	.000	3.085

Variables not in the Equation

			Score	df	Sig.
Step 0	Variables	smokingstatus	.184	1	.668
	Overall Statistics		.184	1	.668

Omnibus Tests of Model Coefficients

		Chi-square	df	Sig.
Step 1	Step	.181	1	.670
	Block	.181	1	.670
	Model	.181	1	.670

Model Summary

Step	-2 Log likelihood	Cox & Snell R Square	Nagelkerke R Square
1	427.242[a]	.000	.001

a. Estimation terminated at iteration number 4 because parameter estimates changed by less than .001.

Classification Table[a]

			Predicted		
			useofsunscreen		Percentage Correct
Observed			No	Yes	
Step 1	useofsunscreen	No	0	94	.0
		Yes	0	290	100.0
	Overall Percentage				75.5

a. The cut value is .500

Variables in the Equation

		B	S.E.	Wald	df	Sig.	Exp(B)
Step 1[a]	smokingstatus	-.137	.320	.184	1	.668	.872

Constant	1.149	.130	78.134	1	.000	3.154

a. Variable(s) entered on step 1: smokingstatus.

Logistic Regression

Case Processing Summary

Unweighted Cases[a]		N	Percent
Selected Cases	Included in Analysis	384	100.0
	Missing Cases	0	.0
	Total	384	100.0
Unselected Cases		0	.0
Total		384	100.0

a. If weight is in effect, see classification table for the total number of cases.

Dependent Variable Encoding

Original Value	Internal Value
No	0
Yes	1

Classification Table[a,b]

			Predicted		
			useofsunscreen		Percentage
	Observed		No	Yes	Correct
Step 0	useofsunscreen	No	0	94	.0
		Yes	0	290	100.0
	Overall Percentage				75.5

a. Constant is included in the model.

b. The cut value is .500

Variables in the Equation

		B	S.E.	Wald	df	Sig.	Exp(B)
Step 0	Constant	1.127	.119	90.100	1	.000	3.085

Variables not in the Equation

			Score	df	Sig.
Step 0	Variables	sunexposureduetoactivitiesa dwork	8.869	1	.003
	Overall Statistics		8.869	1	.003

Omnibus Tests of Model Coefficients

		Chi-square	df	Sig.
Step 1	Step	8.057	1	.005
	Block	8.057	1	.005
	Model	8.057	1	.005

Model Summary

Step	-2 Log likelihood	Cox & Snell R Square	Nagelkerke R Square
1	419.366[a]	.021	.031

a. Estimation terminated at iteration number 4 because parameter estimates changed by less than .001.

Classification Table[a]

	Observed		Predicted useofsunscreen No	Predicted useofsunscreen Yes	Percentage Correct
Step 1	useofsunscreen	No	0	94	.0
		Yes	0	290	100.0
	Overall Percentage				75.5

a. The cut value is .500

Variables in the Equation

	B	S.E.	Wald	df	Sig.	Exp(B)

Step 1[a]	sunexposureduetoactivitiesadwork	.914	.314	8.473	1	.004	2.493
	Constant	.357	.285	1.572	1	.210	1.429

a. Variable(s) entered on step 1: sunexposureduetoactivitiesadwork.

Logistic Regression

Case Processing Summary

Unweighted Cases[a]		N	Percent
Selected Cases	Included in Analysis	384	100.0
	Missing Cases	0	.0
	Total	384	100.0
Unselected Cases		0	.0
Total		384	100.0

a. If weight is in effect, see classification table for the total number of cases.

Dependent Variable Encoding

Original Value	Internal Value
No	0
Yes	1

Classification Table[a,b]

	Observed		Predicted		
			useofsunscreen		Percentage Correct
			No	Yes	
Step 0	useofsunscreen	No	0	94	.0
		Yes	0	290	100.0
	Overall Percentage				75.5

a. Constant is included in the model.

b. The cut value is .500

Variables in the Equation

		B	S.E.	Wald	df	Sig.	Exp(B)
Step 0	Constant	1.127	.119	90.100	1	.000	3.085

Variables not in the Equation

			Score	df	Sig.
Step 0	Variables	Age	23.905	1	.000
		educationallevel	23.551	1	.000
		workingstatus	6.876	1	.009
		montlyincome	.058	1	.810
		maritalstatus	16.934	1	.000
		sunexposureduetoactivitiesadwork	8.869	1	.003
	Overall Statistics		49.204	6	.000

Omnibus Tests of Model Coefficients

		Chi-square	df	Sig.
Step 1	Step	46.795	6	.000
	Block	46.795	6	.000
	Model	46.795	6	.000

Model Summary

Step	-2 Log likelihood	Cox & Snell R Square	Nagelkerke R Square
1	380.629[a]	.115	.171

a. Estimation terminated at iteration number 4 because parameter estimates changed by less than .001.

Classification Table[a]

			Predicted		
			useofsunscreen		Percentage Correct
	Observed		No	Yes	
Step 1	useofsunscreen	No	20	74	21.3

	Yes	11	279	96.2
Overall Percentage				77.9

a. The cut value is .500

Variables in the Equation

		B	S.E.	Wald	df	Sig.	Exp(B)
Step 1[a]	Age	-.040	.013	9.668	1	.002	.961
	educationallevel	1.299	.371	12.297	1	.000	3.667
	workingstatus	-.726	.358	4.120	1	.042	.484
	montlyincome	.418	.284	2.174	1	.140	1.520
	maritalstatus	-.509	.321	2.516	1	.113	.601
	sunexposureduetoactivitiesadwork	.877	.351	6.246	1	.012	2.404
	Constant	1.218	.652	3.488	1	.062	3.380

a. Variable(s) entered on step 1: Age, educationallevel, workingstatus, montlyincome, maritalstatus, sunexposureduetoactivitiesadwork.

Logistic Regression

Case Processing Summary

Unweighted Cases[a]		N	Percent
Selected Cases	Included in Analysis	384	100.0
	Missing Cases	0	.0
	Total	384	100.0
Unselected Cases		0	.0
Total		384	100.0

a. If weight is in effect, see classification table for the total number of cases.

Dependent Variable Encoding

Original Value	Internal Value
No	0

Yes	1

Classification Table[a,b]

Observed			Predicted: useofsunscreen No	Predicted: useofsunscreen Yes	Percentage Correct
Step 0	useofsunscreen	No	0	94	.0
		Yes	0	290	100.0
	Overall Percentage				75.5

a. Constant is included in the model.

b. The cut value is .500

Variables in the Equation

		B	S.E.	Wald	df	Sig.	Exp(B)
Step 0	Constant	1.127	.119	90.100	1	.000	3.085

Variables not in the Equation

			Score	df	Sig.
Step 0	Variables	Age	23.905	1	.000
		educationallevel	23.551	1	.000
		workingstatus	6.876	1	.009
		montlyincome	.058	1	.810
		maritalstatus	16.934	1	.000
		sunexposureduetoactivitiesadwork	8.869	1	.003
	Overall Statistics		49.204	6	.000

Omnibus Tests of Model Coefficients

		Chi-square	df	Sig.
Step 1	Step	46.795	6	.000
	Block	46.795	6	.000
	Model	46.795	6	.000

Model Summary

Step	-2 Log likelihood	Cox & Snell R Square	Nagelkerke R Square
1	380.629[a]	.115	.171

a. Estimation terminated at iteration number 4 because parameter estimates changed by less than .001.

Classification Table[a]

Observed			Predicted useofsunscreen No	Predicted useofsunscreen Yes	Predicted Percentage Correct
Step 1	useofsunscreen	No	20	74	21.3
		Yes	11	279	96.2
	Overall Percentage				77.9

a. The cut value is .500

Variables in the Equation

		B	S.E.	Wald	df	Sig.	Exp(B)	95% Lowe
Step 1[a]	Age	-.040	.013	9.668	1	.002	.961	[illegible]
	educationallevel	1.299	.371	12.297	1	.000	3.667	[illegible]
	workingstatus	-.726	.358	4.120	1	.042	.484	[illegible]
	montlyincome	.418	.284	2.174	1	.140	1.520	[illegible]
	maritalstatus	-.509	.321	2.516	1	.113	.601	[illegible]
	sunexposureduetoactivitiesad work	.877	.351	6.246	1	.012	2.404	[illegible]
	Constant	1.218	.652	3.488	1	.062	3.380	

a. Variable(s) entered on step 1: Age, educationallevel, workingstatus, montlyincome, maritalstatus, sunexposureduetoactivitiesadwork.

Regression

Variables Entered/Removed[a]

Model	Variables Entered	Variables Removed	Method
1	Age[b]	.	Enter

a. Dependent Variable: score

b. All requested variables entered.

Model Summary

Model	R	R Square	Adjusted R Square	Std. Error of the Estimate
1	.149[a]	.022	.020	1.034

a. Predictors: (Constant), Age

ANOVA[a]

Model		Sum of Squares	df	Mean Square	F	Sig.
1	Regression	9.308	1	9.308	8.708	.003[b]
	Residual	408.317	382	1.069		
	Total	417.625	383			

a. Dependent Variable: score

b. Predictors: (Constant), Age

Coefficients[a]

Model		Unstandardized Coefficients		Standardized Coefficients	t	Sig.
		B	Std. Error	Beta		
1	(Constant)	4.675	.144		32.570	.000
	Age	-.012	.004	-.149	-2.951	.003

a. Dependent Variable: score

Regression

Variables Entered/Removed[a]

Model	Variables Entered	Variables Removed	Method
1	workingstatus[b]	.	Enter

a. Dependent Variable: score

b. All requested variables entered.

Model Summary

Model	R	R Square	Adjusted R Square	Std. Error of the Estimate
1	.142[a]	.020	.018	1.035

a. Predictors: (Constant), workingstatus

ANOVA[a]

Model		Sum of Squares	df	Mean Square	F	Sig.
1	Regression	8.474	1	8.474	7.912	.005[b]
	Residual	409.151	382	1.071		
	Total	417.625	383			

a. Dependent Variable: score

b. Predictors: (Constant), workingstatus

Coefficients[a]

Model		Unstandardized Coefficients		Standardized Coefficients	t	Sig.
		B	Std. Error	Beta		
1	(Constant)	4.053	.097		41.810	.000
	workingstatus	.325	.116	.142	2.813	.005

a. Dependent Variable: score

Regression

Variables Entered/Removed[a]

Model	Variables Entered	Variables Removed	Method
1	Age[b]	.	Enter

a. Dependent Variable: side effects knowledge score

b. All requested variables entered.

Model Summary

Model	R	R Square	Adjusted R Square	Std. Error of the Estimate
1	.126[a]	.016	.013	1.859

a. Predictors: (Constant), Age

ANOVA[a]

Model		Sum of Squares	df	Mean Square	F	Sig.
1	Regression	21.329	1	21.329	6.174	.013[b]
	Residual	1319.606	382	3.454		
	Total	1340.935	383			

a. Dependent Variable: side effects knowledge score

b. Predictors: (Constant), Age

Coefficients[a]

Model		Unstandardized Coefficients		Standardized Coefficients	t	Sig.
		B	Std. Error	Beta		
1	(Constant)	5.943	.258		23.029	.000
	Age	-.019	.007	-.126	-2.485	.013

a. Dependent Variable: side effects knowledge score
